# Genetic Diversity and Selection of *MHC I-UAA* in Clariid Catfish from Thailand: Implications for Breeding and Conservation

**DOI:** 10.3390/genes16091106

**Published:** 2025-09-18

**Authors:** Ton Huu Duc Nguyen, Piangjai Chalermwong, Chananya Patta, Wattanawan Jaito, Worapong Singchat, Thitipong Panthum, Trifan Budi, Kednapat Sriphairoj, Sittichai Hatachote, Prapansak Srisapoome, Narongrit Muangmai, Darren K. Griffin, Agostinho Antunes, Prateep Duengkae, Kornsorn Srikulnath

**Affiliations:** 1 Animal Genomics and Bioresource Research Unit (AGB Research Unit), Faculty of Science, Kasetsart University, 50 Ngamwongwan, Chatuchak, Bangkok 10900, Thailand; nguyenhuuduc.t@ku.th (T.H.D.N.); d.k.griffin@kent.ac.uk (D.K.G.);; 2 Interdisciplinary Graduate Program in Bioscience, Faculty of Science, Kasetsart University, 50 Ngamwongwan, Chatuchak, Bangkok 10900, Thailand; 3 Faculty of Biology Education, School of Education, Can Tho University, 3/2 Street, Ninh Kieu Ward, Can Tho 900000, Vietnam; 4 Special Research Unit for Wildlife Genomics (SRUWG), Department of Forest Biology, Faculty of Forestry, Kasetsart University, 50 Ngamwongwan, Chatuchak, Bangkok 10900, Thailand; 5 School of Agricultural Technology, King Mongkut’s Institute of Technology Ladkrabang, Bangkok 10520, Thailand; 6 Faculty of Natural Resources and Agro-Industry, Kasetsart University Chalermphrakiat Sakon Nakhon Province Campus, Sakon Nakhon 47000, Thailand; kednapat.sr@ku.th (K.S.);; 7 Department of Aquaculture, Faculty of Fisheries, Kasetsart University, Bangkok 10900, Thailand; 8 Department of Fishery Biology, Faculty of Fisheries, Kasetsart University, Bangkok 10900, Thailand; 9 School of Natural Sciences, University of Kent, Canterbury CT2 7NJ, UK; 10 Interdisciplinary Centre of Marine and Environmental Research, Terminal de Cruzeiros do Portode Leixões, University of Porto, 4450-208 Porto, Portugal; 11 Department of Biology, Faculty of Sciences, University of Porto, 4169-007 Porto, Portugal; 12 Department of Genetics, Faculty of Science, Kasetsart University, Chatuchak, Bangkok 10900, Thailand; 13 Biodiversity Center, Kasetsart University (BDCKU), Bangkok 10900, Thailand

**Keywords:** aquaculture genetics, *Clarias* catfish, genetic diversity, *MHC* class I, positive selection

## Abstract

**Background/Objectives:** Understanding variabilities in the Major Histocompatibility Complex class I (*MHC I*) gene is essential for evaluating immunogenetic diversity in clariid catfish. *MHC I* plays a critical role in immune defense by presenting endogenous antigens to cytotoxic T cells. Therefore, we aimed to investigate the genetic diversity, selection patterns, and phylogenetic relationships of *MHC I* alleles in three important clariid catfish species (*Clarias gariepinus*, *Clarias macrocephalus*, and *Clarias batrachus*) across wild and hatchery populations in Thailand. **Methods:** Targeted next-generation sequencing of a 174 bp fragment partial exon 6 of *MHC I-UAA* gene was performed, along with phylogenetic analyses, neutrality tests and *dN*/*dS* analyses. **Results:** Overall, 91 novel alleles were identified in 674 individuals, all of which were novel (100% novelty), with none matching existing reference sequences, thereby revealing extensive variation in population-specific variants. Phylogenetic analyses revealed allele sharing among species, which was consistent with balanced selection. Neutrality tests and *dN*/*dS* analyses provided evidence of both purifying and diversifying selection, with episodic positive selection detected at multiple codon sites associated with the antigen-binding α1 domain. Distinct selection patterns among populations, influenced by local environmental conditions and human pressures, along with high allele richness, are reflected in the diversity of immunogenetic variations. **Conclusions:** These findings provide critical insights into immune adaptation and highlight the potential of *MHC I* as a functional marker for genetic monitoring. Although a causal relationship between *MHC I* polymorphism and disease resistance is debated, studies suggest associations with pathogen survival, indicating future implications for aquaculture breeding and conservation, particularly in marker-assisted selection for broodstock management in Thailand.

## 1. Introduction

Aquaculture is recognized as the fastest-growing sector in global animal food production, supplying more than half of the fish consumed worldwide. Among the species of farmed fish, clariid catfish (*Clarias* spp.), favored for their rapid growth, environmental tolerance, and consumer demand, are especially important in Southeast Asia. African catfish (*C. gariepinus*) is the most widely farmed, bighead catfish (*C. macrocephalus*) is native to Southeast Asia, and walking catfish (*C. batrachus*) occurs mainly in South Asia. In Thailand, the total annual fish production, which is approximately 90,000 tons [1], is insufficient to fully supply local communities. Consequently, intensive expansion has relied on a narrow genetic base, raising concerns over reduced diversity and inbreeding in hatchery stocks [2,3]. Broodstock, often derived from few individuals and rarely replenished with wild genetics, show declining fitness and immune function over time [4,5,6]. Additionally, wild populations, are increasingly affected by pollution, salinity shifts, and habitat fragmentation, which erode local adaptations [7,8]. The rapid expansion of *C. gariepinus* farming and its hybridization with *C. macrocephalus*, combined with unregulated breeding practices, have intensified genetic mixing. As a consequence, production declined between 2013 and 2023, with reduced disease resistance likely linked to genetic outbreeding and unrecorded parental stocks [9,10,11]. Moreover, resistance traits are believed to be threatened by domestication, anthropogenic pressures, and diseases, highlighting the importance of understanding their underlying genetic resources [12].

The Major Histocompatibility Complex class I (*MHC I*) gene is a critical component of the vertebrate immune system that plays a central role in recognizing and eliminating intracellular pathogens, including viruses and certain bacteria [13,14]. As a key element in the adaptive immune response, *MHC I* encodes a transmembrane glycoprotein that presents endogenously derived peptide antigens to cytotoxic T lymphocytes, thereby initiating downstream immune reactions [15,16]. The *MHC* type I region in teleosts exhibits extensive polymorphism and enables populations to adapt to diverse and evolving pathogenic environments [17,18]. This polymorphism is maintained by balancing selection mechanisms such as heterozygote advantage, frequency-dependent selection, and fluctuating pathogen-driven pressures [19]. By contrast, variations in *MHC I* genes influence disease susceptibility in clariid catfish, affecting individual immune responses and important traits such as survival, reproduction, and resilience in high-density natural and aquaculture settings [20,21]. *MHC I* is also increasingly used as a functional genetic marker to evaluate the impact of environmental stressors, population bottlenecks, domestication, and habitat degradation [22,23]. However, unlike microsatellites, which track neutral variation, *MHC* loci directly reflect adaptive evolution [24]. *MHC I* is also considered a powerful marker in ecological and applied research, including conservation genetics and sustainable aquaculture, because of its ability to reflect adaptive variations. In hatcheries, immune genes, such as *MHC*, which are affected by reduced natural selection and increased artificial selection, can experience lowered adaptability and survival [25,26]. Furthermore, decreased *MHC* diversity, which has been linked to increased disease vulnerability and lower survival, has been observed in several freshwater fish groups such as salmonids and cyprinids [27,28].

Consequently, understanding *MHC I* variations, which are essential for designing effective breeding strategies for clariid catfish to enhance disease resistance without reducing genetic diversity, can also be used to compare wild and hatchery populations and identify population-specific alleles for genetic monitoring [17,25,29]. Despite its importance, the diversity of *MHC I* in clariid catfish and populations in Southeast Asia, including Thailand, remains poorly studied. To date, no prior comprehensive study has investigated *MHC I* diversity in Thai *Clarias* populations, representing a critical knowledge gap that impedes efficient hatchery management. This knowledge gap hampers effective hatchery management and highlights the urgent need to improve our understanding of advances in immunogenetics and resource management. Therefore, this study aimed to assess *MHC I* variations in *C. gariepinus*, *C. macrocephalus*, and *C. batrachus* in Thailand by (1) evaluating allelic diversity in wild and hatchery populations, (2) identifying signatures of natural selection, (3) reconstructing phylogenetic relationships, and (4) comparing amino acid variation and structural features.

## 2. Materials and Methods

### 2.1. Specimen Collection and DNA Extraction

A total of 674 individuals from three catfish species (246 individuals of *C. gariepinus* from five sites, 420 individuals of *C. macrocephalus* from 14 sites, and eight individuals of *C. batrachus* from one site) were collected from 20 sampling sites (wild and hatchery) across Thailand (Tables S1 and S2, Figure S1). Tissue samples (approximately 0.3 × 0.3 cm) were excised from the caudal fins of individual specimens and preserved in 1.5 mL microcentrifuge tubes containing 95% ethanol, followed by storage at 4 °C until subsequent analyses. Caudal fins were chosen because sampling causes minimal harm to the fish and these tissues have a natural ability to regenerate. Samples were collected with prior consent from fish farm owners or relevant authorities, and all individuals were immediately returned to their original habitats after sampling. Wild fish were sampled non-lethally by fin clipping. After recovery, individuals were released at their site of capture. No wild fish were retained in the laboratory. All experimental procedures, including animal care, were reviewed and approved by the Animal Experiment Committee of Kasetsart University (approval no. ACKU65-SCI-003, ACKU66-SCI-006, and ACKU66-SCI-014). These procedures were conducted in accordance with the Regulations on Animal Experiments of Kasetsart University ARRIVE guidelines (https://arriveguidelines.org/, accessed on 6 May 2025). Genomic DNA was extracted using the standard salting-out method, as described by Supikamolseni [30]. The quality and quantity of DNA were evaluated using 1% agarose gel electrophoresis and a NanoDrop 2000 Spectrophotometer (Thermo Fisher Scientific, Wilmington, DE, USA). DNA concentrations were further quantified using a Qubit 4.0 Fluorometer (Invitrogen, Carlsbad, CA, USA) to ensure accurate measurements.

### 2.2. PCR Amplification and IlluminaTM Short-Read Sequencing

A partial fragment of exon 6 of the *MHC I-UAA* gene, which contains single nucleotide polymorphisms previously associated with immune traits in teleost, was amplified using the primer pair 18MHCI_catfish_F (5′-GGTGTGGGTATACTGATGGGTC-3′) and 18MHCI_catfish_R (5′-TCAGGTAGTCCTCTGTTCCCTT-3′) [31], yielding an expected product size of 174 bp. Eight bp sample-specific barcode sequences were added to the 5′-end of each forward primer (Macrogen Inc., Seoul, Republic of Korea). PCR amplification was performed in a 15 µL reaction containing 50 ng of genomic DNA, 1× Apsalagen reaction buffer, 1.5 mM MgCl_2_, 0.2 mM dNTPs, 0.5 µM of each primer, and 0.5 U of Taq DNA polymerase (Apsalagen Co., Ltd., Bangkok, Thailand). The thermal cycling protocol included an initial denaturation at 94 °C for 10 min, followed by 35 cycles of denaturation at 94 °C for 30 s, annealing at 57 °C for 30 s, and extension at 72 °C for 1 min, with a final extension at 72 °C for 10 min. The PCR products were visualized by electrophoresis using a 1.5% agarose gel. Each sample was processed in triplicate to minimize allelic dropouts and ensure amplification reliability. A total of 92 samples per pool set with each barcode primer were pooled into nine sets and sent for paired-end short-read sequencing on the Illumina NovaSeq^TM^ 6000 platform (Novogene Co., Ltd., Singapore, Singapore). This pooling strategy provided sufficient sequencing depth, yielding an average coverage of >100× per allele after quality filtering.

### 2.3. Sequence Quality Control and Read Processing

FastQC version 0.11.9 was used to evaluate the quality of the 174 bp paired-end reads [32]. Reads with Phred scores (q) > 20 were retained for downstream analyses, as recommended for high-throughput amplicon sequencing to ensure reliable base calls [33]. Libraries were prepared for each individual without pooling, and unique dual indices were used for multiplexing. After filtering, each individual had on average ~3500 reads for the target amplicon, with a range of 1200–5000 reads, ensuring sufficient coverage for reliable allele detection. All subsequent processing steps were performed using AmpliSAS version 1.0 [33], which performs quality filtering, read merging, and the assignment of sequences to individual samples. Amplicons corresponding to allelic variants of *MHC I* genes were clustered, filtered, and assigned to individuals based on the number of alleles per sample [33]. A minimum amplicon depth threshold of 100 reads was used to eliminate the background noise and low-read samples, in accordance with AmpliSAS guidelines for minimizing artifacts and false allele calls [33,34]. Variants occurring at <1% frequency within an amplicon were discarded to minimize sequencing artifacts. The number of true alleles was determined using the degree of change criterion based on the sequencing depth, followed by the default configuration for other parameters [34]. The maximum number of alleles per individual was set to 10 to account for potential duplications in *MHC I* of teleosts [16]. This threshold is consistent with reports of multiple duplicated *MHC I* loci in teleost fishes, including catfish, where individuals may carry more than two alleles per locus [16,35]. Sequences were subsequently aligned and mapped to the *MHC I* reference gene sequence of *C. gariepinus* (accession number: XM53477511) and translated into amino acid sequences using Geneious Prime version 2023.0.4 (https://www.geneious.com, accessed on 6 May 2025) to check for the presence of stop codons within the exons. The *C. gariepinus* reference was selected because it represents the most complete and annotated *MHC I* sequence currently available among *Clarias* species, whereas full-length references for *C. macrocephalus* and *C. batrachus* are not available [36].

### 2.4. Genetic Diversity and Phylogenetic Analysis

Nucleotide diversity (*π*) was estimated using DnaSP version 6.12 [37]. Analysis of molecular variance (AMOVA) was performed using R version 4.1.2 [38] with the “poppr” package to investigate the genetic structure of *C. gariepinus*, *C. macrocephalus*, and *C. batrachus* populations, and statistical significance was assessed using 1000 permutations [39]. Nucleotide sequences of the *MHC I* gene from *C. gariepinus*, *C. macrocephalus*, and *C. batrachus* were retrieved from the NCBI database using the BLASTn tool (https://blast.ncbi.nlm.nih.gov/Blast.cgi, accessed on 6 May 2025) with filtering thresholds set at a sequence similarity of >70% and a query coverage of >85%. The best-fit nucleotide substitution model was selected based on the lowest Bayesian Information Criterion (BIC), as determined by ModelFinder, and the GTR + G model was applied in the subsequent analyses [40]. All retrieved *MHC I* gene sequences were subsequently used to construct a Bayesian phylogenetic tree using MrBayes in Geneious Prime version 2023.0.4 (https://www.geneious.com, accessed on 6 May 2025) [41]. Five Markov Chain Monte Carlo (MCMC) chains were run for 2 million generations, with trees sampled every 5000 generations and a burn-in of 200,000 to illustrate the evolutionary relationships among *MHC I* alleles in catfish. The resulting phylogenetic tree was visualized using Interactive Tree of Life version 5 [42] (https://itol.embl.de/, accessed on 6 May 2025).

### 2.5. Selection Analysis

Neutrality tests, including Tajima’s *D*, Fu and Li’s F*, and D* were performed using DnaSP version 6.12 [37] to assess deviations from neutrality and identify potential signals of selection at the *MHC I* locus. Analyses were conducted for each population using the respective sample sizes (Table 1). The type of selective pressure acting on *MHC I* in clariid catfish was evaluated using the non-synonymous to synonymous substitution rate ratio (*dN*/*dS* or *ω*). The average number of synonymous (*dS*) and nonsynonymous (*dN*) substitutions per site was estimated using the Nei–Gojobori method [43], with Jukes–Cantor correction, implemented in Molecular Evolutionary Genetics Analysis (MEGA) version X [44]. A value of *ω* close to 1 indicates neutral selection, *ω* > 1 suggests positive selection, and *ω* < 1 indicates purifying selection. A suite of codon-based models implemented on the Datamonkey web server (https://www.datamonkey.org, accessed on 6 June 2025) was used to detect signals of natural selection acting on individual codon sites in a partial fragment of exon 6 of *MHC I* genes. A mixed-effects model of evolution (MEME) was used to identify codons under episodic diversifying selection, in which selection acts on a subset of branches at a given site [45]. The analysis was run with a significance threshold of *p* ≤ 0.01. Fixed Effects Likelihood (FEL) was applied to detect pervasive selection by estimating the non-synonymous and synonymous substitution rates at each site across the entire phylogeny. Sites with *p* ≤ 0.01 were considered significant [46]. Fast Unconstrained Bayesian AppRoximation (FUBAR) was used to infer pervasive selection using a Bayesian framework, with posterior probability ≥ 0.9 used as the threshold for significance [47].

### 2.6. Multiple Sequence Alignment of MHC I Amino Acid Residues

Reference sequences of the *MHC I* gene in *C. gariepinus* (XM53477511, MG545605 and EU714321), *C. batrachus* (KC750210), *Silurus meridionalis* (M46842361 and M46859545), and *Tachysurus fulvidraco* (KP881737), were retrieved from the NCBI database using the BLASTp tool (https://blast.ncbi.nlm.nih.gov/Blast.cgi, accessed on 6 June 2025). BLASTp analysis was performed using filtering thresholds of >60% sequence similarity and >85% query coverage, as the study sequences had relatively low similarity to available references, and stricter cutoffs might have excluded important functional variants [48,49]. Subsequently, *MHC I* amino acid sequences from this study and the reference teleost species were aligned using ClustalW in Geneious Prime v2023.0.4 (https://www.geneious.com, accessed on 6 June 2025). The resulting alignment was trimmed to 54 amino acids, and a Bayesian phylogenetic tree was constructed as mentioned above. Secondary protein structures were predicted using EMBOSS tool version 6.5.7 (http://emboss.sourceforge.net/, accessed on 6 June 2025).

## 3. Results

### 3.1. Genetic Diversity of Catfish Based on MHC I Gene

In total, 174 bp of the *MHC I* partial fragment were obtained. Nucleotide diversity of *C. gariepinus* was 0.095 ± 0.017; *C. macrocephalus* was 0.133 ± 0.033 and *C. batrachus* was 0.087 (Table 1). The number of alleles per individual ranged from 1 to 9, with over 80% of the samples possessing between 1 and 4 alleles. The number of alleles per population ranged from 2 to 38 in *C. macrocephalus*, 3–37 in *C. gariepinus*, and six in *C. batrachus*. The highest allele richness was observed for *C. macrocephalus* from Sakon Nakhon 1 with 38 alleles, followed by *C. gariepinus* from Kalasin 2 with 37 alleles, and *C. batrachus* from Ubon Ratchathani with six alleles. Compared to the reference *MHC I* gene sequence of *C. gariepinus* (accession number: XM53477511), 82 polymorphic sites were identified in all individuals, defining 91 newly identified alleles (Table S3). These alleles accounted for 100% novel sequences, as none overlapped with previously reported alleles [36]. Notably, Clarias_MHCI*TH2 and Clarias_MHCI*TH7 were the most prevalent alleles, detected in 15 of the 20 populations. Clarias_MHCI*TH15, Clarias_MHCI*TH19, Clarias_MHCI*TH23, Clarias_MHCI*TH33, Clarias_MHCI*TH37, Clarias_MHCI*TH39, Clarias_MHCI*TH42, and Clarias_MHCI*TH43 were unique to the KSN2 population. *Clarias*_MHCI*TH62, Clarias_MHCI*TH84—Clarias_MHCI*TH88 were specific to the SKN1 population, whereas *Clarias*_MHCI*TH64, Clarias_MHCI*TH66, Clarias_MHCI*TH67, Clarias_MHCI*TH69, and Clarias_MHCI*TH70 were unique to the STN population. Meanwhile, *Clarias*_MHCI*TH72 was exclusively found in the NST2 population. Alleles Clarias_MHCI*TH74, Clarias_MHCI*TH75, Clarias_MHCI*TH77, Clarias_MHCI*TH80, and Clarias_MHCI*TH81 were specific, in the SKN2 population, whereas Clarias_MHCI*TH90 and Clarias_MHCI*TH91 were unique to SKN3 and KSN1 populations, respectively. At the species level, eight alleles were observed only in *C. gariepinus* within this dataset, whereas 18 alleles were specifically found in *C. macrocephalus*. No species-specific alleles were detected in *C. batrachus* (Table S3).

**Figure 1:**
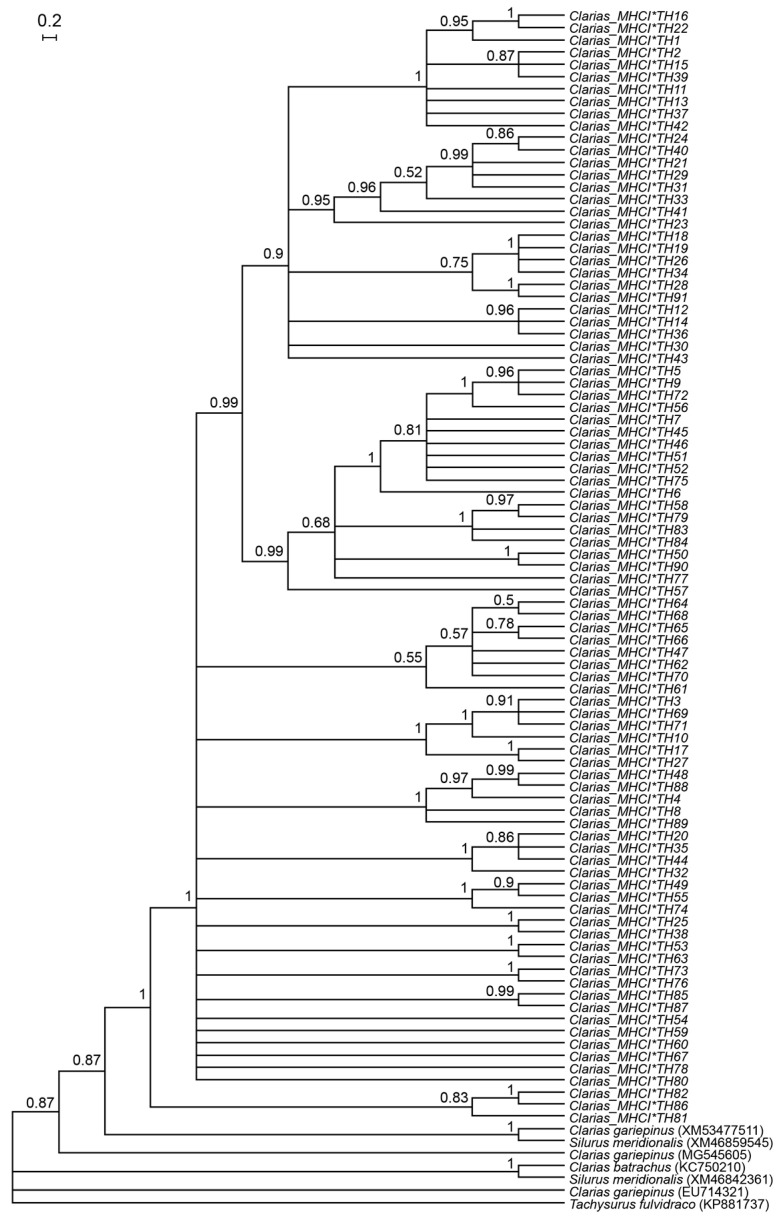
Bayesian phylogenetic tree based on *MHC I* gene in catfish species. Values above branches represent posterior probability. The scale bar indicates the number of substitutions per site. Alleles do not form species-specific clusters, reflecting allele sharing among *C. gariepinus*, *C. macrocephalus*, and *C. batrachus*. Reference sequences from *T. fulvidraco* was included as outgroups.

The Bayesian phylogenetic tree, which revealed the clustering of newly characterized alleles (*Clarias_MHCI_TH*) into distinct clades, supported the divergence of *MHC I* alleles among the three clariid and reference species (Figure 1). AMOVA indicated that 43% of the genetic variation in *MHC I* was attributable to differences among individuals (*p* < 0.001), whereas 11% was due to differences among populations (*p* = 0.02) (Table S4).

### 3.2. Selection and Selective-Sweep Analyses of Clariid Catfish

Neutrality tests of the partial *MHC* type I gene fragment revealed differences in Tajima’s *D*, and Fu and Li’s *D* and *F* values across several populations of *C. gariepinus*, *C. macrocephalus*, and *C. batrachus* (Table 2). Tajima’s *D* values ranged from −1.700–0.604, which were not statistically significant. By contrast, Fu and Li’s D* values ranged from −3.082–1.356, with significant deviations from neutrality in *C. macrocephalus* populations SNK1-CM-W, SNK2-CM-W, and SNK4-CM-C (Table 2). Similarly, Fu and Li’s F* values ranged from −2.800–1.001, with significant results in SNK1-CM-W, SNK2-CM-W, and SNK4-CM-C (Table 2). The *MHC I* gene showed an average ω value of 1.563, ranging from 0.678 to 10.000. All populations of *C. gariepinus* had *ω* > 1 (ranging from 1.750 to 3.200). Meanwhile, SNK2-CM-W, SB-CM-C, and SNK4-CM-C populations in *C. macrocephalus* exhibited *ω* > 1, while others exhibited *ω* < 1. The *ω* = 1.103 for *C. batrachus* (Table 3). Codon-level selection analysis using MEME revealed that 39 of the 54 codon sites were subjected to episodic diversifying selection (*p* < 0.01). On average, 5.08 branches per selected site exhibited evidence of diversifying selection, highlighting lineage-specific adaptive evolution (Table S5). The FEL analysis revealed evidence of selection at two codon sites within the *MHC I* gene region (Table S6). Notably, codon 49 was found to be under diversified selection (*p* < 0.01). By contrast, codon 2 was under purifying selection (*p* < 0.01), suggesting functional constraints and evolutionary conservation. Additionally, FUBAR analysis identified two sites (codons 1 and 2) evolving under pervasive purifying selection and four sites (codons 10, 12, 48, and 49) evolving under pervasive diversifying selection based on posterior probability thresholds (>0.9) and supported by Bayes Factors (Table S7).

### 3.3. Multiple Sequence Alignment of MHC I Amino Acid Residues and Prediction

**Figure 2:**
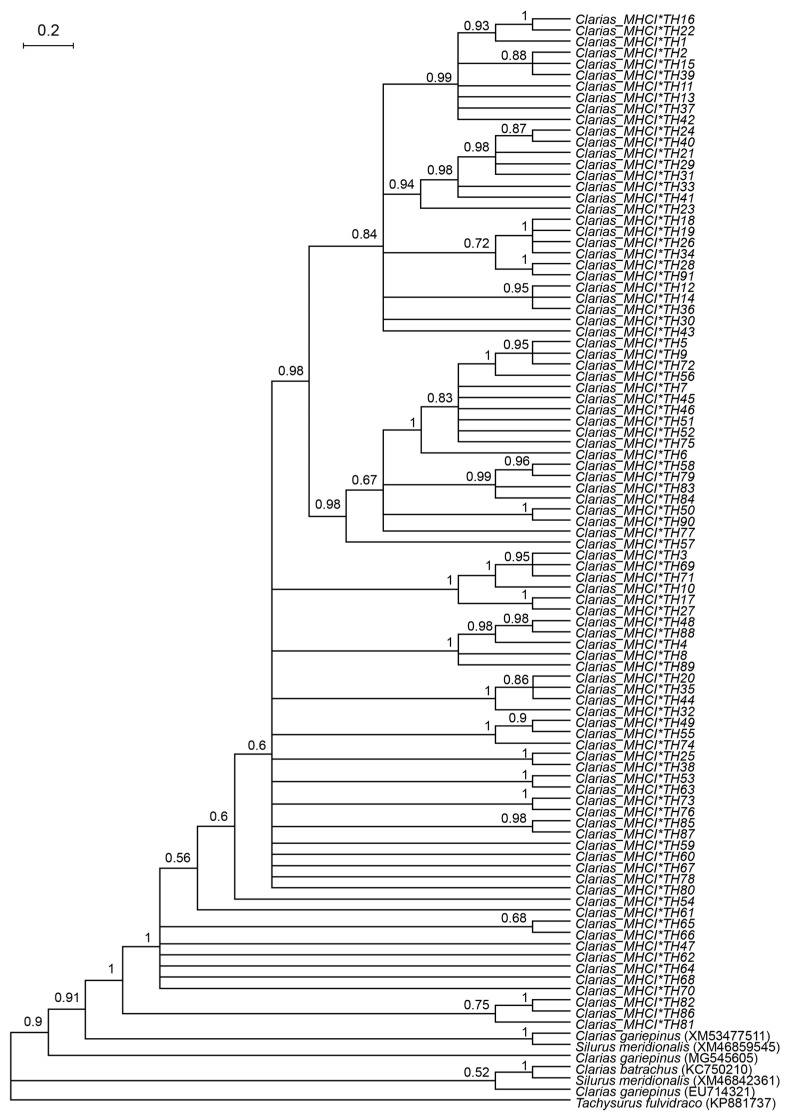
Bayesian phylogenetic tree based on *MHC I* gene amino acid residues in catfish species. Values above branches represent posterior probability. The scale bar indicates the number of substitutions per site. Alleles do not form species-specific clusters, reflecting allele sharing among *C. gariepinus*, *C. macrocephalus*, and *C. batrachus*. Reference sequences from *T. fulvidraco* was included as outgroups.

Overall, 54 amino acid residues of the partial fragment of the *MHC I* gene from 91 alleles were detected in the populations of *C. gariepinus*, *C. macrocephalus*, and *C. batrachus.* They showed low similarity with those of *C. gariepinus* (XM53477511, MG545605 and EU714321; range 6.45–38.71%), *C. batrachus* (KC750210; range 6.45–35.48%), *S. meridionalis* (M46842361 and M46859545; range 6.45–32.26%), and *T. fulvidraco* (KP881737; range 16.12–32.87%). The very low similarity values reflect the high polymorphism characteristic of *MHC I* genes, where strong diversifying selection maintains extensive amino acid diversity to enhance pathogen recognition. These alleles primarily encode amino acids located in the α1 domain of the *MHC I* molecule (Figure S2). In total, 258 mutations were identified, including 26 silent, 215 missense, and 17 nonsense mutations (Table S8). Bayesian phylogenetic analysis based on the amino acid sequences of *MHC I* alleles revealed no distinct clustering patterns among the clariid catfish (Figure 2).

## 4. Discussion

### 4.1. MHC I-UAA Diversity in Clariid Catfish

This study revealed remarkable genetic diversity in exon 6 of *MHC I-UAA*, with 47 polymorphic sites and 91 novel alleles identified from a short 174 bp fragment in clariid catfish, indicating high variability compared to *Salmo salar* (10–20 alleles), *Cyprinus carpio* (5–10 alleles), and *Oreochromis niloticus* (31 alleles), which have fewer alleles [16,27,50]. This is consistent with previous studies reporting that a large allelic diversity of *MHC* genes has been observed in clariid catfish, which are known for their exceptional environmental adaptability and broad pathogen resistance among teleosts [16,17,19,51,52,53]. Among the three clariid catfish evaluated in this study, *C. macrocephalus*, which exhibited the highest allele diversity with 59 alleles in 14 populations, is linked to its larger sampling sites, which are subject to varying selection pressures in hatchery and wild environments. However, from a phylogenetic perspective, no species-specific clade or clustering was observed in the *MHC I-UAA* gene sequences based on nucleotide and amino acid data. This allele sharing and lack of clear clustering may be influenced by recent divergence times, which are less than 20 million years, allowing for the retention of ancestral polymorphisms [54]. Additionally, shared alleles across species, such as *Clarias_MHCITH12* and *Clarias_MHCITH34*, may result from historical introgression, which are associated with balanced selection at immune loci, such as *MHC* [55,56]. The persistence of identical or highly similar alleles across species boundaries is a hallmark of trans-species polymorphism, whereby balancing selection maintains ancient allelic lineages over extended evolutionary timescales despite speciation events [19]. Similar interspecific allele sharing, which is maintained across species due to long-term balancing selection, has been observed in other teleosts such as salmonids (*S. salar*, *Oncorhynchus mykiss*, and *Oncorhynchus kisutch*) and *Etheostoma* species (*Etheostoma flabellare*, *Etheostoma caeruleum*, and *Etheostoma spectabile*) [57,58]. Thus, evolutionary proximity supports the plausibility of shared *MHC I* alleles, despite their morphological and ecological differences. The newly identified *MHC I-UAA* alleles in this study demonstrated relatively low amino acid similarity to reference sequences and closely related species, such as *C. batrachus*, *S. meridionalis*, and *T. fulvidraco*. This suggests that they either follow independent evolutionary paths or persist as ancient, highly divergent lineages that have not been eliminated by selection [59]. Moreover, several amino acid substitutions, especially at residues forming β-strands and α-helices in the α1 domain, which are important for maintaining the structure of the peptide-binding groove, were detected in this study. This groove, which directly binds to endogenous peptides and presents them to cytotoxic T cells, is critical for immune function [15,18]. Thus, the substitutions, which may alter the local secondary structure of the peptide-binding groove and, in turn, affect folding stability and peptide-binding capacity of *MHC I* molecules, with possible consequences for pathogen recognition [60,61]. In addition to the high allelic diversity and trans-species polymorphisms observed, several experimental studies in *C. gariepinus* have demonstrated functional links between *MHC I* variation and disease resistance. It was shown by Azis et al. [20] and Alimuddin et al. [62] that offspring with specific *MHC I* markers had significantly higher survival rates after *Aeromonas hydrophila* infection. Imron et al. [31] identified a unique allele, Clg-UAA*07, amplified only in resistant individuals, suggesting a direct connection between *MHC I* polymorphism and pathogen survival. Additionally, Oyebola et al. reported differential expression of *MHC I* in challenged *C. gariepinus*, further supporting its role in immune response. Although the causal relationship remains debated [18], these findings imply that *MHC I* diversity could enhance resilience against pathogens and serve as a basis for marker-assisted selection in aquaculture.

### 4.2. Evidence of Selection at the MHC I-UAA Locus

Positive selection acting on the *MHC I*-*UAA* locus was supported by high ω values and codon-level evidence from MEME, FEL, and FUBAR analyses; however, allelic frequency patterns did not indicate dominance by one or two alleles. This suggests that positive selection may not act through selective sweeps favoring specific alleles but rather through mechanisms such as heterozygous advantage or fluctuating pathogen pressures that maintain diversity. Unlike a selective sweep, where one or a few alleles rapidly rise to dominance, balancing selection preserves multiple alleles within populations [19]. Additionally, results of Fu and Li’s neutrality tests further support the recent or ongoing selection with selective sweeps and population expansion, as evidenced by significantly negative Fu and Li’s D* and F* values in several *C. macrocephalus* populations. By contrast, Tajima’s *D* test did not reach statistical significance overall. Nevertheless, these *ω* values could reflect localized episodic selection without fixation, especially in hatchery populations, which are subjected to environmental instability and artificial selection [16,19]. Moreover, the conditions described may influence the selection regime acting on immune genes, where fluctuating or relaxed selection can temporarily increase *ω* without causing long-term fixation. For instance, population-specific alleles associated with localized environmental factors, limited gene flow, and demographic history suggest effects of domestication, artificial selection, or genetic drift from restricted broodstock diversity. These alleles may also indicate local adaptation, especially if linked to immune regions such as the *MHC*, shaped by pathogen communities, environmental pressures, and management practices in aquaculture. For practical breeding, such population-specific alleles can guide conservation of genetically diverse stocks (e.g., SNK2-CM-W) that serve as valuable reservoirs for future broodstock improvement and disease-resilient breeding programs. Such environments often exhibit directional and episodic selection owing to exposure to fluctuating or novel pathogens [3,5,23,63,64]. For example, strong diversifying selection for *MHC I* codons is observed in *S. salar* in pathogen-rich environments, whereas diversifying selection for *MHC I* codons is linked to environmental stressors, such as temperature and pollution in *O. niloticus* [17,50,65]. Furthermore, variation in selective pressures was observed among clariid catfish populations. For instance, the wild population SNK2-CM-W showed strong positive selection and significant neutrality test results, while NST1-CM-W and NPT2-CM-C showed *ω* < 1, indicating purifying selection. These patterns may be associated with environmental and management differences between populations. Potential factors include variation in pathogen exposure, water quality, and hatchery conditions (e.g., selective breeding and stocking density), although direct evidence for these effects is not yet available. Subsequently, using the MEME method, 39 of the 54 codons were identified as being under episodic diversifying selection, indicating that positive selection acted at different sites along various branches of the phylogeny. This proportion is unusually high compared with reports in other teleosts, where only a smaller subset of codons shows such signals, bringing attention to the strong and widespread selective pressures acting on clariid catfish *MHC* [50,65]. This pattern supports the occurrence of lineage-specific adaptations driven by population-specific immune challenges [45]. Additionally, strong selection signals were observed at codons 10, 12, 48, and 49 within the α1 domain of the *MHC I* molecule, which is involved in antigen recognition. Secondary structure prediction revealed that this domain is mainly composed of β-strands, α-helices, and coil regions, indicating a well-organized and stable structure typical of antigen-binding areas (Figure S3). Using FEL and FUBAR, which detect pervasive selection, codons 48 and 49 were identified, whereas only codons 10 and 12 were identified by MEME under episodic diversifying selection. This is consistent with the findings in teleosts, where MEME typically detects more positively selected sites than FEL and FUBAR [50,65]. These results, which reflect method-specific sensitivity, showed that MEME detects selection acting on a subset of branches (episodic), whereas FEL and FUBAR detect selection acting across the entire phylogeny (pervasive). These codons, which are present in multiple alleles such as Clarias_MHCI*TH12, Clarias_MHCI*TH34, Clarias_MHCI*TH48, and Clarias_MHCI*TH84, with nonsynonymous substitutions, are considered potential candidates for further functional studies [66,67]. Codons 10 and 12 are positioned at β-strand regions, while codons 48 and 49 are located within the α-helix of the antigen-binding α1 domain, suggesting potential influence on peptide-binding interactions [68].

### 4.3. Advances and Future Directions in MHC Genotyping

*MHC I* in clariid catfish, which is shaped by heterogeneous and largely episodic selection, particularly in hatcheries or environmentally unstable settings, reflects the influence of various selective pressures [17,19,31,69]. However, our findings provide novel insight into the evolutionary dynamics of *MHC I* in clariid catfish, which also suggest that high diversity and population-specific alleles reflect immune adaptation to local pathogens, supporting disease-resistant breeding strategies [19,24]. Functional markers, such as *MHC I* are used to enable more accurate assessments of immune potential than neutral markers in disease-prone high-density aquaculture systems [22]. A key application involves integrating beneficial *MHC* alleles into selective breeding programs to enhance disease resistance while maintaining genetic diversity. However, overemphasis on only a few alleles could inadvertently narrow overall diversity, increasing inbreeding risks and reducing long-term adaptability of farmed populations. Therefore, conservation of broad allele pools alongside targeted selection is essential [70]. Populations with unique alleles or strong positively selection signals (*ω* = 10.000), like SNK2-CM-W, may be prioritized as “immune variability reservoirs” [71]. SNK1-CM-W and SNK2-CM-W, which exhibited high allelic richness and private alleles, are valued for their immunogenetic diversity and are considered promising for broodstock conservation. Despite the advances in sequencing and selection models, this study focused on short *MHC I* segments. Consequently, further research, including expression assays and immune challenge experiments, is required to clarify the functional roles of these novel alleles [16,72]. Future studies could also apply RNA-seq to quantify allele-specific expression patterns under pathogen stress, providing direct evidence of functional relevance. For instance, long-read sequencing, such as PacBio or Nanopore, can be used to resolve full *MHC* loci, revealing gene copy variations and regulatory complexities that are often missed by short amplicon approaches [33]. Nevertheless, this study enhances our understanding of *MHC* class I evolution in clariid catfish and provides a genetic basis for disease-resistant breeding and conservation, thereby supporting the integration of *MHC* data into broodstock management for sustainable aquaculture.

## 5. Conclusions

To the best of our knowledge, this study provides the first comprehensive analysis of *MHC I-UAA* gene variation in three important clariid catfish species in Thailand using targeted next-generation sequencing. We identified 91 novel alleles across wild and hatchery populations, revealing substantial diversity in exon 6, which encodes the antigen-binding α1 domain. Purifying and diversifying selections, with population- and site-specific signals of adaptive evolution, were detected by molecular analyses, highlighting dynamic immune gene responses to environmental and pathogenic pressures. These findings support the use of population-specific alleles as MAS targets to improve disease resistance. Populations such as SNK2-CM-W and SNK1-CM-W, which exhibited high allelic richness and unique variants, are considered promising sources for marker-assisted selection in breeding programs. These genetic patterns provide a basis for defining populations that can be used for genetic monitoring and management. Future studies, including functional validation and full-length *MHC* genotyping through long-read sequencing, are expected to further enhance our understanding of adaptive immune variation and support the development of more effective breeding and conservation strategies for tropical freshwater fishes. In particular, disease-challenge experiments will be essential to validate whether specific alleles provide measurable resistance when exposed to pathogens.

## Figures and Tables

**Table 1:** Nucleotide sequence diversity in catfish populations based on *MHC I* sequences.

Species	Population	Code	*N*	Number of Allele per Population	Nucleotide Diversity
*C. gariepinus*	Nakhon Nayok	NYK-CG-C	5	3	0.074
	Kalasin 1	KSN1-CG-C	94	20	0.111
	Kalasin 2	KSN2-CG-C	134	37	0.082
	Ubon Ratchathani	UBR-CG-C	6	3	0.091
	Sing Buri	SB-CG-C	7	7	0.118
	*C. gariepinus*	-	246	41	0.095
	SD	-	-	-	0.017
*C. macrocephalus*	Sing Buri	SB-CM-C	4	4	0.240
	Sakon Nakhon 1	SNK1-CM-W	182	38	0.133
	Sakon Nakhon 2	SNK2-CM-W	74	34	0.121
	Sakon Nakhon 3	SNK3-CM-W	82	16	0.104
	Sakon Nakhon 4	SNK4-CM-C	14	5	0.132
	Suphan Buri 1	SPB1-CM-W	6	11	0.123
	Suphan Buri 2	SPB2-CM-W	3	10	0.127
	Nakhon Pathom 1	NPT1-CM-W	2	8	0.131
	Nakhon Pathom 2	NPT2-CM-W	2	7	0.144
	Nakhon Si Thammarat 1	NST1-CM-W	3	6	0.145
	Nakhon Si Thammarat 2	NST2-CM-C	10	13	0.136
	Surat Thani	STN-CM-C	25	26	0.114
	Nakhon Phanom	NKPN-CM-C	7	4	0.084
	Ubon Ratchathani	UBR-CM-C	6	10	0.128
	*C. macrocephalus*	-	420	59	0.133
	SD	-	-	-	0.033
*C. batrachus*	Ubon Ratchathani	UBR-CB-C	8	6	0.087
	Overall mean value	-	674	91	0.121
	SD	-	-	-	0.034

SD: standard deviation.

**Table 2:** Neutrality tests of *MHC I* gene sequences in catfish populations.

Species	Population	Code	Tajima’s *D*	Fu and Li’s *D*	Fu and Li’s *F*
*C. gariepinus*	Nakhon Nayok	NYK-CG-C	0.604 ^ns^	0.750 ^ns^	0.792 ^ns^
	Kalasin 1	KSN1-CG-C	0.228 ^ns^	1.356 ^ns^	1.001 ^ns^
	Kalasin 2	KSN2-CG-C	−0.699 ^ns^	0.045 ^ns^	−0.388 ^ns^
	Ubon Ratchathani	UBR-CG-C	−0.507 ^ns^	−0.683 ^ns^	−0.716 ^ns^
	Sing Buri	SB-CG-C	−0.869 ^ns^	−0.957 ^ns^	−1.064 ^ns^
	Mean	-	−0.249	0.102	−0.075
*C. macrocephalus*	Sing Buri	SB-CM-C	−1.292 ^ns^	−1.188 ^ns^	−1.330 ^ns^
	Sakon Nakhon 1	SNK1-CM-W	−0.936 ^ns^	−3.082 *	−2.392 *
	Sakon Nakhon 2	SNK2-CM-W	−1.142 ^ns^	−2.370 *	−2.114 ^ns^
	Sakon Nakhon 3	SNK3-CM-W	−0.032 ^ns^	0.336 ^ns^	0.206 ^ns^
	Sakon Nakhon 4	SNK4-CM-C	−1.700 ^ns^	−2.704 *	−2.800 *
	Suphan Buri 1	SPB1-CM-W	−0.721 ^ns^	0.012 ^ns^	−0.247 ^ns^
	Suphan Buri 2	SPB2-CM-W	−0.521 ^ns^	0.607 ^ns^	0.326 ^ns^
	Nakhon Pathom 1	NPT1-CM-W	−0.614 ^ns^	0.112 ^ns^	−0.085 ^ns^
	Nakhon Pathom 2	NPT2-CM-W	−0.800 ^ns^	−0.347 ^ns^	−0.505 ^ns^
	Nakhon Si Thammarat 1	NST1-CM-W	−0.085 ^ns^	0.613 ^ns^	0.495 ^ns^
	Nakhon Si Thammarat 2	NST2-CM-C	−0.542 ^ns^	−0.727 ^ns^	−0.786 ^ns^
	Surat Thani	STN-CM-C	−0.935 ^ns^	1.288 ^ns^	0.404 ^ns^
	Nakhon Phanom	NKPN-CM-C	−0.879 ^ns^	−0.906 ^ns^	−1.010 ^ns^
	Ubon Ratchathani	UBR-CM-C	−1.478 ^ns^	−1.495 ^ns^	−1.713 ^ns^
	Mean	-	−0.834	−0.704	−0.825
*C. batrachus*	Ubon Ratchathani	UBR-CB-C	−0.670 ^ns^	−1.453 ^ns^	−1.422 ^ns^
	Overall mean value	-	−0.679	−0.540	−0.667

ns: not significant; *: *p* < 0.05.

**Table 3:** Rates of synonymous (*dS*) and nonsynonymous (*dN*) substitutions in nucleotide sequences of *MHC I* gene sequences in catfish populations.

Species	Population	Code	*dN*	*dS*	*ω*
*C. gariepinus*	Nakhon Nayok	NYK-CG-C	0.049 ± 0.018	0.023 ± 0.016	2.130
	Kalasin 1	KSN1-CG-C	0.094 ± 0.020	0.037 ± 0.017	2.541
	Kalasin 2	KSN2-CG-C	0.064 ± 0.012	0.020 ± 0.008	3.200
	Ubon Ratchathani	UBR-CG-C	0.035 ± 0.013	0.011 ± 0.008	3.182
	Sing Buri	SB-CG-C	0.077 ± 0.021	0.044 ± 0.019	1.750
	Mean	-	0.064 ± 0.017	0.027 ± 0.014	2.561
*C. macrocephalus*	Sing Buri	SB-CM-C	0.502 ± 0.130	0.141 ± 0.049	3.560
	Sakon Nakhon 1	SNK1-CM-W	0.120 ± 0.023	0.090 ± 0.033	1.333
	Sakon Nakhon 2	SNK2-CM-W	0.120 ± 0.021	0.012 ± 0.037	10.000
	Sakon Nakhon 3	SNK3-CM-W	0.102 ± 0.023	0.084 ± 0.037	1.214
	Sakon Nakhon 4	SNK4-CM-C	0.200 ± 0.043	0.063 ± 0.021	3.175
	Suphan Buri 1	SPB1-CM-W	0.088 ± 0.019	0.097 ± 0.040	0.907
	Suphan Buri 2	SPB2-CM-W	0.102 ± 0.021	0.098 ± 0.043	1.041
	Nakhon Pathom 1	NPT1-CM-W	0.050 ± 0.012	0.034 ± 0.015	1.471
	Nakhon Pathom 2	NPT2-CM-W	0.066 ± 0.018	0.093 ± 0.046	0.710
	Nakhon Si Thammarat 1	NST1-CM-W	0.059 ± 0.018	0.087 ± 0.041	0.678
	Nakhon Si Thammarat 2	NST2-CM-C	0.070 ± 0.016	0.080 ± 0.037	0.875
	Surat Thani	STN-CM-C	0.122 ± 0.022	0.117 ± 0.043	1.043
	Nakhon Phanom	NKPN-CM-C	0.062 ± 0.017	0.052 ± 0.027	1.192
	Ubon Ratchathani	UBR-CM-C	0.139 ± 0.022	0.132 ± 0.049	1.053
	Mean	-	0.129 ± 0.029	0.084 ± 0.037	2.018
*C. batrachus*	Ubon Ratchathani	UBR-CB-C	0.107 ± 0.029	0.097 ± 0.052	1.103
	Overall mean value	-	0.111 ± 0.097	0.071 ± 0.039	1.563

## Data Availability

All sequences were deposited in the National Center for Biotechnology Information (NCBI) (https://www.ncbi.nlm.nih.gov/; accession number: PX243432–PX243522 (accessed on 8 September 2025)).

## Supplementary Materials

The following supporting information can be downloaded at https://www.mdpi.com/article/10.3390/genes16091106/s1, Figure S1: Distribution of sampling sites in Thailand; Figure S2: *MHC I* protein sequence alignment of the catfish populations in Thailand and reference sequences; Figure S3: *MHC I* protein secondary structure production in catfish species in Thailand; Table S1: Summary of catfish individuals sampled in this study; Table S2: The catfish specimens used in this study; Table S3: Variable sites in the sequences of *MHC I* alleles of North African and bighead catfish populations in Thailand; Table S4:The results of Analysis of Variance (AMOVA) for catfish populations in Thailand; Table S5: Detailed site-by-site results from the MEME analysis; Table S6: Detailed site-by-site results from the FEL analysis; Table S7: Codon sites under selection identified by FUBAR analysis; Table S8: Types of single nucleotide polymorphism (SNPs) and their locations in the partial fragment of the exon 6 of *MHC I* gene of catfish catfish populations in Thailand compare with the reference sequence (Accession number XM53477511).

## Abbreviations

The following abbreviations are used in this manuscript:

AMOVA	Analysis of Molecular Variance
BIC	Bayesian Information Criterion
ESU	Evolutionarily Significant Unit
FEL	Fixed Effects Likelihood
FUBAR	Fast Unconstrained Bayesian Approximation
MCMC	Markov Chain Monte Carlo
*MHC I*	Major Histocompatibility Complex class I
NCBI	National Center for Biotechnology Information
PCR	Polymerase Chain Reaction
