# Supplementary material for: Genetic Diversity and Selection of MHC I-UAA in Clariid Catfish from Thailand: Implications for Breeding and Conservation
Source: Genes (Basel). 2025 Sep 18;16(9):1106. doi: 10.3390/genes16091106 (PMC12470123; doi:10.3390/genes16091106)
Supplement: Supplementary file 1 [file genes-16-01106-s001.zip › genes-3877640-supplementary.pdf]

Supplementary Figure

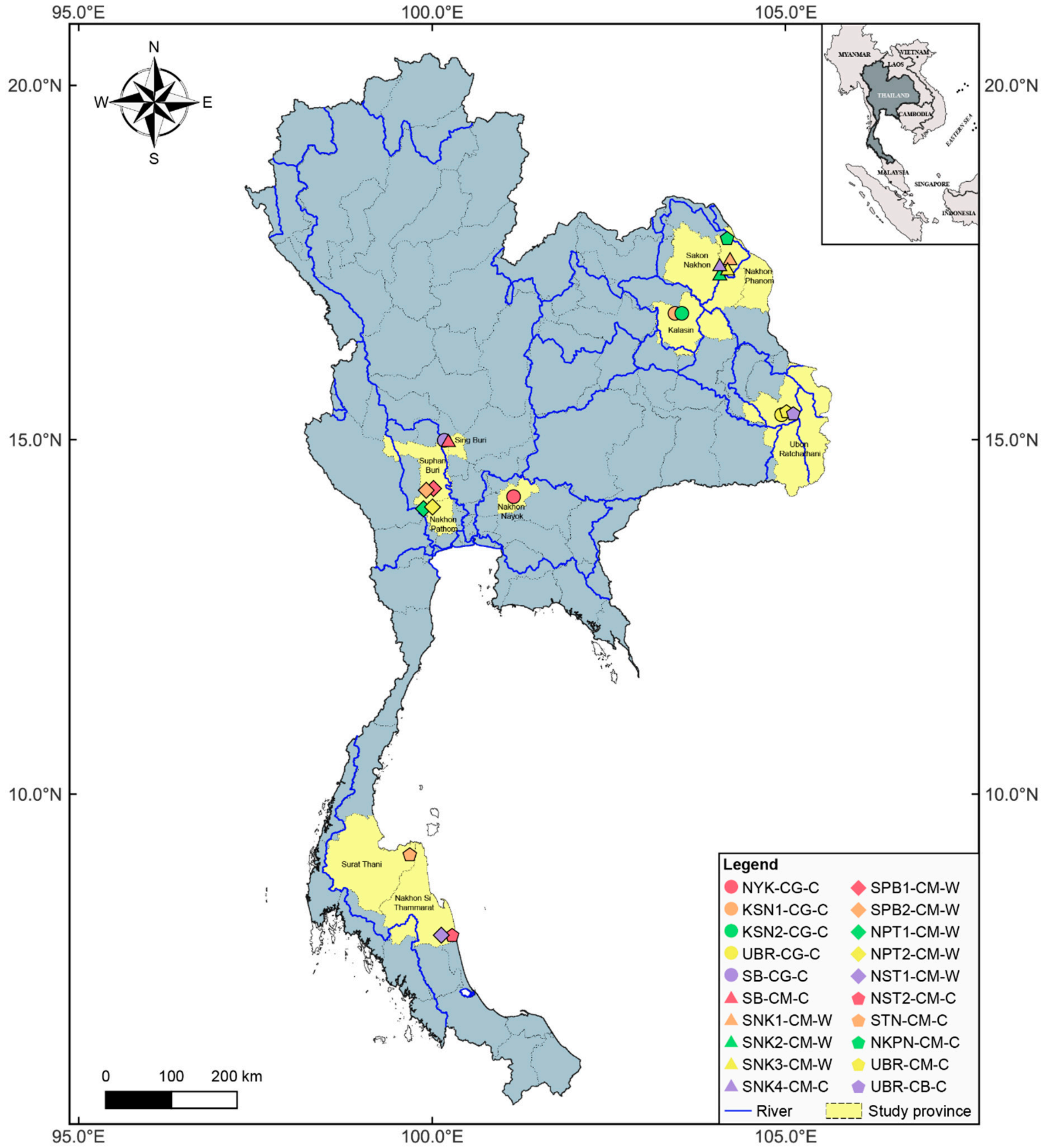

Figure S1. Distribution of sampling sites in Thailand.

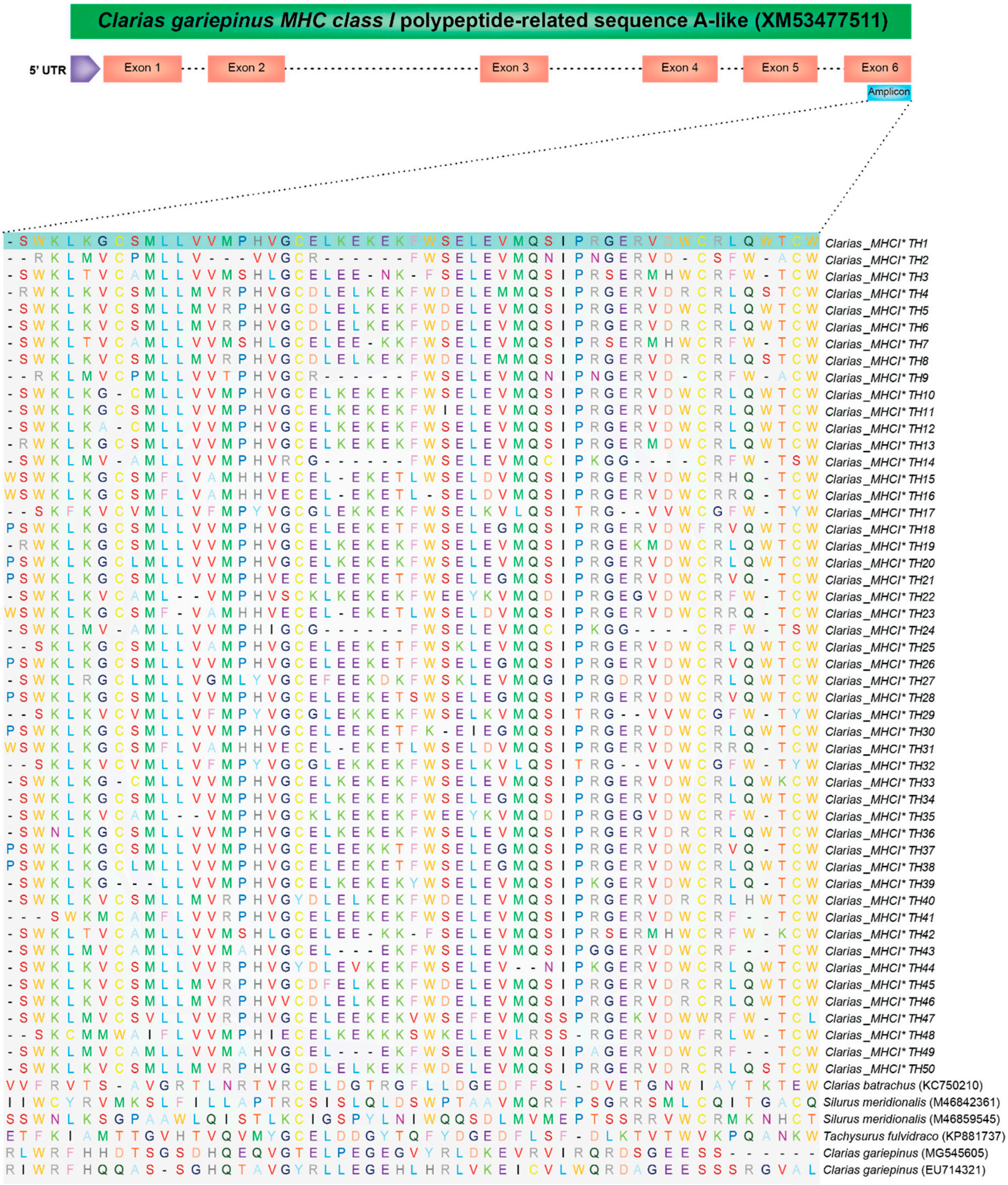

Figure S2. *MHC I* protein sequence alignment of the catfish populations in Thailand and reference sequences.

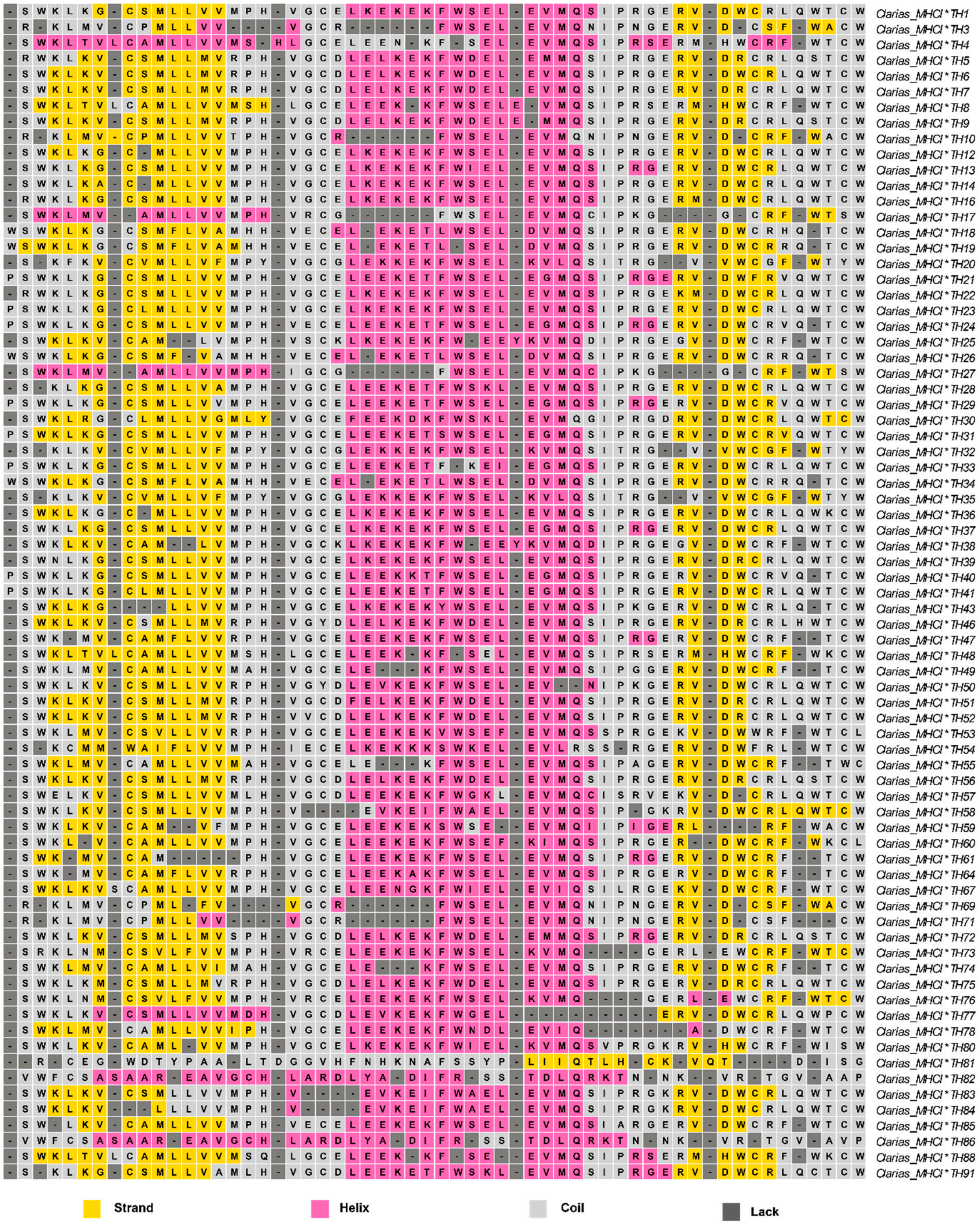

Figure S3. *MHC I* protein secondary structure production in catfish species in Thailand.

Supplementary Table

Table S1. Summary of catfish individuals sampled in this study.

No.	Abbreviation/Code	Coordinate	Locality
1	NYK-CG-C_1	14˚05'58"N, 101˚09'39"E	Nakhon Nayok
2	NYK-CG-C_2	14˚05'58"N, 101˚09'39"E	Nakhon Nayok
3	NYK-CG-C_3	14˚05'58"N, 101˚09'39"E	Nakhon Nayok
4	NYK-CG-C_4	14˚05'58"N, 101˚09'39"E	Nakhon Nayok
5	NYK-CG-C_5	14˚05'58"N, 101˚09'39"E	Nakhon Nayok
6	KSN1-CG-C_1	16°29'45.6"N, 103°25'8.9"E	Kalasin
7	KSN1-CG-C_2	16°29'45.6"N, 103°25'8.9"E	Kalasin
8	KSN1-CG-C_3	16°29'45.6"N, 103°25'8.9"E	Kalasin
9	KSN1-CG-C_4	16°29'45.6"N, 103°25'8.9"E	Kalasin
10	KSN1-CG-C_5	16°29'45.6"N, 103°25'8.9"E	Kalasin
11	KSN1-CG-C_6	16°29'45.6"N, 103°25'8.9"E	Kalasin
12	KSN1-CG-C_7	16°29'45.6"N, 103°25'8.9"E	Kalasin
13	KSN1-CG-C_8	16°29'45.6"N, 103°25'8.9"E	Kalasin
14	KSN1-CG-C_9	16°29'45.6"N, 103°25'8.9"E	Kalasin
15	KSN1-CG-C_10	16°29'45.6"N, 103°25'8.9"E	Kalasin
16	KSN1-CG-C_11	16°29'45.6"N, 103°25'8.9"E	Kalasin
17	KSN1-CG-C_12	16°29'45.6"N, 103°25'8.9"E	Kalasin
18	KSN1-CG-C_13	16°29'45.6"N, 103°25'8.9"E	Kalasin
19	KSN1-CG-C_14	16°29'45.6"N, 103°25'8.9"E	Kalasin
20	KSN1-CG-C_15	16°29'45.6"N, 103°25'8.9"E	Kalasin
21	KSN1-CG-C_16	16°29'45.6"N, 103°25'8.9"E	Kalasin
22	KSN1-CG-C_17	16°29'45.6"N, 103°25'8.9"E	Kalasin
23	KSN1-CG-C_18	16°29'45.6"N, 103°25'8.9"E	Kalasin
24	KSN1-CG-C_19	16°29'45.6"N, 103°25'8.9"E	Kalasin
25	KSN1-CG-C_20	16°29'45.6"N, 103°25'8.9"E	Kalasin
26	KSN1-CG-C_21	16°29'45.6"N, 103°25'8.9"E	Kalasin
27	KSN1-CG-C_22	16°29'45.6"N, 103°25'8.9"E	Kalasin
28	KSN1-CG-C_23	16°29'45.6"N, 103°25'8.9"E	Kalasin
29	KSN1-CG-C_24	16°29'45.6"N, 103°25'8.9"E	Kalasin
30	KSN1-CG-C_25	16°29'45.6"N, 103°25'8.9"E	Kalasin
31	KSN1-CG-C_26	16°29'45.6"N, 103°25'8.9"E	Kalasin
32	KSN1-CG-C_27	16°29'45.6"N, 103°25'8.9"E	Kalasin
33	KSN1-CG-C_28	16°29'45.6"N, 103°25'8.9"E	Kalasin
34	KSN1-CG-C_29	16°29'45.6"N, 103°25'8.9"E	Kalasin
35	KSN1-CG-C_30	16°29'45.6"N, 103°25'8.9"E	Kalasin
36	KSN1-CG-C_31	16°29'45.6"N, 103°25'8.9"E	Kalasin
37	KSN1-CG-C_32	16°29'45.6"N, 103°25'8.9"E	Kalasin
38	KSN1-CG-C_33	16°29'45.6"N, 103°25'8.9"E	Kalasin
39	KSN1-CG-C_34	16°29'45.6"N, 103°25'8.9"E	Kalasin
40	KSN1-CG-C_35	16°29'45.6"N, 103°25'8.9"E	Kalasin
41	KSN1-CG-C_36	16°29'45.6"N, 103°25'8.9"E	Kalasin
42	KSN1-CG-C_37	16°29'45.6"N, 103°25'8.9"E	Kalasin
43	KSN1-CG-C_38	16°29'45.6"N, 103°25'8.9"E	Kalasin
44	KSN1-CG-C_39	16°29'45.6"N, 103°25'8.9"E	Kalasin
45	KSN1-CG-C_40	16°29'45.6"N, 103°25'8.9"E	Kalasin
46	KSN1-CG-C_41	16°29'45.6"N, 103°25'8.9"E	Kalasin
47	KSN1-CG-C_42	16°29'45.6"N, 103°25'8.9"E	Kalasin
48	KSN1-CG-C_43	16°29'45.6"N, 103°25'8.9"E	Kalasin
49	KSN1-CG-C_44	16°29'45.6"N, 103°25'8.9"E	Kalasin
50	KSN1-CG-C_45	16°29'45.6"N, 103°25'8.9"E	Kalasin
51	KSN1-CG-C_46	16°29'45.6"N, 103°25'8.9"E	Kalasin
52	KSN1-CG-C_47	16°29'45.6"N, 103°25'8.9"E	Kalasin
53	KSN1-CG-C_48	16°29'45.6"N, 103°25'8.9"E	Kalasin

No.	Abbreviation/Code	Coordinate	Locality
54	KSN1-CG-C_49	16°29'45.6"N, 103°25'8.9"E	Kalasin
55	KSN1-CG-C_50	16°29'45.6"N, 103°25'8.9"E	Kalasin
56	KSN1-CG-C_51	16°29'45.6"N, 103°25'8.9"E	Kalasin
57	KSN1-CG-C_52	16°29'45.6"N, 103°25'8.9"E	Kalasin
58	KSN1-CG-C_53	16°29'45.6"N, 103°25'8.9"E	Kalasin
59	KSN1-CG-C_54	16°29'45.6"N, 103°25'8.9"E	Kalasin
60	KSN1-CG-C_55	16°29'45.6"N, 103°25'8.9"E	Kalasin
61	KSN1-CG-C_56	16°29'45.6"N, 103°25'8.9"E	Kalasin
62	KSN1-CG-C_57	16°29'45.6"N, 103°25'8.9"E	Kalasin
63	KSN1-CG-C_58	16°29'45.6"N, 103°25'8.9"E	Kalasin
64	KSN1-CG-C_59	16°29'45.6"N, 103°25'8.9"E	Kalasin
65	KSN1-CG-C_60	16°29'45.6"N, 103°25'8.9"E	Kalasin
66	KSN1-CG-C_61	16°29'45.6"N, 103°25'8.9"E	Kalasin
67	KSN1-CG-C_62	16°29'45.6"N, 103°25'8.9"E	Kalasin
68	KSN1-CG-C_63	16°29'45.6"N, 103°25'8.9"E	Kalasin
69	KSN1-CG-C_64	16°29'45.6"N, 103°25'8.9"E	Kalasin
70	KSN1-CG-C_65	16°29'45.6"N, 103°25'8.9"E	Kalasin
71	KSN1-CG-C_66	16°29'45.6"N, 103°25'8.9"E	Kalasin
72	KSN1-CG-C_67	16°29'45.6"N, 103°25'8.9"E	Kalasin
73	KSN1-CG-C_68	16°29'45.6"N, 103°25'8.9"E	Kalasin
74	KSN1-CG-C_69	16°29'45.6"N, 103°25'8.9"E	Kalasin
75	KSN1-CG-C_70	16°29'45.6"N, 103°25'8.9"E	Kalasin
76	KSN1-CG-C_71	16°29'45.6"N, 103°25'8.9"E	Kalasin
77	KSN1-CG-C_72	16°29'45.6"N, 103°25'8.9"E	Kalasin
78	KSN1-CG-C_73	16°29'45.6"N, 103°25'8.9"E	Kalasin
79	KSN1-CG-C_74	16°29'45.6"N, 103°25'8.9"E	Kalasin
80	KSN1-CG-C_75	16°29'45.6"N, 103°25'8.9"E	Kalasin
81	KSN1-CG-C_76	16°29'45.6"N, 103°25'8.9"E	Kalasin
82	KSN1-CG-C_77	16°29'45.6"N, 103°25'8.9"E	Kalasin
83	KSN1-CG-C_78	16°29'45.6"N, 103°25'8.9"E	Kalasin
84	KSN1-CG-C_79	16°29'45.6"N, 103°25'8.9"E	Kalasin
85	KSN1-CG-C_80	16°29'45.6"N, 103°25'8.9"E	Kalasin
86	KSN1-CG-C_81	16°29'45.6"N, 103°25'8.9"E	Kalasin
87	KSN1-CG-C_82	16°29'45.6"N, 103°25'8.9"E	Kalasin
88	KSN1-CG-C_83	16°29'45.6"N, 103°25'8.9"E	Kalasin
89	KSN1-CG-C_84	16°29'45.6"N, 103°25'8.9"E	Kalasin
90	KSN1-CG-C_85	16°29'45.6"N, 103°25'8.9"E	Kalasin
91	KSN1-CG-C_86	16°29'45.6"N, 103°25'8.9"E	Kalasin
92	KSN1-CG-C_87	16°29'45.6"N, 103°25'8.9"E	Kalasin
93	KSN1-CG-C_88	16°29'45.6"N, 103°25'8.9"E	Kalasin
94	KSN1-CG-C_89	16°29'45.6"N, 103°25'8.9"E	Kalasin
95	KSN1-CG-C_90	16°29'45.6"N, 103°25'8.9"E	Kalasin
96	KSN1-CG-C_91	16°29'45.6"N, 103°25'8.9"E	Kalasin
97	KSN1-CG-C_92	16°29'45.6"N, 103°25'8.9"E	Kalasin
98	KSN1-CG-C_93	16°29'45.6"N, 103°25'8.9"E	Kalasin
99	KSN1-CG-C_94	16°29'45.6"N, 103°25'8.9"E	Kalasin
100	KSN2-CG-C_1	16˚39’29"N, 103˚29’10"E	Kalasin
101	KSN2-CG-C_2	16˚39’29"N, 103˚29’10"E	Kalasin
102	KSN2-CG-C_3	16˚39’29"N, 103˚29’10"E	Kalasin
103	KSN2-CG-C_4	16˚39’29"N, 103˚29’10"E	Kalasin
104	KSN2-CG-C_5	16˚39’29"N, 103˚29’10"E	Kalasin
105	KSN2-CG-C_6	16˚39’29"N, 103˚29’10"E	Kalasin
106	KSN2-CG-C_7	16˚39’29"N, 103˚29’10"E	Kalasin
107	KSN2-CG-C_8	16˚39’29"N, 103˚29’10"E	Kalasin
108	KSN2-CG-C_9	16˚39’29"N, 103˚29’10"E	Kalasin
109	KSN2-CG-C_10	16˚39’29"N, 103˚29’10"E	Kalasin

No.	Abbreviation/Code	Coordinate	Locality
110	KSN2-CG-C_11	16˚39’29"N, 103˚29’10"E	Kalasin
111	KSN2-CG-C_12	16˚39’29"N, 103˚29’10"E	Kalasin
112	KSN2-CG-C_13	16˚39’29"N, 103˚29’10"E	Kalasin
113	KSN2-CG-C_14	16˚39’29"N, 103˚29’10"E	Kalasin
114	KSN2-CG-C_15	16˚39’29"N, 103˚29’10"E	Kalasin
115	KSN2-CG-C_16	16˚39’29"N, 103˚29’10"E	Kalasin
116	KSN2-CG-C_17	16˚39’29"N, 103˚29’10"E	Kalasin
117	KSN2-CG-C_18	16˚39’29"N, 103˚29’10"E	Kalasin
118	KSN2-CG-C_19	16˚39’29"N, 103˚29’10"E	Kalasin
119	KSN2-CG-C_20	16˚39’29"N, 103˚29’10"E	Kalasin
120	KSN2-CG-C_21	16˚39’29"N, 103˚29’10"E	Kalasin
121	KSN2-CG-C_22	16˚39’29"N, 103˚29’10"E	Kalasin
122	KSN2-CG-C_23	16˚39’29"N, 103˚29’10"E	Kalasin
123	KSN2-CG-C_24	16˚39’29"N, 103˚29’10"E	Kalasin
124	KSN2-CG-C_25	16˚39’29"N, 103˚29’10"E	Kalasin
125	KSN2-CG-C_26	16˚39’29"N, 103˚29’10"E	Kalasin
126	KSN2-CG-C_27	16˚39’29"N, 103˚29’10"E	Kalasin
127	KSN2-CG-C_28	16˚39’29"N, 103˚29’10"E	Kalasin
128	KSN2-CG-C_29	16˚39’29"N, 103˚29’10"E	Kalasin
129	KSN2-CG-C_30	16˚39’29"N, 103˚29’10"E	Kalasin
130	KSN2-CG-C_31	16˚39’29"N, 103˚29’10"E	Kalasin
131	KSN2-CG-C_32	16˚39’29"N, 103˚29’10"E	Kalasin
132	KSN2-CG-C_33	16˚39’29"N, 103˚29’10"E	Kalasin
133	KSN2-CG-C_34	16˚39’29"N, 103˚29’10"E	Kalasin
134	KSN2-CG-C_35	16˚39’29"N, 103˚29’10"E	Kalasin
135	KSN2-CG-C_36	16˚39’29"N, 103˚29’10"E	Kalasin
136	KSN2-CG-C_37	16˚39’29"N, 103˚29’10"E	Kalasin
137	KSN2-CG-C_38	16˚39’29"N, 103˚29’10"E	Kalasin
138	KSN2-CG-C_39	16˚39’29"N, 103˚29’10"E	Kalasin
139	KSN2-CG-C_40	16˚39’29"N, 103˚29’10"E	Kalasin
140	KSN2-CG-C_41	16˚39’29"N, 103˚29’10"E	Kalasin
141	KSN2-CG-C_42	16˚39’29"N, 103˚29’10"E	Kalasin
142	KSN2-CG-C_43	16˚39’29"N, 103˚29’10"E	Kalasin
143	KSN2-CG-C_44	16˚39’29"N, 103˚29’10"E	Kalasin
144	KSN2-CG-C_45	16˚39’29"N, 103˚29’10"E	Kalasin
145	KSN2-CG-C_46	16˚39’29"N, 103˚29’10"E	Kalasin
146	KSN2-CG-C_47	16˚39’29"N, 103˚29’10"E	Kalasin
147	KSN2-CG-C_48	16˚39’29"N, 103˚29’10"E	Kalasin
148	KSN2-CG-C_49	16˚39’29"N, 103˚29’10"E	Kalasin
149	KSN2-CG-C_50	16˚39’29"N, 103˚29’10"E	Kalasin
150	KSN2-CG-C_51	16˚39’29"N, 103˚29’10"E	Kalasin
151	KSN2-CG-C_52	16˚39’29"N, 103˚29’10"E	Kalasin
152	KSN2-CG-C_53	16˚39’29"N, 103˚29’10"E	Kalasin
153	KSN2-CG-C_54	16˚39’29"N, 103˚29’10"E	Kalasin
154	KSN2-CG-C_55	16˚39’29"N, 103˚29’10"E	Kalasin
155	KSN2-CG-C_56	16˚39’29"N, 103˚29’10"E	Kalasin
156	KSN2-CG-C_57	16˚39’29"N, 103˚29’10"E	Kalasin
157	KSN2-CG-C_58	16˚39’29"N, 103˚29’10"E	Kalasin
158	KSN2-CG-C_59	16˚39’29"N, 103˚29’10"E	Kalasin
159	KSN2-CG-C_60	16˚39’29"N, 103˚29’10"E	Kalasin
160	KSN2-CG-C_61	16˚39’29"N, 103˚29’10"E	Kalasin
161	KSN2-CG-C_62	16˚39’29"N, 103˚29’10"E	Kalasin
162	KSN2-CG-C_63	16˚39’29"N, 103˚29’10"E	Kalasin
163	KSN2-CG-C_64	16˚39’29"N, 103˚29’10"E	Kalasin
164	KSN2-CG-C_65	16˚39’29"N, 103˚29’10"E	Kalasin
165	KSN2-CG-C_66	16˚39’29"N, 103˚29’10"E	Kalasin

No.	Abbreviation/Code	Coordinate	Locality
166	KSN2-CG-C_67	16˚39’29"N, 103˚29’10"E	Kalasin
167	KSN2-CG-C_68	16˚39’29"N, 103˚29’10"E	Kalasin
168	KSN2-CG-C_69	16˚39’29"N, 103˚29’10"E	Kalasin
169	KSN2-CG-C_70	16˚39’29"N, 103˚29’10"E	Kalasin
170	KSN2-CG-C_71	16˚39’29"N, 103˚29’10"E	Kalasin
171	KSN2-CG-C_72	16˚39’29"N, 103˚29’10"E	Kalasin
172	KSN2-CG-C_73	16˚39’29"N, 103˚29’10"E	Kalasin
173	KSN2-CG-C_74	16˚39’29"N, 103˚29’10"E	Kalasin
174	KSN2-CG-C_75	16˚39’29"N, 103˚29’10"E	Kalasin
175	KSN2-CG-C_76	16˚39’29"N, 103˚29’10"E	Kalasin
176	KSN2-CG-C_77	16˚39’29"N, 103˚29’10"E	Kalasin
177	KSN2-CG-C_78	16˚39’29"N, 103˚29’10"E	Kalasin
178	KSN2-CG-C_79	16˚39’29"N, 103˚29’10"E	Kalasin
179	KSN2-CG-C_80	16˚39’29"N, 103˚29’10"E	Kalasin
180	KSN2-CG-C_81	16˚39’29"N, 103˚29’10"E	Kalasin
181	KSN2-CG-C_82	16˚39’29"N, 103˚29’10"E	Kalasin
182	KSN2-CG-C_83	16˚39’29"N, 103˚29’10"E	Kalasin
183	KSN2-CG-C_84	16˚39’29"N, 103˚29’10"E	Kalasin
184	KSN2-CG-C_85	16˚39’29"N, 103˚29’10"E	Kalasin
185	KSN2-CG-C_86	16˚39’29"N, 103˚29’10"E	Kalasin
186	KSN2-CG-C_87	16˚39’29"N, 103˚29’10"E	Kalasin
187	KSN2-CG-C_88	16˚39’29"N, 103˚29’10"E	Kalasin
188	KSN2-CG-C_89	16˚39’29"N, 103˚29’10"E	Kalasin
189	KSN2-CG-C_90	16˚39’29"N, 103˚29’10"E	Kalasin
190	KSN2-CG-C_91	16˚39’29"N, 103˚29’10"E	Kalasin
191	KSN2-CG-C_92	16˚39’29"N, 103˚29’10"E	Kalasin
192	KSN2-CG-C_93	16˚39’29"N, 103˚29’10"E	Kalasin
193	KSN2-CG-C_94	16˚39’29"N, 103˚29’10"E	Kalasin
194	KSN2-CG-C_95	16˚39’29"N, 103˚29’10"E	Kalasin
195	KSN2-CG-C_96	16˚39’29"N, 103˚29’10"E	Kalasin
196	KSN2-CG-C_97	16˚39’29"N, 103˚29’10"E	Kalasin
197	KSN2-CG-C_98	16˚39’29"N, 103˚29’10"E	Kalasin
198	KSN2-CG-C_99	16˚39’29"N, 103˚29’10"E	Kalasin
199	KSN2-CG-C_100	16˚39’29"N, 103˚29’10"E	Kalasin
200	KSN2-CG-C_101	16˚39’29"N, 103˚29’10"E	Kalasin
201	KSN2-CG-C_102	16˚39’29"N, 103˚29’10"E	Kalasin
202	KSN2-CG-C_103	16˚39’29"N, 103˚29’10"E	Kalasin
203	KSN2-CG-C_104	16˚39’29"N, 103˚29’10"E	Kalasin
204	KSN2-CG-C_105	16˚39’29"N, 103˚29’10"E	Kalasin
205	KSN2-CG-C_106	16˚39’29"N, 103˚29’10"E	Kalasin
206	KSN2-CG-C_107	16˚39’29"N, 103˚29’10"E	Kalasin
207	KSN2-CG-C_108	16˚39’29"N, 103˚29’10"E	Kalasin
208	KSN2-CG-C_109	16˚39’29"N, 103˚29’10"E	Kalasin
209	KSN2-CG-C_110	16˚39’29"N, 103˚29’10"E	Kalasin
210	KSN2-CG-C_111	16˚39’29"N, 103˚29’10"E	Kalasin
211	KSN2-CG-C_112	16˚39’29"N, 103˚29’10"E	Kalasin
212	KSN2-CG-C_113	16˚39’29"N, 103˚29’10"E	Kalasin
213	KSN2-CG-C_114	16˚39’29"N, 103˚29’10"E	Kalasin
214	KSN2-CG-C_115	16˚39’29"N, 103˚29’10"E	Kalasin
215	KSN2-CG-C_116	16˚39’29"N, 103˚29’10"E	Kalasin
216	KSN2-CG-C_117	16˚39’29"N, 103˚29’10"E	Kalasin
217	KSN2-CG-C_118	16˚39’29"N, 103˚29’10"E	Kalasin
218	KSN2-CG-C_119	16˚39’29"N, 103˚29’10"E	Kalasin
219	KSN2-CG-C_120	16˚39’29"N, 103˚29’10"E	Kalasin
220	KSN2-CG-C_121	16˚39’29"N, 103˚29’10"E	Kalasin
221	KSN2-CG-C_122	16˚39’29"N, 103˚29’10"E	Kalasin

No.	Abbreviation/Code	Coordinate	Locality
222	KSN2-CG-C_123	16˚39’29"N, 103˚29’10"E	Kalasin
223	KSN2-CG-C_124	16˚39’29"N, 103˚29’10"E	Kalasin
224	KSN2-CG-C_125	16˚39’29"N, 103˚29’10"E	Kalasin
225	KSN2-CG-C_126	16˚39’29"N, 103˚29’10"E	Kalasin
226	KSN2-CG-C_127	16˚39’29"N, 103˚29’10"E	Kalasin
227	KSN2-CG-C_128	16˚39’29"N, 103˚29’10"E	Kalasin
228	KSN2-CG-C_129	16˚39’29"N, 103˚29’10"E	Kalasin
229	KSN2-CG-C_130	16˚39’29"N, 103˚29’10"E	Kalasin
230	KSN2-CG-C_131	16˚39’29"N, 103˚29’10"E	Kalasin
231	KSN2-CG-C_132	16˚39’29"N, 103˚29’10"E	Kalasin
232	KSN2-CG-C_133	16˚39’29"N, 103˚29’10"E	Kalasin
233	KSN2-CG-C_134	16˚39’29"N, 103˚29’10"E	Kalasin
234	UBR-CG-C_1	15°14’25”N 104°51’23”E	Ubon Ratchathani
235	UBR-CG-C_2	15°14’25”N 104°51’23”E	Ubon Ratchathani
236	UBR-CG-C_3	15°14’25”N 104°51’23”E	Ubon Ratchathani
237	UBR-CG-C_4	15°14’25”N 104°51’23”E	Ubon Ratchathani
238	UBR-CG-C_5	15°14’25”N 104°51’23”E	Ubon Ratchathani
239	UBR-CG-C_6	15°14’25”N 104°51’23”E	Ubon Ratchathani
240	SB-CG-C_1	14˚58’13”N, 100˚18’41”E	Sing Buri
241	SB-CG-C_2	14˚58’13”N, 100˚18’41”E	Sing Buri
242	SB-CG-C_3	14˚58’13”N, 100˚18’41”E	Sing Buri
243	SB-CG-C_4	14˚58’13”N, 100˚18’41”E	Sing Buri
244	SB-CG-C_5	14˚58’13”N, 100˚18’41”E	Sing Buri
245	SB-CG-C_6	14˚58’13”N, 100˚18’41”E	Sing Buri
246	SB-CG-C_7	14˚58’13”N, 100˚18’41”E	Sing Buri
247	SB-CM-C_1	14˚58’13”N, 100˚18’41”E	Sing Buri
248	SB-CM-C_2	14˚58’13”N, 100˚18’41”E	Sing Buri
249	SB-CM-C_3	14˚58’13”N, 100˚18’41”E	Sing Buri
250	SB-CM-C_4	14˚58’13”N, 100˚18’41”E	Sing Buri
251	SNK1-CM-W_1	17°21’14”N, 104°19’16”E	Sakon Nakhon
252	SNK1-CM-W_2	17°21’14”N, 104°19’16”E	Sakon Nakhon
253	SNK1-CM-W_3	17°21’14”N, 104°19’16”E	Sakon Nakhon
254	SNK1-CM-W_4	17°21’14”N, 104°19’16”E	Sakon Nakhon
255	SNK1-CM-W_5	17°21’14”N, 104°19’16”E	Sakon Nakhon
256	SNK1-CM-W_6	17°21’14”N, 104°19’16”E	Sakon Nakhon
257	SNK1-CM-W_7	17°21’14”N, 104°19’16”E	Sakon Nakhon
258	SNK1-CM-W_8	17°21’14”N, 104°19’16”E	Sakon Nakhon
259	SNK1-CM-W_9	17°21’14”N, 104°19’16”E	Sakon Nakhon
260	SNK1-CM-W_10	17°21’14”N, 104°19’16”E	Sakon Nakhon
261	SNK1-CM-W_11	17°21’14”N, 104°19’16”E	Sakon Nakhon
262	SNK1-CM-W_12	17°21’14”N, 104°19’16”E	Sakon Nakhon
263	SNK1-CM-W_13	17°21’14”N, 104°19’16”E	Sakon Nakhon
264	SNK1-CM-W_14	17°21’14”N, 104°19’16”E	Sakon Nakhon
265	SNK1-CM-W_15	17°21’14”N, 104°19’16”E	Sakon Nakhon
266	SNK1-CM-W_16	17°21’14”N, 104°19’16”E	Sakon Nakhon
267	SNK1-CM-W_17	17°21’14”N, 104°19’16”E	Sakon Nakhon
268	SNK1-CM-W_18	17°21’14”N, 104°19’16”E	Sakon Nakhon
269	SNK1-CM-W_19	17°21’14”N, 104°19’16”E	Sakon Nakhon
270	SNK1-CM-W_20	17°21’14”N, 104°19’16”E	Sakon Nakhon
271	SNK1-CM-W_21	17°21’14”N, 104°19’16”E	Sakon Nakhon
272	SNK1-CM-W_22	17°21’14”N, 104°19’16”E	Sakon Nakhon
273	SNK1-CM-W_23	17°21’14”N, 104°19’16”E	Sakon Nakhon
274	SNK1-CM-W_24	17°21’14”N, 104°19’16”E	Sakon Nakhon
275	SNK1-CM-W_25	17°21’14”N, 104°19’16”E	Sakon Nakhon
276	SNK1-CM-W_26	17°21’14”N, 104°19’16”E	Sakon Nakhon
277	SNK1-CM-W_27	17°21’14”N, 104°19’16”E	Sakon Nakhon

No.	Abbreviation/Code	Coordinate	Locality
278	SNK1-CM-W_28	17°21’14”N, 104°19’16”E	Sakon Nakhon
279	SNK1-CM-W_29	17°21’14”N, 104°19’16”E	Sakon Nakhon
280	SNK1-CM-W_30	17°21’14”N, 104°19’16”E	Sakon Nakhon
281	SNK1-CM-W_31	17°21’14”N, 104°19’16”E	Sakon Nakhon
282	SNK1-CM-W_32	17°21’14”N, 104°19’16”E	Sakon Nakhon
283	SNK1-CM-W_33	17°21’14”N, 104°19’16”E	Sakon Nakhon
284	SNK1-CM-W_34	17°21’14”N, 104°19’16”E	Sakon Nakhon
285	SNK1-CM-W_35	17°21’14”N, 104°19’16”E	Sakon Nakhon
286	SNK1-CM-W_36	17°21’14”N, 104°19’16”E	Sakon Nakhon
287	SNK1-CM-W_37	17°21’14”N, 104°19’16”E	Sakon Nakhon
288	SNK1-CM-W_38	17°21’14”N, 104°19’16”E	Sakon Nakhon
289	SNK1-CM-W_39	17°21’14”N, 104°19’16”E	Sakon Nakhon
290	SNK1-CM-W_40	17°21’14”N, 104°19’16”E	Sakon Nakhon
291	SNK1-CM-W_41	17°21’14”N, 104°19’16”E	Sakon Nakhon
292	SNK1-CM-W_42	17°21’14”N, 104°19’16”E	Sakon Nakhon
293	SNK1-CM-W_43	17°21’14”N, 104°19’16”E	Sakon Nakhon
294	SNK1-CM-W_44	17°21’14”N, 104°19’16”E	Sakon Nakhon
295	SNK1-CM-W_45	17°21’14”N, 104°19’16”E	Sakon Nakhon
296	SNK1-CM-W_46	17°21’14”N, 104°19’16”E	Sakon Nakhon
297	SNK1-CM-W_47	17°21’14”N, 104°19’16”E	Sakon Nakhon
298	SNK1-CM-W_48	17°21’14”N, 104°19’16”E	Sakon Nakhon
299	SNK1-CM-W_49	17°21’14”N, 104°19’16”E	Sakon Nakhon
300	SNK1-CM-W_50	17°21’14”N, 104°19’16”E	Sakon Nakhon
301	SNK1-CM-W_51	17°21’14”N, 104°19’16”E	Sakon Nakhon
302	SNK1-CM-W_52	17°21’14”N, 104°19’16”E	Sakon Nakhon
303	SNK1-CM-W_53	17°21’14”N, 104°19’16”E	Sakon Nakhon
304	SNK1-CM-W_54	17°21’14”N, 104°19’16”E	Sakon Nakhon
305	SNK1-CM-W_55	17°21’14”N, 104°19’16”E	Sakon Nakhon
306	SNK1-CM-W_56	17°21’14”N, 104°19’16”E	Sakon Nakhon
307	SNK1-CM-W_57	17°21’14”N, 104°19’16”E	Sakon Nakhon
308	SNK1-CM-W_58	17°21’14”N, 104°19’16”E	Sakon Nakhon
309	SNK1-CM-W_59	17°21’14”N, 104°19’16”E	Sakon Nakhon
310	SNK1-CM-W_60	17°21’14”N, 104°19’16”E	Sakon Nakhon
311	SNK1-CM-W_61	17°21’14”N, 104°19’16”E	Sakon Nakhon
312	SNK1-CM-W_62	17°21’14”N, 104°19’16”E	Sakon Nakhon
313	SNK1-CM-W_63	17°21’14”N, 104°19’16”E	Sakon Nakhon
314	SNK1-CM-W_64	17°21’14”N, 104°19’16”E	Sakon Nakhon
315	SNK1-CM-W_65	17°21’14”N, 104°19’16”E	Sakon Nakhon
316	SNK1-CM-W_66	17°21’14”N, 104°19’16”E	Sakon Nakhon
317	SNK1-CM-W_67	17°21’14”N, 104°19’16”E	Sakon Nakhon
318	SNK1-CM-W_68	17°21’14”N, 104°19’16”E	Sakon Nakhon
319	SNK1-CM-W_69	17°21’14”N, 104°19’16”E	Sakon Nakhon
320	SNK1-CM-W_70	17°21’14”N, 104°19’16”E	Sakon Nakhon
321	SNK1-CM-W_71	17°21’14”N, 104°19’16”E	Sakon Nakhon
322	SNK1-CM-W_72	17°21’14”N, 104°19’16”E	Sakon Nakhon
323	SNK1-CM-W_73	17°21’14”N, 104°19’16”E	Sakon Nakhon
324	SNK1-CM-W_74	17°21’14”N, 104°19’16”E	Sakon Nakhon
325	SNK1-CM-W_75	17°21’14”N, 104°19’16”E	Sakon Nakhon
326	SNK1-CM-W_76	17°21’14”N, 104°19’16”E	Sakon Nakhon
327	SNK1-CM-W_77	17°21’14”N, 104°19’16”E	Sakon Nakhon
328	SNK1-CM-W_78	17°21’14”N, 104°19’16”E	Sakon Nakhon
329	SNK1-CM-W_79	17°21’14”N, 104°19’16”E	Sakon Nakhon
330	SNK1-CM-W_80	17°21’14”N, 104°19’16”E	Sakon Nakhon
331	SNK1-CM-W_81	17°21’14”N, 104°19’16”E	Sakon Nakhon
332	SNK1-CM-W_82	17°21’14”N, 104°19’16”E	Sakon Nakhon
333	SNK1-CM-W_83	17°21’14”N, 104°19’16”E	Sakon Nakhon

No.	Abbreviation/Code	Coordinate	Locality
334	SNK1-CM-W_84	17°21’14”N, 104°19’16”E	Sakon Nakhon
335	SNK1-CM-W_85	17°21’14”N, 104°19’16”E	Sakon Nakhon
336	SNK1-CM-W_86	17°21’14”N, 104°19’16”E	Sakon Nakhon
337	SNK1-CM-W_87	17°21’14”N, 104°19’16”E	Sakon Nakhon
338	SNK1-CM-W_88	17°21’14”N, 104°19’16”E	Sakon Nakhon
339	SNK1-CM-W_89	17°21’14”N, 104°19’16”E	Sakon Nakhon
340	SNK1-CM-W_90	17°21’14”N, 104°19’16”E	Sakon Nakhon
341	SNK1-CM-W_91	17°21’14”N, 104°19’16”E	Sakon Nakhon
342	SNK1-CM-W_92	17°21’14”N, 104°19’16”E	Sakon Nakhon
343	SNK1-CM-W_93	17°21’14”N, 104°19’16”E	Sakon Nakhon
344	SNK1-CM-W_94	17°21’14”N, 104°19’16”E	Sakon Nakhon
345	SNK1-CM-W_95	17°21’14”N, 104°19’16”E	Sakon Nakhon
346	SNK1-CM-W_96	17°21’14”N, 104°19’16”E	Sakon Nakhon
347	SNK1-CM-W_97	17°21’14”N, 104°19’16”E	Sakon Nakhon
348	SNK1-CM-W_98	17°21’14”N, 104°19’16”E	Sakon Nakhon
349	SNK1-CM-W_99	17°21’14”N, 104°19’16”E	Sakon Nakhon
350	SNK1-CM-W_100	17°21’14”N, 104°19’16”E	Sakon Nakhon
351	SNK1-CM-W_101	17°21’14”N, 104°19’16”E	Sakon Nakhon
352	SNK1-CM-W_102	17°21’14”N, 104°19’16”E	Sakon Nakhon
353	SNK1-CM-W_103	17°21’14”N, 104°19’16”E	Sakon Nakhon
354	SNK1-CM-W_104	17°21’14”N, 104°19’16”E	Sakon Nakhon
355	SNK1-CM-W_105	17°21’14”N, 104°19’16”E	Sakon Nakhon
356	SNK1-CM-W_106	17°21’14”N, 104°19’16”E	Sakon Nakhon
357	SNK1-CM-W_107	17°21’14”N, 104°19’16”E	Sakon Nakhon
358	SNK1-CM-W_108	17°21’14”N, 104°19’16”E	Sakon Nakhon
359	SNK1-CM-W_109	17°21’14”N, 104°19’16”E	Sakon Nakhon
360	SNK1-CM-W_110	17°21’14”N, 104°19’16”E	Sakon Nakhon
361	SNK1-CM-W_111	17°21’14”N, 104°19’16”E	Sakon Nakhon
362	SNK1-CM-W_112	17°21’14”N, 104°19’16”E	Sakon Nakhon
363	SNK1-CM-W_113	17°21’14”N, 104°19’16”E	Sakon Nakhon
364	SNK1-CM-W_114	17°21’14”N, 104°19’16”E	Sakon Nakhon
365	SNK1-CM-W_115	17°21’14”N, 104°19’16”E	Sakon Nakhon
366	SNK1-CM-W_116	17°21’14”N, 104°19’16”E	Sakon Nakhon
367	SNK1-CM-W_117	17°21’14”N, 104°19’16”E	Sakon Nakhon
368	SNK1-CM-W_118	17°21’14”N, 104°19’16”E	Sakon Nakhon
369	SNK1-CM-W_119	17°21’14”N, 104°19’16”E	Sakon Nakhon
370	SNK1-CM-W_120	17°21’14”N, 104°19’16”E	Sakon Nakhon
371	SNK1-CM-W_121	17°21’14”N, 104°19’16”E	Sakon Nakhon
372	SNK1-CM-W_122	17°21’14”N, 104°19’16”E	Sakon Nakhon
373	SNK1-CM-W_123	17°21’14”N, 104°19’16”E	Sakon Nakhon
374	SNK1-CM-W_124	17°21’14”N, 104°19’16”E	Sakon Nakhon
375	SNK1-CM-W_125	17°21’14”N, 104°19’16”E	Sakon Nakhon
376	SNK1-CM-W_126	17°21’14”N, 104°19’16”E	Sakon Nakhon
377	SNK1-CM-W_127	17°21’14”N, 104°19’16”E	Sakon Nakhon
378	SNK1-CM-W_128	17°21’14”N, 104°19’16”E	Sakon Nakhon
379	SNK1-CM-W_129	17°21’14”N, 104°19’16”E	Sakon Nakhon
380	SNK1-CM-W_130	17°21’14”N, 104°19’16”E	Sakon Nakhon
381	SNK1-CM-W_131	17°21’14”N, 104°19’16”E	Sakon Nakhon
382	SNK1-CM-W_132	17°21’14”N, 104°19’16”E	Sakon Nakhon
383	SNK1-CM-W_133	17°21’14”N, 104°19’16”E	Sakon Nakhon
384	SNK1-CM-W_134	17°21’14”N, 104°19’16”E	Sakon Nakhon
385	SNK1-CM-W_135	17°21’14”N, 104°19’16”E	Sakon Nakhon
386	SNK1-CM-W_136	17°21’14”N, 104°19’16”E	Sakon Nakhon
387	SNK1-CM-W_137	17°21’14”N, 104°19’16”E	Sakon Nakhon
388	SNK1-CM-W_138	17°21’14”N, 104°19’16”E	Sakon Nakhon
389	SNK1-CM-W_139	17°21’14”N, 104°19’16”E	Sakon Nakhon

No.	Abbreviation/Code	Coordinate	Locality
390	SNK1-CM-W_140	17°21’14”N, 104°19’16”E	Sakon Nakhon
391	SNK1-CM-W_141	17°21’14”N, 104°19’16”E	Sakon Nakhon
392	SNK1-CM-W_142	17°21’14”N, 104°19’16”E	Sakon Nakhon
393	SNK1-CM-W_143	17°21’14”N, 104°19’16”E	Sakon Nakhon
394	SNK1-CM-W_144	17°21’14”N, 104°19’16”E	Sakon Nakhon
395	SNK1-CM-W_145	17°21’14”N, 104°19’16”E	Sakon Nakhon
396	SNK1-CM-W_146	17°21’14”N, 104°19’16”E	Sakon Nakhon
397	SNK1-CM-W_147	17°21’14”N, 104°19’16”E	Sakon Nakhon
398	SNK1-CM-W_148	17°21’14”N, 104°19’16”E	Sakon Nakhon
399	SNK1-CM-W_149	17°21’14”N, 104°19’16”E	Sakon Nakhon
400	SNK1-CM-W_150	17°21’14”N, 104°19’16”E	Sakon Nakhon
401	SNK1-CM-W_151	17°21’14”N, 104°19’16”E	Sakon Nakhon
402	SNK1-CM-W_152	17°21’14”N, 104°19’16”E	Sakon Nakhon
403	SNK1-CM-W_153	17°21’14”N, 104°19’16”E	Sakon Nakhon
404	SNK1-CM-W_154	17°21’14”N, 104°19’16”E	Sakon Nakhon
405	SNK1-CM-W_155	17°21’14”N, 104°19’16”E	Sakon Nakhon
406	SNK1-CM-W_156	17°21’14”N, 104°19’16”E	Sakon Nakhon
407	SNK1-CM-W_157	17°21’14”N, 104°19’16”E	Sakon Nakhon
408	SNK1-CM-W_158	17°21’14”N, 104°19’16”E	Sakon Nakhon
409	SNK1-CM-W_159	17°21’14”N, 104°19’16”E	Sakon Nakhon
410	SNK1-CM-W_160	17°21’14”N, 104°19’16”E	Sakon Nakhon
411	SNK1-CM-W_161	17°21’14”N, 104°19’16”E	Sakon Nakhon
412	SNK1-CM-W_162	17°21’14”N, 104°19’16”E	Sakon Nakhon
413	SNK1-CM-W_163	17°21’14”N, 104°19’16”E	Sakon Nakhon
414	SNK1-CM-W_164	17°21’14”N, 104°19’16”E	Sakon Nakhon
415	SNK1-CM-W_165	17°21’14”N, 104°19’16”E	Sakon Nakhon
416	SNK1-CM-W_166	17°21’14”N, 104°19’16”E	Sakon Nakhon
417	SNK1-CM-W_167	17°21’14”N, 104°19’16”E	Sakon Nakhon
418	SNK1-CM-W_168	17°21’14”N, 104°19’16”E	Sakon Nakhon
419	SNK1-CM-W_169	17°21’14”N, 104°19’16”E	Sakon Nakhon
420	SNK1-CM-W_170	17°21’14”N, 104°19’16”E	Sakon Nakhon
421	SNK1-CM-W_171	17°21’14”N, 104°19’16”E	Sakon Nakhon
422	SNK1-CM-W_172	17°21’14”N, 104°19’16”E	Sakon Nakhon
423	SNK1-CM-W_173	17°21’14”N, 104°19’16”E	Sakon Nakhon
424	SNK1-CM-W_174	17°21’14”N, 104°19’16”E	Sakon Nakhon
425	SNK1-CM-W_175	17°21’14”N, 104°19’16”E	Sakon Nakhon
426	SNK1-CM-W_176	17°21’14”N, 104°19’16”E	Sakon Nakhon
427	SNK1-CM-W_177	17°21’14”N, 104°19’16”E	Sakon Nakhon
428	SNK1-CM-W_178	17°21’14”N, 104°19’16”E	Sakon Nakhon
429	SNK1-CM-W_179	17°21’14”N, 104°19’16”E	Sakon Nakhon
430	SNK1-CM-W_180	17°21’14”N, 104°19’16”E	Sakon Nakhon
431	SNK1-CM-W_181	17°21’14”N, 104°19’16”E	Sakon Nakhon
432	SNK1-CM-W_182	17°21’14”N, 104°19’16”E	Sakon Nakhon
433	SNK2-CM-W_1	17°14’05”N, 104°12’28”E	Sakon Nakhon
434	SNK2-CM-W_2	17°14’05”N, 104°12’28”E	Sakon Nakhon
435	SNK2-CM-W_3	17°14’05”N, 104°12’28”E	Sakon Nakhon
436	SNK2-CM-W_4	17°14’05”N, 104°12’28”E	Sakon Nakhon
437	SNK2-CM-W_5	17°14’05”N, 104°12’28”E	Sakon Nakhon
438	SNK2-CM-W_6	17°14’05”N, 104°12’28”E	Sakon Nakhon
439	SNK2-CM-W_7	17°14’05”N, 104°12’28”E	Sakon Nakhon
440	SNK2-CM-W_8	17°14’05”N, 104°12’28”E	Sakon Nakhon
441	SNK2-CM-W_9	17°14’05”N, 104°12’28”E	Sakon Nakhon
442	SNK2-CM-W_10	17°14’05”N, 104°12’28”E	Sakon Nakhon
443	SNK2-CM-W_11	17°14’05”N, 104°12’28”E	Sakon Nakhon
444	SNK2-CM-W_12	17°14’05”N, 104°12’28”E	Sakon Nakhon
445	SNK2-CM-W_13	17°14’05”N, 104°12’28”E	Sakon Nakhon

No.	Abbreviation/Code	Coordinate	Locality
446	SNK2-CM-W_14	17°14’05”N, 104°12’28”E	Sakon Nakhon
447	SNK2-CM-W_15	17°14’05”N, 104°12’28”E	Sakon Nakhon
448	SNK2-CM-W_16	17°14’05”N, 104°12’28”E	Sakon Nakhon
449	SNK2-CM-W_17	17°14’05”N, 104°12’28”E	Sakon Nakhon
450	SNK2-CM-W_18	17°14’05”N, 104°12’28”E	Sakon Nakhon
451	SNK2-CM-W_19	17°14’05”N, 104°12’28”E	Sakon Nakhon
452	SNK2-CM-W_20	17°14’05”N, 104°12’28”E	Sakon Nakhon
453	SNK2-CM-W_21	17°14’05”N, 104°12’28”E	Sakon Nakhon
454	SNK2-CM-W_22	17°14’05”N, 104°12’28”E	Sakon Nakhon
455	SNK2-CM-W_23	17°14’05”N, 104°12’28”E	Sakon Nakhon
456	SNK2-CM-W_24	17°14’05”N, 104°12’28”E	Sakon Nakhon
457	SNK2-CM-W_25	17°14’05”N, 104°12’28”E	Sakon Nakhon
458	SNK2-CM-W_26	17°14’05”N, 104°12’28”E	Sakon Nakhon
459	SNK2-CM-W_27	17°14’05”N, 104°12’28”E	Sakon Nakhon
460	SNK2-CM-W_28	17°14’05”N, 104°12’28”E	Sakon Nakhon
461	SNK2-CM-W_29	17°14’05”N, 104°12’28”E	Sakon Nakhon
462	SNK2-CM-W_30	17°14’05”N, 104°12’28”E	Sakon Nakhon
463	SNK2-CM-W_31	17°14’05”N, 104°12’28”E	Sakon Nakhon
464	SNK2-CM-W_32	17°14’05”N, 104°12’28”E	Sakon Nakhon
465	SNK2-CM-W_33	17°14’05”N, 104°12’28”E	Sakon Nakhon
466	SNK2-CM-W_34	17°14’05”N, 104°12’28”E	Sakon Nakhon
467	SNK2-CM-W_35	17°14’05”N, 104°12’28”E	Sakon Nakhon
468	SNK2-CM-W_36	17°14’05”N, 104°12’28”E	Sakon Nakhon
469	SNK2-CM-W_37	17°14’05”N, 104°12’28”E	Sakon Nakhon
470	SNK2-CM-W_38	17°14’05”N, 104°12’28”E	Sakon Nakhon
471	SNK2-CM-W_39	17°14’05”N, 104°12’28”E	Sakon Nakhon
472	SNK2-CM-W_40	17°14’05”N, 104°12’28”E	Sakon Nakhon
473	SNK2-CM-W_41	17°14’05”N, 104°12’28”E	Sakon Nakhon
474	SNK2-CM-W_42	17°14’05”N, 104°12’28”E	Sakon Nakhon
475	SNK2-CM-W_43	17°14’05”N, 104°12’28”E	Sakon Nakhon
476	SNK2-CM-W_44	17°14’05”N, 104°12’28”E	Sakon Nakhon
477	SNK2-CM-W_45	17°14’05”N, 104°12’28”E	Sakon Nakhon
478	SNK2-CM-W_46	17°14’05”N, 104°12’28”E	Sakon Nakhon
479	SNK2-CM-W_47	17°14’05”N, 104°12’28”E	Sakon Nakhon
480	SNK2-CM-W_48	17°14’05”N, 104°12’28”E	Sakon Nakhon
481	SNK2-CM-W_49	17°14’05”N, 104°12’28”E	Sakon Nakhon
482	SNK2-CM-W_50	17°14’05”N, 104°12’28”E	Sakon Nakhon
483	SNK2-CM-W_51	17°14’05”N, 104°12’28”E	Sakon Nakhon
484	SNK2-CM-W_52	17°14’05”N, 104°12’28”E	Sakon Nakhon
485	SNK2-CM-W_53	17°14’05”N, 104°12’28”E	Sakon Nakhon
486	SNK2-CM-W_54	17°14’05”N, 104°12’28”E	Sakon Nakhon
487	SNK2-CM-W_55	17°14’05”N, 104°12’28”E	Sakon Nakhon
488	SNK2-CM-W_56	17°14’05”N, 104°12’28”E	Sakon Nakhon
489	SNK2-CM-W_57	17°14’05”N, 104°12’28”E	Sakon Nakhon
490	SNK2-CM-W_58	17°14’05”N, 104°12’28”E	Sakon Nakhon
491	SNK2-CM-W_59	17°14’05”N, 104°12’28”E	Sakon Nakhon
492	SNK2-CM-W_60	17°14’05”N, 104°12’28”E	Sakon Nakhon
493	SNK2-CM-W_61	17°14’05”N, 104°12’28”E	Sakon Nakhon
494	SNK2-CM-W_62	17°14’05”N, 104°12’28”E	Sakon Nakhon
495	SNK2-CM-W_63	17°14’05”N, 104°12’28”E	Sakon Nakhon
496	SNK2-CM-W_64	17°14’05”N, 104°12’28”E	Sakon Nakhon
497	SNK2-CM-W_65	17°14’05”N, 104°12’28”E	Sakon Nakhon
498	SNK2-CM-W_66	17°14’05”N, 104°12’28”E	Sakon Nakhon
499	SNK2-CM-W_67	17°14’05”N, 104°12’28”E	Sakon Nakhon
500	SNK2-CM-W_68	17°14’05”N, 104°12’28”E	Sakon Nakhon
501	SNK2-CM-W_69	17°14’05”N, 104°12’28”E	Sakon Nakhon

No.	Abbreviation/Code	Coordinate	Locality
502	SNK2-CM-W_70	17°14’05”N, 104°12’28”E	Sakon Nakhon
503	SNK2-CM-W_71	17°14’05”N, 104°12’28”E	Sakon Nakhon
504	SNK2-CM-W_72	17°14’05”N, 104°12’28”E	Sakon Nakhon
505	SNK2-CM-W_73	17°14’05”N, 104°12’28”E	Sakon Nakhon
506	SNK2-CM-W_74	17°14’05”N, 104°12’28”E	Sakon Nakhon
507	SKN3-CM-W_1	17°14’46”N, 104°23’28”E	Sakon Nakhon
508	SKN3-CM-W_2	17°14’46”N, 104°23’28”E	Sakon Nakhon
509	SKN3-CM-W_3	17°14’46”N, 104°23’28”E	Sakon Nakhon
510	SKN3-CM-W_4	17°14’46”N, 104°23’28”E	Sakon Nakhon
511	SKN3-CM-W_5	17°14’46”N, 104°23’28”E	Sakon Nakhon
512	SKN3-CM-W_6	17°14’46”N, 104°23’28”E	Sakon Nakhon
513	SKN3-CM-W_7	17°14’46”N, 104°23’28”E	Sakon Nakhon
514	SKN3-CM-W_8	17°14’46”N, 104°23’28”E	Sakon Nakhon
515	SKN3-CM-W_9	17°14’46”N, 104°23’28”E	Sakon Nakhon
516	SKN3-CM-W_10	17°14’46”N, 104°23’28”E	Sakon Nakhon
517	SKN3-CM-W_11	17°14’46”N, 104°23’28”E	Sakon Nakhon
518	SKN3-CM-W_12	17°14’46”N, 104°23’28”E	Sakon Nakhon
519	SKN3-CM-W_13	17°14’46”N, 104°23’28”E	Sakon Nakhon
520	SKN3-CM-W_14	17°14’46”N, 104°23’28”E	Sakon Nakhon
521	SKN3-CM-W_15	17°14’46”N, 104°23’28”E	Sakon Nakhon
522	SKN3-CM-W_16	17°14’46”N, 104°23’28”E	Sakon Nakhon
523	SKN3-CM-W_17	17°14’46”N, 104°23’28”E	Sakon Nakhon
524	SKN3-CM-W_18	17°14’46”N, 104°23’28”E	Sakon Nakhon
525	SKN3-CM-W_19	17°14’46”N, 104°23’28”E	Sakon Nakhon
526	SKN3-CM-W_20	17°14’46”N, 104°23’28”E	Sakon Nakhon
527	SKN3-CM-W_21	17°14’46”N, 104°23’28”E	Sakon Nakhon
528	SKN3-CM-W_22	17°14’46”N, 104°23’28”E	Sakon Nakhon
529	SKN3-CM-W_23	17°14’46”N, 104°23’28”E	Sakon Nakhon
530	SKN3-CM-W_24	17°14’46”N, 104°23’28”E	Sakon Nakhon
531	SKN3-CM-W_25	17°14’46”N, 104°23’28”E	Sakon Nakhon
532	SKN3-CM-W_26	17°14’46”N, 104°23’28”E	Sakon Nakhon
533	SKN3-CM-W_27	17°14’46”N, 104°23’28”E	Sakon Nakhon
534	SKN3-CM-W_28	17°14’46”N, 104°23’28”E	Sakon Nakhon
535	SKN3-CM-W_29	17°14’46”N, 104°23’28”E	Sakon Nakhon
536	SKN3-CM-W_30	17°14’46”N, 104°23’28”E	Sakon Nakhon
537	SKN3-CM-W_31	17°14’46”N, 104°23’28”E	Sakon Nakhon
538	SKN3-CM-W_32	17°14’46”N, 104°23’28”E	Sakon Nakhon
539	SKN3-CM-W_33	17°14’46”N, 104°23’28”E	Sakon Nakhon
540	SKN3-CM-W_34	17°14’46”N, 104°23’28”E	Sakon Nakhon
541	SKN3-CM-W_35	17°14’46”N, 104°23’28”E	Sakon Nakhon
542	SKN3-CM-W_36	17°14’46”N, 104°23’28”E	Sakon Nakhon
543	SKN3-CM-W_37	17°14’46”N, 104°23’28”E	Sakon Nakhon
544	SKN3-CM-W_38	17°14’46”N, 104°23’28”E	Sakon Nakhon
545	SKN3-CM-W_39	17°14’46”N, 104°23’28”E	Sakon Nakhon
546	SKN3-CM-W_40	17°14’46”N, 104°23’28”E	Sakon Nakhon
547	SKN3-CM-W_41	17°14’46”N, 104°23’28”E	Sakon Nakhon
548	SKN3-CM-W_42	17°14’46”N, 104°23’28”E	Sakon Nakhon
549	SKN3-CM-W_43	17°14’46”N, 104°23’28”E	Sakon Nakhon
550	SKN3-CM-W_44	17°14’46”N, 104°23’28”E	Sakon Nakhon
551	SKN3-CM-W_45	17°14’46”N, 104°23’28”E	Sakon Nakhon
552	SKN3-CM-W_46	17°14’46”N, 104°23’28”E	Sakon Nakhon
553	SKN3-CM-W_47	17°14’46”N, 104°23’28”E	Sakon Nakhon
554	SKN3-CM-W_48	17°14’46”N, 104°23’28”E	Sakon Nakhon
555	SKN3-CM-W_49	17°14’46”N, 104°23’28”E	Sakon Nakhon
556	SKN3-CM-W_50	17°14’46”N, 104°23’28”E	Sakon Nakhon
557	SKN3-CM-W_51	17°14’46”N, 104°23’28”E	Sakon Nakhon

No.	Abbreviation/Code	Coordinate	Locality
558	SKN3-CM-W_52	17°14’46”N, 104°23’28”E	Sakon Nakhon
559	SKN3-CM-W_53	17°14’46”N, 104°23’28”E	Sakon Nakhon
560	SKN3-CM-W_54	17°14’46”N, 104°23’28”E	Sakon Nakhon
561	SKN3-CM-W_55	17°14’46”N, 104°23’28”E	Sakon Nakhon
562	SKN3-CM-W_56	17°14’46”N, 104°23’28”E	Sakon Nakhon
563	SKN3-CM-W_57	17°14’46”N, 104°23’28”E	Sakon Nakhon
564	SKN3-CM-W_58	17°14’46”N, 104°23’28”E	Sakon Nakhon
565	SKN3-CM-W_59	17°14’46”N, 104°23’28”E	Sakon Nakhon
566	SKN3-CM-W_60	17°14’46”N, 104°23’28”E	Sakon Nakhon
567	SKN3-CM-W_61	17°14’46”N, 104°23’28”E	Sakon Nakhon
568	SKN3-CM-W_62	17°14’46”N, 104°23’28”E	Sakon Nakhon
569	SKN3-CM-W_63	17°14’46”N, 104°23’28”E	Sakon Nakhon
570	SKN3-CM-W_64	17°14’46”N, 104°23’28”E	Sakon Nakhon
571	SKN3-CM-W_65	17°14’46”N, 104°23’28”E	Sakon Nakhon
572	SKN3-CM-W_66	17°14’46”N, 104°23’28”E	Sakon Nakhon
573	SKN3-CM-W_67	17°14’46”N, 104°23’28”E	Sakon Nakhon
574	SKN3-CM-W_68	17°14’46”N, 104°23’28”E	Sakon Nakhon
575	SKN3-CM-W_69	17°14’46”N, 104°23’28”E	Sakon Nakhon
576	SKN3-CM-W_70	17°14’46”N, 104°23’28”E	Sakon Nakhon
577	SKN3-CM-W_71	17°14’46”N, 104°23’28”E	Sakon Nakhon
578	SKN3-CM-W_72	17°14’46”N, 104°23’28”E	Sakon Nakhon
579	SKN3-CM-W_73	17°14’46”N, 104°23’28”E	Sakon Nakhon
580	SKN3-CM-W_74	17°14’46”N, 104°23’28”E	Sakon Nakhon
581	SKN3-CM-W_75	17°14’46”N, 104°23’28”E	Sakon Nakhon
582	SKN3-CM-W_76	17°14’46”N, 104°23’28”E	Sakon Nakhon
583	SKN3-CM-W_77	17°14’46”N, 104°23’28”E	Sakon Nakhon
584	SKN3-CM-W_78	17°14’46”N, 104°23’28”E	Sakon Nakhon
585	SKN3-CM-W_79	17°14’46”N, 104°23’28”E	Sakon Nakhon
586	SKN3-CM-W_80	17°14’46”N, 104°23’28”E	Sakon Nakhon
587	SKN3-CM-W_81	17°14’46”N, 104°23’28”E	Sakon Nakhon
588	SKN3-CM-W_82	17°14’46”N, 104°23’28”E	Sakon Nakhon
589	SNK4-CM-C_1	17°17’06”N, 104°15’24”E	Sakon Nakhon
590	SNK4-CM-C_2	17°17’06”N, 104°15’24”E	Sakon Nakhon
591	SNK4-CM-C_3	17°17’06”N, 104°15’24”E	Sakon Nakhon
592	SNK4-CM-C_4	17°17’06”N, 104°15’24”E	Sakon Nakhon
593	SNK4-CM-C_5	17°17’06”N, 104°15’24”E	Sakon Nakhon
594	SNK4-CM-C_6	17°17’06”N, 104°15’24”E	Sakon Nakhon
595	SNK4-CM-C_7	17°17’06”N, 104°15’24”E	Sakon Nakhon
596	SNK4-CM-C_8	17°17’06”N, 104°15’24”E	Sakon Nakhon
597	SNK4-CM-C_9	17°17’06”N, 104°15’24”E	Sakon Nakhon
598	SNK4-CM-C_10	17°17’06”N, 104°15’24”E	Sakon Nakhon
599	SNK4-CM-C_11	17°17’06”N, 104°15’24”E	Sakon Nakhon
600	SNK4-CM-C_12	17°17’06”N, 104°15’24”E	Sakon Nakhon
601	SNK4-CM-C_13	17°17’06”N, 104°15’24”E	Sakon Nakhon
602	SNK4-CM-C_14	17°17’06”N, 104°15’24”E	Sakon Nakhon
603	SPB1-CM-W_1	14°14’57.1”N, 100°11’13.0”E	Suphan Buri
604	SPB1-CM-W_2	14°14’57.1”N, 100°11’13.0”E	Suphan Buri
605	SPB1-CM-W_3	14°14’57.1”N, 100°11’13.0”E	Suphan Buri
606	SPB1-CM-W_4	14°14’57.1”N, 100°11’13.0”E	Suphan Buri
607	SPB1-CM-W_5	14°14’57.1”N, 100°11’13.0”E	Suphan Buri
608	SPB1-CM-W_6	14°14’57.1”N, 100°11’13.0”E	Suphan Buri
609	SPB2-CM-W_1	14°14’40.5”N, 100°07’60.0”E	Suphan Buri
610	SPB2-CM-W_2	14°14’40.5”N, 100°07’60.0”E	Suphan Buri
611	SPB2-CM-W_3	14°14’40.5”N, 100°07’60.0”E	Suphan Buri
612	NPT1-CM-W_1	13°59’17.7”N, 99°56’37.3”E	Nakhon Pathom
613	NPT1-CM-W_2	13°59’17.7”N, 99°56’37.3”E	Nakhon Pathom

No.	Abbreviation/Code	Coordinate	Locality
614	NPT2-CM-W_1	13°58'01"N, 100°24'17"E	Nakhon Pathom
615	NPT2-CM-W_2	13°58'01"N, 100°24'17"E	Nakhon Pathom
616	NST1-CM-W_1	7°58'12"N, 99°58'12"E	Nakhon Si Thammarat
617	NST1-CM-W_2	7°58'12"N, 99°58'12"E	Nakhon Si Thammarat
618	NST1-CM-W_3	7°58'12"N, 99°58'12"E	Nakhon Si Thammarat
619	NST2-CM-C_1	8°60'27"N, 100°17'03"E	Nakhon Si Thammarat
620	NST2-CM-C_2	8°60'27"N, 100°17'03"E	Nakhon Si Thammarat
621	NST2-CM-C_3	8°60'27"N, 100°17'03"E	Nakhon Si Thammarat
622	NST2-CM-C_4	8°60'27"N, 100°17'03"E	Nakhon Si Thammarat
623	NST2-CM-C_5	8°60'27"N, 100°17'03"E	Nakhon Si Thammarat
624	NST2-CM-C_6	8°60'27"N, 100°17'03"E	Nakhon Si Thammarat
625	NST2-CM-C_7	8°60'27"N, 100°17'03"E	Nakhon Si Thammarat
626	NST2-CM-C_8	8°60'27"N, 100°17'03"E	Nakhon Si Thammarat
627	NST2-CM-C_9	8°60'27"N, 100°17'03"E	Nakhon Si Thammarat
628	NST2-CM-C_10	8°60'27"N, 100°17'03"E	Nakhon Si Thammarat
629	STN-CM-C_1	9°15'52"N, 99°42'84"E	Surat Thani
630	STN-CM-C_2	9°15'52"N, 99°42'84"E	Surat Thani
631	STN-CM-C_3	9°15'52"N, 99°42'84"E	Surat Thani
632	STN-CM-C_4	9°15'52"N, 99°42'84"E	Surat Thani
633	STN-CM-C_5	9°15'52"N, 99°42'84"E	Surat Thani
634	STN-CM-C_6	9°15'52"N, 99°42'84"E	Surat Thani
635	STN-CM-C_7	9°15'52"N, 99°42'84"E	Surat Thani
636	STN-CM-C_8	9°15'52"N, 99°42'84"E	Surat Thani
637	STN-CM-C_9	9°15'52"N, 99°42'84"E	Surat Thani
638	STN-CM-C_10	9°15'52"N, 99°42'84"E	Surat Thani
639	STN-CM-C_11	9°15'52"N, 99°42'84"E	Surat Thani
640	STN-CM-C_12	9°15'52"N, 99°42'84"E	Surat Thani
641	STN-CM-C_13	9°15'52"N, 99°42'84"E	Surat Thani
642	STN-CM-C_14	9°15'52"N, 99°42'84"E	Surat Thani
643	STN-CM-C_15	9°15'52"N, 99°42'84"E	Surat Thani
644	STN-CM-C_16	9°15'52"N, 99°42'84"E	Surat Thani
645	STN-CM-C_17	9°15'52"N, 99°42'84"E	Surat Thani
646	STN-CM-C_18	9°15'52"N, 99°42'84"E	Surat Thani
647	STN-CM-C_19	9°15'52"N, 99°42'84"E	Surat Thani
648	STN-CM-C_20	9°15'52"N, 99°42'84"E	Surat Thani
649	STN-CM-C_21	9°15'52"N, 99°42'84"E	Surat Thani
650	STN-CM-C_22	9°15'52"N, 99°42'84"E	Surat Thani
651	STN-CM-C_23	9°15'52"N, 99°42'84"E	Surat Thani
652	STN-CM-C_24	9°15'52"N, 99°42'84"E	Surat Thani
653	STN-CM-C_25	9°15'52"N, 99°42'84"E	Surat Thani
654	NKPN-CM-C_1	17°37'50"N 104°14'56"E	Nakhon Phanom
655	NKPN-CM-C_2	17°37'50"N 104°14'56"E	Nakhon Phanom
656	NKPN-CM-C_3	17°37'50"N 104°14'56"E	Nakhon Phanom
657	NKPN-CM-C_4	17°37'50"N 104°14'56"E	Nakhon Phanom
658	NKPN-CM-C_5	17°37'50"N 104°14'56"E	Nakhon Phanom
659	NKPN-CM-C_6	17°37'50"N 104°14'56"E	Nakhon Phanom
660	NKPN-CM-C_7	17°37'50"N 104°14'56"E	Nakhon Phanom
661	UBR-CM-C_1	15°14'25"N 104°51'23"E	Ubon Ratchathani
662	UBR-CM-C_2	15°14'25"N 104°51'23"E	Ubon Ratchathani
663	UBR-CM-C_3	15°14'25"N 104°51'23"E	Ubon Ratchathani
664	UBR-CM-C_4	15°14'25"N 104°51'23"E	Ubon Ratchathani
665	UBR-CM-C_5	15°14'25"N 104°51'23"E	Ubon Ratchathani
666	UBR-CM-C_6	15°14'25"N 104°51'23"E	Ubon Ratchathani
667	UBR-CB-C_1	15°14'25"N 104°51'23"E	Ubon Ratchathani
668	UBR-CB-C_2	15°14'25"N 104°51'23"E	Ubon Ratchathani
669	UBR-CB-C_3	15°14'25"N 104°51'23"E	Ubon Ratchathani

No.	Abbreviation/Code	Coordinate	Locality
670	UBR-CB-C_4	15°14'25"N 104°51'23"E	Ubon Ratchathani
671	UBR-CB-C_5	15°14'25"N 104°51'23"E	Ubon Ratchathani
672	UBR-CB-C_6	15°14'25"N 104°51'23"E	Ubon Ratchathani
673	UBR-CB-C_7	15°14'25"N 104°51'23"E	Ubon Ratchathani
674	UBR-CB-C_8	15°14'25"N 104°51'23"E	Ubon Ratchathani

NYK, Nakhon Nayok; KSN, Kalasin; UBR, Ubon Ratchathani; SB, Sing Buri; SNK, Sakon Nakhon; SPB, Suphan Buri; NPT, Nakhon Pathom; NST, Nakhon Si Thammarat; STN, Surat Thani; NKPN; Nakhon Phanom; CG, *Clarias gariephinus*; CM, *Clarias macrocephalus*; CB, *Clarias batrachus*; C, captive; W, wild

Table S2. The catfish specimens used in this study.

Species	Population	Code	*N**	Location	District	Province	GPS coordinate
C. gariephinus	Nakhon Nayok	NYK-CG-C	5	Jeed Jad Farm	Mueang Nakhon Nayok	Nakhon Nayok	14˚05’58”N, 101˚09’39”E
	Kalasin 1	KSN1-CG-C	94	Kalasin Fish Farm (Betagro), Phu Sing	Mueang Kalasin	Kalasin	16°29'45.6"N 103°25'8.9"E
	Kalasin 2	KSN2-CG-C	134	Kalasin Fish Farm, Phusing	Mueang Kalasin	Kalasin	16˚39’29"N, 103˚29’10"E
	Ubon Ratchathani	UBR-CG-C	6	Mun river	Mueang Ubon Ratchathani	Ubon Ratchathani	15°14’25”N 104°51’23”E
	Sing Buri	SB-CG-C	7	Phakin fish species	In Buri	Sing Buri	14°58’13”N, 100˚18’41”E
C. macrocephalus	Sing Buri	SB-CM-C	4	Phakin fish species	In Buri	Sing Buri	14°58’13”N, 100˚18’41”E
	Sakon Nakhon 1	SNK1-CM-W	182	Nong Han	Mueang Sakon Nakhon	Sakon Nakhon	17°21’14”N, 104°19’16”E
	Sakon Nakhon 2	SNK2-CM-W	74	Susarn Donkajo	Mueang Sakon Nakhon	Sakon Nakhon	17°14’05”N, 104°12’28”E
	Sakon Nakhon 3	SNK3-CM-W	82	Barnthavat	Mueang Sakon Nakhon	Sakon Nakhon	17°14’46”N, 104°23’28”E
	Sakon Nakhon 4	SNK4-CM-C	14	Fisheries Research and Development center Sakon Nakhon	Mueang Sakon Nakhon	Sakon Nakhon	17°17’06”N, 104°15’24”E
	Suphan Buri 1	SPB1-CM-W	6	Ban Chang Subdistrict	Song Phi Nong	Suphan Buri	14°14’57.1”N, 100°11’13.0”E
	Suphan Buri 2	SPB2-CM-W	3	Khlong Bang Khan Chang, Bang Ta then subdistrict	Song Phi Nong	Suphan Buri	14°14’40.5”N, 100°07’60.0”E
	Nakhon Pathom 1	NPT1-CM-W	2	Khlong Mon Thong	Kamphaeng Saen	Nakhon Pathom	13°59’17.7”N, 99°56’37.3”E
	Nakhon Pathom 2	NPT2-CM-W	2	Tha Chin Rever Basin	Nakhon Chai Si	Nakhon Pathom	13°58’01”N, 100°24’17”E
	Nakhon Si Thammarat 1	NST1-CM-W	3	River	Cha-uat	Nakhon Si Thammarat	7°58’12”N, 99°58’12”E

Species	Population	Code	*N**	Location	District	Province	GPS coordinate
	Nakhon Si Thammarat 2	NST2-CM-C	10	Nakhon Si Thammarat Pond	Phrom Khiri	Nakhon Si Thammarat	8°60’27”N, 100°17’03”E
	Surat Thani	STN-CM-C	25	Fisheries Center Surat Thani	Kanchanadit	Surat Thani	9°15’52”N, 99°42’84”E
	Nakhon Phanom	NKPN-CM-C	7	Songkhram river	Si Songkhram	Nakhon Phanom	17°37’50”N 104°14’56”E
	Ubon Ratchathani	UBR-CM-C	6	Mun river	Mueang Ubon Ratchathani	Ubon Ratchathani	15°14’25”N 104°51’23”E
C. batrachus	Ubon Ratchathani	UBR-CB-C	8	Mun river	Mueang Ubon Ratchathani	Ubon Ratchathani	15°14’25”N 104°51’23”E

**N*, number of samples; NYK, Nakhon Nayok; KSN, Kalasin; UBR, Ubon Ratchathani; SB, Sing Buri; SNK, Sakon Nakhon; SPB, Suphan Buri; NPT, Nakhon Pathom; NST, Nakhon Si Thammarat; STN, Surat Thani; NKPN; Nakhon Phanom; CG, *Clarias gariephinus*; CM, *Clarias macrocephalus*; CB, *Clarias batrachus*; C, captive; W, wild

Table S3. Variable sites in the sequences of *MHC I* alleles of North African and bighead catfish populations in Thailand.

Allele	Variable nucleotide position (Accession number: XM53477511)																										
	**816**	**817**	**820**	**821**	**822**	**823**	**824**	**825**	**827**	**828**	**829**	**830**	**834**	**835**	**836**	**838**	**839**	**840**	**845**	**848**	**853**	**863**	**866**	**871**	**872**	**874**	**875**	**876**
**XM53477511**	**C**	**G**	**G**	**T**	**T**	**C**	**T**	**A**	**C**	**C**	**T**	**A**	**A**	**T**	**G**	**G**	**G**	**A**	**A**	**G**	**G**	**A**	**T**	**T**	**C**	**G**	**G**	**T**
*Clarias_MHCI*TH1*	T	.	.	C	.	G	G	.	A	T	.	G	G	G	A	A	A	T	.	.	T	G	C	C	A	.	.	.
*Clarias_MHCI*TH2*	T	.	.	C	.	G	G	.	A	T	.	G	G	G	A	A	A	T	.	.	T	G	C	C	A	.	.	.
*Clarias_MHCI*TH3*	T	.	.	A	.	A	G	.	A	T	.	G	G	.	.	.	A	T	.	.	T	-	-	C	A	.	.	.
*Clarias_MHCI*TH4*	T	.	.	.	.	G	G	.	A	T	.	G	G	.	.	T	A	T	.	.	T	G	C	C	A	.	T	.
*Clarias_MHCI*TH5*	T	.	.	G	.	G	G	.	A	T	.	G	G	.	.	.	A	T	.	.	T	.	C	C	A	.	.	.
*Clarias_MHCI*TH6*	T	.	.	.	.	G	G	.	A	T	.	G	G	.	.	.	A	T	.	.	T	.	C	C	A	.	.	.
*Clarias_MHCI*TH7*	T	.	.	.	.	G	G	.	A	T	.	G	G	.	.	.	A	T	.	.	T	.	C	C	A	.	.	.
*Clarias_MHCI*TH8*	T	.	.	.	.	G	G	.	A	T	.	G	G	.	.	T	A	T	.	.	T	G	C	C	A	.	T	.
*Clarias_MHCI*TH9*	T	.	.	.	.	G	G	.	A	T	.	G	G	.	.	.	A	T	.	.	T	.	C	C	A	.	.	.
*Clarias_MHCI*TH10*	T	.	.	A	.	A	G	.	A	T	.	G	G	.	.	.	A	T	.	.	T	G	C	C	A	.	.	.
*Clarias_MHCI*TH11*	T	.	.	C	.	G	G	.	A	T	.	G	G	G	A	A	A	T	.	.	T	G	C	C	A	.	.	.
*Clarias_MHCI*TH12*	T	.	.	C	.	G	G	.	A	T	.	G	G	G	.	.	A	T	.	.	T	G	C	C	A	.	.	.
*Clarias_MHCI*TH13*	T	.	.	C	.	G	G	.	A	T	.	G	G	G	A	A	A	T	.	.	T	G	C	C	A	.	.	.
*Clarias_MHCI*TH14*	T	.	.	C	.	G	G	.	A	T	.	G	G	C	.	.	A	T	.	.	T	G	C	C	A	.	.	.
*Clarias_MHCI*TH15*	T	.	.	C	.	G	G	.	A	T	.	G	G	G	A	A	A	T	.	.	T	G	C	C	A	.	.	.
*Clarias_MHCI*TH16*	T	.	.	G	.	G	G	.	A	T	.	G	G	G	A	A	A	T	.	.	T	G	C	C	A	.	.	.
*Clarias_MHCI*TH17*	T	A	.	C	.	G	G	.	A	T	.	G	G	.	.	.	A	T	.	.	T	G	C	C	A	.	.	.
*Clarias_MHCI*TH18*	T	.	.	C	.	G	G	.	A	T	.	.	G	G	.	.	A	T	.	.	T	G	C	C	A	.	.	.
*Clarias_MHCI*TH19*	T	.	.	C	.	G	G	.	A	T	.	.	G	G	.	.	A	T	.	.	T	G	C	C	A	.	.	.
*Clarias_MHCI*TH20*	T	.	.	C	.	G	A	.	A	T	.	T	G	.	.	.	A	T	.	.	T	G	C	C	A	.	.	.
*Clarias_MHCI*TH21*	.	C	.	C	.	G	G	.	A	T	.	G	G	G	.	.	A	T	.	.	T	G	C	C	A	.	.	.
*Clarias_MHCI*TH22*	T	.	.	G	.	G	G	.	A	T	.	G	G	G	A	A	A	T	.	.	T	G	C	C	A	.	.	.
*Clarias_MHCI*TH23*	.	C	.	.	.	G	G	.	A	T	.	G	G	G	.	.	A	T	.	.	T	G	C	C	A	.	.	.
*Clarias_MHCI*TH24*	.	C	.	C	.	G	G	.	A	T	.	G	G	G	.	.	A	T	.	.	T	G	C	C	A	.	.	.
*Clarias_MHCI*TH25*	T	.	.	C	.	G	G	.	A	T	.	G	G	.	T	.	A	T	.	.	T	G	A	C	A	.	.	.
*Clarias_MHCI*TH26*	T	.	.	C	.	G	G	.	A	T	.	.	G	G	.	.	A	T	.	.	A	G	C	C	A	.	.	.
*Clarias_MHCI*TH27*	T	A	.	C	.	G	G	.	A	T	.	G	G	.	.	.	A	T	.	.	T	G	C	C	A	.	A	.
*Clarias_MHCI*TH28*	T	A	.	C	.	A	G	.	A	T	.	G	G	G	.	.	A	T	.	.	T	G	C	C	A	.	.	.
*Clarias_MHCI*TH29*	.	C	.	C	.	G	G	.	A	T	.	.	G	G	.	.	A	T	.	.	T	G	C	C	A	.	.	.
*Clarias_MHCI*TH30*	T	.	.	C	.	G	G	.	A	T	.	G	G	G	.	.	A	T	.	.	T	G	C	.	A	.	.	.

Allele	Variable nucleotide position (Accession number: XM53477511)																											
	**816**	**817**	**820**	**821**	**822**	**823**	**824**	**825**	**827**	**828**	**829**	**830**	**834**	**835**	**836**	**838**	**839**	**840**	**845**	**848**	**853**	**863**	**866**	**871**	**872**	**874**	**875**	**876**
*Clarias_MHCI*TH31*	.	C	.	C	.	G	G	.	A	T	.	G	G	G	.	.	A	T	.	.	T	G	C	C	A	.	.	.
*Clarias_MHCI*TH32*	T	.	.	C	.	G	A	.	A	T	.	.	G	.	.	.	A	T	.	.	T	G	C	C	A	.	.	.
*Clarias_MHCI*TH33*	.	C	.	.	.	G	G	.	A	T	.	G	G	G	.	.	A	T	.	.	T	G	C	C	A	.	.	.
*Clarias_MHCI*TH34*	T	.	.	C	.	G	G	.	A	T	.	.	G	G	.	.	A	T	.	.	T	G	C	C	A	.	.	.
*Clarias_MHCI*TH35*	T	.	.	C	.	G	A	.	A	T	.	G	G	.	.	.	A	T	.	.	T	G	C	C	A	.	.	.
*Clarias_MHCI*TH36*	T	.	.	C	.	G	G	.	A	T	.	G	G	G	.	.	A	T	.	.	T	G	C	C	A	.	.	.
*Clarias_MHCI*TH37*	T	.	.	C	.	G	G	.	A	T	.	G	G	G	A	A	A	T	.	.	T	G	C	C	A	.	.	.
*Clarias_MHCI*TH38*	T	.	.	C	.	G	G	.	A	T	.	G	G	.	T	.	A	T	.	.	T	G	A	C	A	.	.	.
*Clarias_MHCI*TH39*	T	.	.	C	.	G	G	.	T	T	.	G	G	G	A	A	A	T	.	.	T	G	C	C	A	.	.	.
*Clarias_MHCI*TH40*	.	C	.	C	.	G	G	.	A	T	.	G	G	G	.	.	A	T	.	.	T	G	C	C	A	.	.	.
*Clarias_MHCI*TH41*	.	C	.	.	.	G	G	.	A	T	.	G	G	G	.	.	A	T	.	.	T	G	C	C	A	.	.	.
*Clarias_MHCI*TH42*	T	.	.	C	.	G	G	.	A	T	.	G	G	G	A	A	A	T	.	.	T	G	C	C	A	.	.	.
*Clarias_MHCI*TH43*	T	.	.	C	.	G	G	.	A	T	.	G	G	G	.	.	A	T	-	.	T	G	C	C	A	.	.	.
*Clarias_MHCI*TH44*	T	A	.	C	.	G	A	.	A	T	.	G	G	.	.	.	A	T	.	.	T	G	C	C	A	.	.	.
*Clarias_MHCI*TH45*	T	.	.	.	.	G	G	.	A	T	.	G	G	.	.	.	A	T	C	.	T	.	C	C	A	.	.	.
*Clarias_MHCI*TH46*	T	.	.	.	.	G	G	.	A	T	.	G	G	.	.	.	A	T	.	.	T	.	C	C	A	.	.	.
*Clarias_MHCI*TH47*	T	.	.	C	.	G	G	.	A	T	G	.	G	.	.	.	A	T	G	.	T	G	C	C	A	.	.	.
*Clarias_MHCI*TH48*	T	.	.	.	.	G	G	.	A	T	.	G	G	.	.	T	A	T	.	.	T	G	C	C	A	.	T	.
*Clarias_MHCI*TH49*	T	.	.	C	.	G	G	.	A	T	.	G	G	.	.	.	A	T	.	.	T	G	C	C	A	T	.	.
*Clarias_MHCI*TH50*	T	.	.	C	.	G	G	.	A	T	.	G	G	.	.	.	A	T	.	.	T	G	C	C	A	.	.	.
*Clarias_MHCI*TH51*	T	.	.	.	.	G	G	.	A	T	.	G	G	.	.	.	A	T	.	.	T	.	C	C	A	.	.	.
*Clarias_MHCI*TH52*	T	.	.	.	.	G	G	.	A	T	.	G	G	.	.	.	A	T	.	.	T	.	C	C	A	.	.	.
*Clarias_MHCI*TH53*	T	.	.	C	.	G	G	.	A	.	.	G	G	.	.	.	A	T	.	.	T	G	C	C	A	.	.	.
*Clarias_MHCI*TH54*	T	.	.	C	.	G	A	.	A	T	G	T	.	.	.	.	A	T	.	A	T	G	C	C	A	.	A	.
*Clarias_MHCI*TH55*	T	.	.	C	.	G	G	.	A	T	.	G	G	.	.	.	A	T	.	.	T	G	C	C	A	T	.	.
*Clarias_MHCI*TH56*	T	.	.	.	.	G	G	.	A	T	.	G	G	.	.	.	A	T	.	.	T	.	C	C	A	.	.	.
*Clarias_MHCI*TH57*	T	.	.	C	.	G	G	G	A	T	.	G	G	.	.	.	A	T	.	.	T	G	C	C	A	.	.	.
*Clarias_MHCI*TH58*	T	.	.	C	.	G	G	.	A	T	.	G	G	.	.	.	A	T	.	.	T	G	C	C	A	.	.	.
*Clarias_MHCI*TH59*	T	.	.	C	.	G	G	.	A	T	.	G	G	.	.	.	A	T	.	.	T	G	C	C	A	.	.	.
*Clarias_MHCI*TH60*	T	.	.	.	.	G	G	.	A	T	.	.	G	.	.	.	A	T	.	.	T	G	C	C	A	.	.	.
*Clarias_MHCI*TH61*	T	.	.	C	.	G	G	.	A	T	G	.	G	.	.	.	A	T	G	.	-	G	C	C	A	.	.	.
*Clarias_MHCI*TH62*	T	.	.	C	.	G	G	.	A	T	G	.	G	.	.	.	A	T	G	.	T	G	C	C	A	.	.	.

Allele	Variable nucleotide position (Accession number: XM53477511)																											
	**816**	**817**	**820**	**821**	**822**	**823**	**824**	**825**	**827**	**828**	**829**	**830**	**834**	**835**	**836**	**838**	**839**	**840**	**845**	**848**	**853**	**863**	**866**	**871**	**872**	**874**	**875**	**876**
*Clarias_MHCI*TH63*	T	.	.	C	.	G	G	.	A	T	.	G	G	.	.	.	A	T	.	.	T	G	C	C	A	.	.	.
*Clarias_MHCI*TH64*	T	.	.	C	.	G	G	.	A	T	G	.	G	.	.	.	A	T	G	.	T	G	C	C	A	.	.	.
*Clarias_MHCI*TH65*	T	.	.	C	.	G	G	.	A	T	G	.	G	.	.	.	A	T	T	.	T	G	C	C	A	.	.	.
*Clarias_MHCI*TH66*	T	.	.	C	.	G	G	.	A	T	G	.	G	.	.	.	A	T	T	.	T	G	C	C	A	.	.	.
*Clarias_MHCI*TH67*	T	.	.	C	.	G	G	.	A	T	.	G	G	.	.	C	A	T	.	.	T	G	C	C	A	.	.	.
*Clarias_MHCI*TH68*	T	.	.	C	.	G	G	.	A	T	G	.	G	.	.	.	A	T	G	.	T	G	C	C	A	.	.	.
*Clarias_MHCI*TH69*	T	.	.	A	.	A	G	.	A	T	.	G	G	.	.	.	A	T	.	.	A	-	-	C	A	.	.	.
*Clarias_MHCI*TH70*	T	.	.	C	.	G	G	.	A	T	G	.	G	.	.	.	A	T	G	.	T	G	C	C	A	.	.	.
*Clarias_MHCI*TH71*	T	.	.	A	.	A	G	.	A	T	.	G	G	.	.	.	A	T	.	.	T	-	-	C	A	.	.	.
*Clarias_MHCI*TH72*	T	.	.	.	.	G	G	.	A	T	.	G	G	.	.	.	A	T	.	.	T	C	C	C	A	.	.	.
*Clarias_MHCI*TH73*	T	.	.	C	C	G	G	.	A	T	.	G	.	.	.	.	A	T	.	.	T	G	C	C	A	.	.	.
*Clarias_MHCI*TH74*	T	.	.	C	.	G	G	.	A	T	.	G	G	.	.	.	A	T	.	.	T	G	C	C	A	T	.	.
*Clarias_MHCI*TH75*	T	.	.	.	.	G	G	.	A	T	.	G	.	.	.	.	A	T	.	.	T	.	C	C	A	.	.	.
*Clarias_MHCI*TH76*	T	.	.	C	.	G	G	.	A	T	.	G	.	.	.	.	A	T	.	.	T	G	C	C	A	.	.	.
*Clarias_MHCI*TH77*	T	.	.	C	.	G	G	.	A	T	.	G	G	.	.	.	A	T	.	.	T	G	C	C	A	T	.	.
*Clarias_MHCI*TH78*	T	.	.	C	.	G	G	.	A	T	.	G	G	.	.	.	A	T	.	.	T	C	C	C	A	C	.	.
*Clarias_MHCI*TH79*	T	.	.	C	.	G	G	.	A	T	.	G	G	.	A	.	A	T	.	.	T	G	C	C	A	.	.	.
*Clarias_MHCI*TH80*	T	.	.	C	.	G	G	.	A	T	.	.	G	.	.	.	A	T	.	.	A	G	C	C	A	T	.	.
*Clarias_MHCI*TH81*	T	-	T	A	C	G	G	C	G	T	G	T	G	G	.	.	A	T	T	.	C	.	C	C	G	C	.	G
*Clarias_MHCI*TH82*	T	.	T	A	.	G	G	T	T	T	G	C	G	C	C	C	A	G	T	.	A	T	C	C	A	C	T	.
*Clarias_MHCI*TH83*	T	.	.	C	.	G	G	.	A	T	.	G	G	.	.	.	A	T	.	.	T	G	C	C	A	.	.	.
*Clarias_MHCI*TH84*	T	.	.	C	.	G	G	.	A	T	.	G	G	.	.	.	A	G	-	.	T	G	C	C	A	.	.	.
*Clarias_MHCI*TH85*	T	.	.	C	.	G	G	.	A	T	.	G	G	.	.	.	A	T	.	.	T	G	.	C	A	.	.	.
*Clarias_MHCI*TH86*	T	.	T	A	.	G	G	T	T	T	G	C	G	C	C	C	A	G	T	.	A	T	C	C	A	C	T	.
*Clarias_MHCI*TH87*	T	.	.	C	.	G	G	.	A	T	.	G	G	.	.	.	A	T	.	.	T	G	C	C	A	.	.	.
*Clarias_MHCI*TH88*	T	.	.	.	.	G	G	.	A	T	.	G	G	.	.	T	A	T	.	.	T	G	C	A	A	.	T	.
*Clarias_MHCI*TH89*	T	.	.	.	.	G	G	.	A	T	.	G	G	.	.	T	A	T	.	.	T	G	C	C	A	.	T	.
*Clarias_MHCI*TH90*	T	.	.	C	.	G	G	.	A	T	.	G	G	.	.	.	A	T	G	.	T	G	C	C	A	.	.	.
*Clarias_MHCI*TH91*	T	A	.	C	.	A	G	.	A	T	.	G	G	G	.	.	A	T	.	.	T	G	C	C	A	.	.	.

Allele	Variable nucleotide position (Accession number: XM53477511)																										
	**877**	**878**	**879**	**882**	**883**	**899**	**900**	**901**	**902**	**903**	**904**	**905**	**906**	**907**	**908**	**909**	**910**	**911**	**912**	**913**	**914**	**915**	**916**	**917**	**918**	**920**	**921**
**XM53477511**	**A**	**A**	**C**	**C**	**T**	**C**	**A**	**G**	**T**	**G**	**G**	**T**	**G**	**A**	**T**	**G**	**G**	**A**	**A**	**T**	**C**	**T**	**A**	**C**	**C**	**G**	**C**
*Clarias_MHCI*TH1*	.	G	G	G	.	G	.	.	A	A	A	.	T	C	.	.	.	.	G	.	G	A	G	T	T	.	A
*Clarias_MHCI*TH2*	.	G	G	G	.	G	.	.	A	A	A	.	T	C	.	.	.	.	G	.	G	A	G	T	T	.	A
*Clarias_MHCI*TH3*	.	G	G	G	C	-	-	T	.	A	A	.	T	C	.	.	.	.	G	.	G	A	G	T	T	.	A
*Clarias_MHCI*TH4*	.	G	G	G	.	-	.	A	A	A	A	.	T	C	.	A	.	.	G	.	G	A	G	T	T	.	A
*Clarias_MHCI*TH5*	.	G	G	G	.	G	.	.	A	A	A	.	T	C	.	.	.	G	.	.	G	A	G	T	T	.	A
*Clarias_MHCI*TH6*	.	G	G	G	.	G	.	.	A	A	A	.	T	C	.	.	.	G	.	.	G	A	G	T	T	.	A
*Clarias_MHCI*TH7*	.	G	G	G	.	G	.	.	A	A	A	.	T	C	.	.	.	G	.	.	G	A	G	T	T	.	A
*Clarias_MHCI*TH8*	.	G	G	G	.	-	.	A	A	A	A	.	T	C	.	.	.	.	G	.	G	A	G	T	T	.	A
*Clarias_MHCI*TH9*	.	G	G	G	.	G	.	.	A	A	A	.	T	C	.	.	.	G	.	.	G	A	G	T	T	.	A
*Clarias_MHCI*TH10*	.	G	G	G	C	-	-	T	.	A	A	.	T	C	.	.	.	.	G	.	G	A	G	T	T	.	A
*Clarias_MHCI*TH11*	.	G	G	G	.	G	.	.	A	A	A	.	T	C	.	.	.	.	G	.	G	A	G	T	T	.	A
*Clarias_MHCI*TH12*	.	G	G	G	.	G	.	.	A	A	A	.	T	C	.	.	.	.	G	.	G	A	G	T	T	.	A
*Clarias_MHCI*TH13*	.	G	G	G	.	G	.	.	A	A	A	.	T	C	.	.	.	.	T	.	G	A	G	T	T	.	A
*Clarias_MHCI*TH14*	.	G	G	G	.	G	.	.	A	A	A	.	T	C	.	.	.	.	G	.	G	A	G	T	T	.	A
*Clarias_MHCI*TH15*	.	G	G	G	.	G	.	.	A	A	A	.	T	C	.	.	.	.	G	.	G	A	G	T	T	.	A
*Clarias_MHCI*TH16*	.	G	G	G	C	G	.	.	A	A	A	.	T	C	.	.	.	.	G	.	G	A	G	T	T	.	A
*Clarias_MHCI*TH17*	.	C	G	G	C	-	-	T	.	A	A	.	T	C	.	.	.	.	G	.	G	A	G	T	T	.	A
*Clarias_MHCI*TH18*	.	G	A	G	.	G	.	.	A	C	A	C	T	C	.	.	.	.	G	.	G	A	G	T	T	.	A
*Clarias_MHCI*TH19*	.	G	A	G	.	G	.	.	A	C	A	C	T	C	.	.	A	.	G	.	G	A	G	T	T	.	A
*Clarias_MHCI*TH20*	.	G	G	G	.	G	.	.	A	A	A	.	T	C	.	.	.	.	G	.	G	A	G	T	T	.	A
*Clarias_MHCI*TH21*	.	G	G	G	.	G	.	.	A	C	A	.	T	C	.	.	.	.	G	.	G	A	.	T	T	.	A
*Clarias_MHCI*TH22*	.	G	G	G	C	G	.	.	A	A	A	.	T	C	.	.	.	.	G	.	G	A	G	T	T	.	A
*Clarias_MHCI*TH23*	.	G	G	G	.	G	.	.	A	A	A	.	T	C	.	.	.	.	G	.	G	A	G	T	T	.	A
*Clarias_MHCI*TH24*	.	G	A	G	.	G	.	.	A	C	A	.	T	C	.	.	.	.	G	.	G	A	.	T	T	.	A
*Clarias_MHCI*TH25*	C	.	G	G	.	G	.	.	A	A	A	.	T	C	.	.	.	.	-	.	G	A	.	G	A	T	A
*Clarias_MHCI*TH26*	.	G	A	G	.	G	.	.	A	C	A	C	T	C	.	.	.	.	G	.	G	A	G	T	T	.	A
*Clarias_MHCI*TH27*	.	G	G	G	C	-	-	T	.	A	A	.	T	C	.	.	.	.	G	.	G	A	G	T	T	.	A
*Clarias_MHCI*TH28*	.	G	G	G	.	G	.	.	A	C	A	.	T	C	.	.	.	.	G	.	A	A	G	T	T	.	A
*Clarias_MHCI*TH29*	.	G	G	G	.	G	.	.	A	C	A	.	T	C	.	.	.	.	G	.	G	A	.	T	T	.	A
*Clarias_MHCI*TH30*	.	G	G	G	.	G	.	T	A	A	A	.	T	C	.	.	.	.	G	.	A	A	G	T	T	.	A

Allele	Variable nucleotide position (Accession number: XM53477511)																									
	**877**	**878**	**879**	**882**	**883**	**899**	**900**	**901**	**902**	**903**	**904**	**905**	**906**	**907**	**908**	**909**	**910**	**911**	**912**	**913**	**914**	**915**	**916**	**917**	**918**	**920**	**921**
*Clarias_MHCI*TH31*	.	G	G	G	.	G	.	.	A	C	A	.	C	C	.	.	.	.	G	.	G	A	.	T	T	.	A
*Clarias_MHCI*TH32*	.	G	G	G	.	G	.	.	A	A	A	.	T	C	.	.	.	.	G	.	G	A	G	T	T	.	A
*Clarias_MHCI*TH33*	.	G	G	G	.	G	.	.	A	C	A	.	T	C	.	.	A	.	.	G	G	A	.	A	T	.	A
*Clarias_MHCI*TH34*	.	G	A	G	.	G	.	.	A	C	A	C	T	C	.	.	.	.	G	.	G	A	G	T	T	.	A
*Clarias_MHCI*TH35*	.	G	G	G	.	G	.	.	A	A	A	.	T	C	.	.	.	.	G	.	G	A	G	T	T	.	A
*Clarias_MHCI*TH36*	.	G	G	G	.	G	.	.	A	A	A	.	T	C	.	.	.	.	G	.	G	A	G	T	T	.	A
*Clarias_MHCI*TH37*	.	G	G	G	.	G	.	.	A	A	A	.	T	C	.	.	.	.	G	.	G	A	G	T	T	.	A
*Clarias_MHCI*TH38*	C	G	G	G	.	G	.	.	A	A	A	.	T	C	.	.	.	.	-	.	G	A	.	G	A	T	A
*Clarias_MHCI*TH39*	.	G	G	G	.	G	.	.	A	A	A	.	T	C	.	.	.	.	G	.	G	A	G	T	T	.	A
*Clarias_MHCI*TH40*	.	G	G	G	.	A	.	.	A	C	A	.	T	C	.	.	.	.	G	.	G	A	.	T	T	.	A
*Clarias_MHCI*TH41*	.	G	G	G	.	G	.	.	A	C	A	.	T	C	.	.	.	.	G	.	G	A	.	T	T	.	A
*Clarias_MHCI*TH42*	.	G	G	G	.	G	.	.	A	A	A	.	T	C	.	.	.	.	G	.	G	A	G	T	T	.	A
*Clarias_MHCI*TH43*	.	G	G	G	.	G	.	.	A	A	A	.	A	C	.	.	.	.	G	.	G	A	G	T	T	.	A
*Clarias_MHCI*TH44*	.	G	G	G	.	G	.	.	A	A	A	.	T	C	.	.	.	.	G	.	G	A	G	T	T	.	A
*Clarias_MHCI*TH45*	.	G	G	G	.	G	.	.	A	A	A	.	T	C	.	.	.	G	.	.	G	A	G	T	T	.	A
*Clarias_MHCI*TH46*	.	G	G	A	.	G	.	.	A	A	A	.	T	C	.	.	.	G	.	.	G	A	G	T	T	.	A
*Clarias_MHCI*TH47*	.	G	G	G	.	G	.	.	A	A	A	.	T	C	.	.	.	.	G	.	G	A	G	T	T	.	A
*Clarias_MHCI*TH48*	.	G	G	G	.	-	.	A	A	A	A	.	T	C	.	A	.	.	G	.	G	A	G	T	T	.	A
*Clarias_MHCI*TH49*	.	G	G	G	.	-	-	.	A	A	A	.	T	C	.	.	.	.	G	.	G	A	G	T	T	.	T
*Clarias_MHCI*TH50*	.	G	G	A	.	G	.	.	A	A	A	.	T	T	.	.	.	.	G	.	G	A	G	T	T	.	A
*Clarias_MHCI*TH51*	.	G	G	G	.	G	.	.	A	A	A	.	T	C	.	.	.	G	.	.	G	A	G	T	T	.	A
*Clarias_MHCI*TH52*	.	G	T	G	.	G	.	.	A	A	A	.	T	C	.	.	.	G	.	.	G	A	G	T	T	.	A
*Clarias_MHCI*TH53*	.	G	G	G	.	G	.	.	A	A	A	G	T	C	.	.	.	.	G	.	G	A	G	T	T	.	A
*Clarias_MHCI*TH54*	.	G	A	G	.	A	.	.	A	A	A	.	C	C	.	.	.	.	.	A	G	A	G	T	T	.	A
*Clarias_MHCI*TH55*	.	G	G	G	.	-	-	.	A	A	A	.	T	C	.	.	.	.	G	.	G	A	G	T	T	.	T
*Clarias_MHCI*TH56*	.	G	G	G	.	G	.	.	A	A	A	.	T	C	.	.	.	G	.	.	G	A	G	T	T	.	A
*Clarias_MHCI*TH57*	.	G	G	G	.	G	.	.	A	A	A	.	T	C	.	.	.	G	G	.	A	A	G	T	T	.	.
*Clarias_MHCI*TH58*	.	G	T	-	-	G	.	.	A	T	A	.	T	T	.	.	.	G	C	.	G	A	G	T	T	.	A
*Clarias_MHCI*TH59*	.	G	G	G	C	G	.	.	A	A	A	.	C	.	.	.	.	.	G	.	G	A	G	T	A	.	A
*Clarias_MHCI*TH60*	.	G	G	G	.	G	.	.	A	A	A	.	T	C	.	.	.	.	G	.	G	A	G	T	T	.	A
*Clarias_MHCI*TH61*	.	G	G	G	.	G	.	.	A	A	A	.	T	C	.	.	.	.	G	.	G	A	G	T	T	A	A

Allele	Variable nucleotide position (Accession number: XM53477511)																									
	**877**	**878**	**879**	**882**	**883**	**899**	**900**	**901**	**902**	**903**	**904**	**905**	**906**	**907**	**908**	**909**	**910**	**911**	**912**	**913**	**914**	**915**	**916**	**917**	**918**	**920**	**921**
*Clarias_MHCI*TH62*	.	G	G	G	.	G	.	.	A	A	A	.	T	C	.	.	.	.	G	.	G	A	G	T	T	.	A
*Clarias_MHCI*TH63*	.	G	G	G	.	G	.	.	A	A	A	G	T	C	.	.	.	.	G	.	G	A	G	T	T	.	A
*Clarias_MHCI*TH64*	.	G	G	G	.	G	C	.	A	A	A	.	T	C	.	.	.	.	G	.	G	A	G	T	T	A	A
*Clarias_MHCI*TH65*	.	G	G	G	.	G	.	.	A	A	A	.	T	C	.	.	.	.	G	.	G	A	G	T	T	.	A
*Clarias_MHCI*TH66*	.	G	G	G	.	G	C	.	A	A	A	.	T	C	.	.	.	.	G	.	G	A	G	T	T	.	A
*Clarias_MHCI*TH67*	.	G	G	G	.	G	G	.	A	A	A	.	T	C	.	.	.	.	T	.	G	A	G	T	T	.	A
*Clarias_MHCI*TH68*	.	G	G	G	.	G	.	.	A	A	A	.	T	C	.	.	.	.	G	.	G	A	G	T	T	A	A
*Clarias_MHCI*TH69*	.	G	G	G	C	-	-	T	.	A	A	.	T	C	.	.	.	.	G	.	G	A	G	T	T	.	A
*Clarias_MHCI*TH70*	.	G	G	G	.	G	.	.	A	A	A	.	T	C	.	.	.	.	G	.	G	A	G	T	T	A	A
*Clarias_MHCI*TH71*	.	G	G	G	C	-	-	T	.	A	A	.	T	C	.	.	.	.	G	.	G	A	G	T	T	.	A
*Clarias_MHCI*TH72*	.	G	G	G	.	G	.	.	A	A	A	.	T	C	.	.	.	G	.	.	G	A	G	T	T	.	A
*Clarias_MHCI*TH73*	.	.	G	G	C	G	.	.	A	A	A	.	T	C	.	.	.	.	G	.	G	A	G	T	T	.	A
*Clarias_MHCI*TH74*	.	G	G	G	.	-	-	.	A	A	A	.	T	C	.	.	.	.	G	.	G	A	G	T	T	.	T
*Clarias_MHCI*TH75*	.	G	G	G	.	G	.	.	A	A	A	.	T	C	.	.	.	G	.	.	G	A	G	T	T	.	A
*Clarias_MHCI*TH76*	.	.	G	G	C	G	.	.	A	A	A	.	T	C	.	.	.	.	G	.	G	A	G	T	T	.	A
*Clarias_MHCI*TH77*	.	G	G	G	.	G	.	.	A	A	A	.	T	C	.	.	.	G	G	.	G	A	G	T	T	.	A
*Clarias_MHCI*TH78*	.	G	G	G	.	G	.	.	A	A	A	.	T	C	.	.	.	.	.	.	G	A	T	T	T	.	A
*Clarias_MHCI*TH79*	.	G	T	-	-	G	.	.	A	T	A	.	T	T	.	.	.	G	C	.	G	A	G	T	T	.	A
*Clarias_MHCI*TH80*	.	G	G	G	.	G	.	.	A	A	A	.	T	C	.	.	.	.	T	.	G	A	.	T	T	.	T
*Clarias_MHCI*TH81*	.	G	G	T	.	A	.	T	G	C	A	.	T	T	A	.	C	.	G	.	T	A	T	.	.	C	A
*Clarias_MHCI*TH82*	.	G	.	G	G	G	.	C	A	T	A	.	T	C	C	.	A	T	G	A	T	C	G	T	.	.	A
*Clarias_MHCI*TH83*	.	G	T	-	-	G	.	.	A	T	A	.	T	T	.	.	.	G	C	.	G	A	G	T	T	.	A
*Clarias_MHCI*TH84*	.	G	T	-	-	G	.	.	A	T	A	.	T	T	.	.	.	G	C	.	G	A	G	T	T	.	A
*Clarias_MHCI*TH85*	.	G	A	G	.	G	.	.	A	A	A	.	T	C	.	.	.	.	G	.	G	A	G	T	T	.	A
*Clarias_MHCI*TH86*	.	G	.	G	G	G	.	C	A	T	A	.	T	C	C	.	A	T	G	A	T	C	G	T	.	.	A
*Clarias_MHCI*TH87*	.	G	A	G	.	G	.	.	A	A	A	.	T	C	.	.	.	.	G	.	G	A	G	T	T	.	A
*Clarias_MHCI*TH88*	.	G	G	G	.	-	.	A	A	A	A	.	T	C	.	A	.	.	G	.	G	A	G	T	T	.	A
*Clarias_MHCI*TH89*	.	G	G	G	C	-	.	A	A	A	A	.	T	C	.	.	.	.	G	.	G	A	G	T	T	.	A
*Clarias_MHCI*TH90*	.	G	G	A	.	G	.	.	A	A	A	.	T	T	.	.	.	.	G	.	G	A	G	T	T	.	A
*Clarias_MHCI*TH91*	.	G	G	G	.	G	.	.	A	C	A	.	T	C	.	.	.	.	G	.	A	A	G	T	T	.	A

Allele	Variable nucleotide position (Accession number: XM53477511)																										
	**924**	**928**	**929**	**947**	**948**	**949**	**952**	**955**	**957**	**963**	**968**	**974**	**976**	**978**	**981**	**984**	**985**	**987**	**988**	**989**	**990**	**991**	**992**	**993**	**994**	**995**	**996**
**XM53477511**	**G**	**G**	**A**	**A**	**A**	**G**	**C**	**A**	**A**	**A**	**T**	**G**	**G**	**C**	**A**	**G**	**C**	**C**	**C**	**T**	**T**	**T**	**A**	**A**	**A**	**G**	**A**
*Clarias_MHCI*TH1*	T	.	T	.	G	A	G	G	G	G	.	A	A	T	.	.	G	.	A	.	G	.	T	G	G	.	G
*Clarias_MHCI*TH2*	T	.	T	.	G	A	T	.	G	G	.	A	A	T	.	.	G	.	A	.	G	.	T	G	G	.	G
*Clarias_MHCI*TH3*	A	.	T	.	.	T	T	G	G	G	.	A	T	T	.	.	G	.	A	.	G	.	T	G	G	.	G
*Clarias_MHCI*TH4*	T	.	T	.	G	A	T	G	G	G	.	A	A	T	.	.	G	.	A	.	G	.	T	G	G	.	G
*Clarias_MHCI*TH5*	T	A	T	.	G	A	T	G	G	G	C	A	A	T	.	C	.	.	A	.	G	.	T	G	G	.	G
*Clarias_MHCI*TH6*	T	.	T	.	G	A	T	G	G	G	.	A	A	T	.	.	G	.	A	.	G	.	T	G	G	.	G
*Clarias_MHCI*TH7*	T	.	T	.	G	A	T	G	G	G	C	A	A	T	.	.	G	.	A	.	G	.	T	G	G	.	G
*Clarias_MHCI*TH8*	T	.	T	.	G	A	T	G	G	G	.	A	A	T	.	.	G	.	A	.	G	.	T	G	G	.	G
*Clarias_MHCI*TH9*	T	A	T	.	G	A	T	G	G	G	C	A	A	T	.	C	.	.	A	.	G	.	T	G	G	.	G
*Clarias_MHCI*TH10*	A	.	T	.	.	T	T	G	G	G	.	A	A	T	.	.	G	.	A	.	G	.	T	G	G	.	G
*Clarias_MHCI*TH11*	T	.	T	.	G	A	T	G	G	G	.	A	A	T	.	.	G	.	A	.	G	.	T	G	G	.	G
*Clarias_MHCI*TH12*	T	.	T	.	G	A	T	.	G	G	.	A	A	T	.	.	G	.	A	.	G	.	T	G	G	.	G
*Clarias_MHCI*TH13*	T	.	T	.	G	A	T	G	G	G	.	A	A	T	.	.	G	.	A	.	G	.	T	G	G	.	G
*Clarias_MHCI*TH14*	T	.	T	.	G	A	T	.	G	G	.	A	A	T	.	.	G	.	A	.	G	.	T	G	G	.	G
*Clarias_MHCI*TH15*	T	.	T	.	G	A	T	.	G	G	.	A	A	T	.	.	G	.	A	.	G	.	T	G	G	-	G
*Clarias_MHCI*TH16*	T	.	T	.	G	A	G	G	G	G	.	A	A	T	.	.	G	.	A	.	G	.	T	G	G	.	G
*Clarias_MHCI*TH17*	A	.	T	.	.	A	T	-	-	-	.	A	A	T	.	.	G	.	A	.	C	.	T	G	G	.	G
*Clarias_MHCI*TH18*	T	.	T	.	G	A	T	G	G	G	.	A	A	A	.	.	A	.	A	.	G	.	T	G	G	.	G
*Clarias_MHCI*TH19*	T	.	T	.	G	A	T	G	G	G	.	A	A	G	.	.	A	.	A	.	G	.	T	G	G	.	G
*Clarias_MHCI*TH20*	T	.	T	.	G	A	T	-	G	G	.	.	A	T	.	.	G	.	A	.	A	.	T	G	G	.	G
*Clarias_MHCI*TH21*	T	.	G	.	G	A	T	G	G	G	.	A	A	T	.	.	G	.	A	.	G	.	T	G	G	.	G
*Clarias_MHCI*TH22*	T	.	T	.	G	A	G	G	.	G	.	A	A	T	.	.	G	.	A	.	G	.	T	G	G	.	G
*Clarias_MHCI*TH23*	T	.	T	.	G	A	T	G	G	G	.	A	A	T	.	.	G	.	A	.	G	.	T	G	G	.	G
*Clarias_MHCI*TH24*	T	.	G	.	G	A	T	G	G	G	.	A	A	T	.	.	A	.	A	.	G	.	T	G	G	.	G
*Clarias_MHCI*TH25*	A	.	T	.	G	A	T	.	G	.	.	A	A	T	.	.	G	.	A	.	G	.	T	G	G	.	G
*Clarias_MHCI*TH26*	T	.	T	.	G	A	T	G	G	G	.	A	A	G	.	.	A	.	A	.	G	.	T	G	G	.	G
*Clarias_MHCI*TH27*	A	.	T	.	.	A	T	-	-	-	.	A	A	T	.	.	G	.	A	.	C	.	T	G	G	.	G
*Clarias_MHCI*TH28*	T	.	T	.	G	A	T	G	G	G	.	A	A	T	.	.	G	.	A	.	G	.	T	G	G	.	G
*Clarias_MHCI*TH29*	T	.	G	.	G	A	T	G	G	G	.	A	A	T	.	.	G	.	A	.	G	.	T	G	G	.	G
*Clarias_MHCI*TH30*	T	.	T	.	G	A	G	T	G	G	.	A	A	T	.	.	G	.	A	.	G	.	T	G	G	.	G

Allele	Variable nucleotide position (Accession number: XM53477511)																									
	924	928	929	947	948	949	952	955	957	963	968	974	976	978	981	984	985	987	988	989	990	991	992	993	994	995	996
*Clarias_MHCI*TH31*	T	.	G	.	G	A	T	G	G	G	.	A	A	T	.	.	G	.	A	.	G	.	T	G	G	.	G
*Clarias_MHCI*TH32*	T	.	T	.	G	A	T	-	G	G	.	.	A	T	.	.	G	.	A	.	A	.	T	G	G	.	G
*Clarias_MHCI*TH33*	T	.	G	.	G	A	T	G	G	G	.	A	A	T	.	.	G	.	A	.	G	.	T	G	G	.	G
*Clarias_MHCI*TH34*	T	.	T	.	G	A	T	G	G	G	.	A	A	G	.	.	A	.	A	.	G	.	T	G	G	.	G
*Clarias_MHCI*TH35*	T	.	T	.	G	A	T	-	G	G	.	.	A	T	.	.	G	.	A	.	A	.	T	G	G	.	G
*Clarias_MHCI*TH36*	A	.	T	.	G	A	T	.	G	G	.	A	A	T	.	.	G	A	A	.	G	.	T	G	G	.	G
*Clarias_MHCI*TH37*	T	.	G	.	G	A	T	G	G	G	.	A	A	T	.	.	G	.	A	.	G	.	T	G	G	.	G
*Clarias_MHCI*TH38*	A	.	T	.	G	A	T	.	G	.	.	A	A	T	.	.	G	.	A	.	G	.	T	G	G	.	G
*Clarias_MHCI*TH39*	T	.	T	.	G	A	T	.	G	G	C	A	A	T	.	.	G	.	A	.	G	.	T	G	G	.	G
*Clarias_MHCI*TH40*	T	.	G	.	G	A	T	.	G	G	.	A	A	T	.	.	A	.	A	.	G	.	T	G	G	.	G
*Clarias_MHCI*TH41*	T	.	G	.	G	A	T	G	G	G	.	A	A	T	.	.	G	.	A	.	G	.	T	G	G	.	G
*Clarias_MHCI*TH42*	T	.	T	.	G	A	T	G	G	.	.	A	A	T	.	.	G	.	A	.	G	.	T	G	G	.	G
*Clarias_MHCI*TH43*	T	.	T	.	.	A	T	G	G	G	.	A	A	T	.	A	A	.	A	.	G	.	T	G	G	.	G
*Clarias_MHCI*TH44*	T	.	T	.	G	A	T	-	G	G	.	.	A	T	.	.	G	.	A	.	A	.	T	G	G	.	G
*Clarias_MHCI*TH45*	T	.	T	.	G	A	T	G	G	G	C	A	A	T	.	.	G	.	A	.	G	.	T	G	G	.	G
*Clarias_MHCI*TH46*	T	.	T	.	G	A	T	G	G	G	C	A	A	T	.	.	G	.	A	.	G	.	T	G	G	.	G
*Clarias_MHCI*TH47*	T	.	T	.	G	.	T	G	G	.	.	A	A	T	.	.	A	.	A	.	G	.	T	G	G	.	G
*Clarias_MHCI*TH48*	T	.	T	.	G	A	T	G	G	G	.	A	A	T	.	.	G	A	A	.	G	.	T	G	G	.	G
*Clarias_MHCI*TH49*	T	.	T	G	G	A	T	G	G	G	.	A	A	T	.	-	G	.	A	.	G	.	T	G	G	.	G
*Clarias_MHCI*TH50*	T	.	T	.	.	A	T	G	G	G	.	A	A	T	.	.	G	.	A	.	G	.	T	G	G	A	G
*Clarias_MHCI*TH51*	T	.	T	.	G	A	T	G	G	G	C	A	A	T	.	.	G	.	A	.	G	.	T	G	G	.	G
*Clarias_MHCI*TH52*	T	.	T	.	G	A	T	G	G	G	C	A	A	T	.	.	G	.	A	.	G	.	T	G	G	.	G
*Clarias_MHCI*TH53*	T	.	T	.	G	A	T	G	.	G	.	A	A	T	.	.	G	.	A	.	G	.	T	T	G	.	G
*Clarias_MHCI*TH54*	T	.	T	.	G	.	T	G	G	G	.	A	A	T	.	.	G	.	A	.	G	.	T	G	G	.	G
*Clarias_MHCI*TH55*	T	.	T	G	C	A	T	G	G	G	.	A	A	T	.	-	G	.	A	.	G	.	T	G	G	.	G
*Clarias_MHCI*TH56*	T	.	T	.	G	A	T	G	G	G	C	A	A	T	.	C	.	.	A	.	G	.	T	G	G	.	G
*Clarias_MHCI*TH57*	T	.	T	.	G	A	T	G	.	G	.	A	A	T	.	.	G	.	A	.	G	.	T	G	G	.	G
*Clarias_MHCI*TH58*	T	.	T	T	G	A	T	G	G	G	.	A	A	T	.	.	G	.	A	.	G	.	T	G	G	.	G
*Clarias_MHCI*TH59*	T	.	T	.	T	A	T	G	G	-	-	A	A	T	.	.	G	.	A	.	G	.	T	G	G	.	G
*Clarias_MHCI*TH60*	T	A	T	.	G	A	T	G	G	-	.	A	A	T	.	.	G	A	A	.	G	.	T	T	G	.	G
*Clarias_MHCI*TH61*	T	.	T	.	G	A	T	G	G	G	.	A	A	T	.	.	A	.	A	.	G	.	T	G	G	.	G

Allele	Variable nucleotide position (Accession number: XM53477511)																										
	**924**	**928**	**929**	**947**	**948**	**949**	**952**	**955**	**957**	**963**	**968**	**974**	**976**	**978**	**981**	**984**	**985**	**987**	**988**	**989**	**990**	**991**	**992**	**993**	**994**	**995**	**996**
*Clarias_MHCI*TH62*	T	.	T	.	G	A	T	G	G	.	.	A	A	T	.	.	A	.	A	.	G	.	T	G	G	.	G
*Clarias_MHCI*TH63*	T	.	T	.	G	A	T	G	.	G	.	A	A	T	.	.	G	.	A	.	G	.	T	T	G	.	G
*Clarias_MHCI*TH64*	T	.	T	.	G	.	T	G	G	.	.	A	A	T	.	.	A	.	A	.	G	.	T	G	G	.	G
*Clarias_MHCI*TH65*	T	.	T	.	G	.	T	G	G	.	.	A	A	T	.	.	A	.	A	.	G	.	T	G	G	.	G
*Clarias_MHCI*TH66*	T	.	T	.	G	.	T	G	G	.	.	A	A	T	.	.	A	.	A	.	G	.	T	G	G	.	G
*Clarias_MHCI*TH67*	T	.	T	.	G	A	A	G	.	G	.	A	A	T	.	.	G	.	A	.	G	.	T	G	G	.	G
*Clarias_MHCI*TH68*	T	.	T	.	G	.	T	G	G	.	.	A	A	T	.	.	A	.	A	.	G	.	T	G	G	.	G
*Clarias_MHCI*TH69*	A	.	T	.	.	T	T	G	G	G	.	A	T	T	.	.	G	.	A	.	G	.	T	G	G	.	G
*Clarias_MHCI*TH70*	T	.	T	.	G	A	T	G	G	.	.	A	A	T	.	.	A	.	A	.	G	.	T	G	G	.	G
*Clarias_MHCI*TH71*	A	.	T	.	.	T	T	G	G	G	.	A	T	T	T	-	-	A	A	.	G	.	T	G	G	.	G
*Clarias_MHCI*TH72*	T	A	T	.	G	A	T	G	G	G	C	A	A	T	.	C	.	.	A	.	G	.	T	G	G	.	G
*Clarias_MHCI*TH73*	T	.	T	-	-	A	T	G	G	G	.	A	A	T	.	.	G	.	A	.	G	.	T	G	G	.	G
*Clarias_MHCI*TH74*	T	.	T	.	G	A	T	G	G	G	.	A	A	T	.	-	G	.	A	.	G	.	T	G	G	.	G
*Clarias_MHCI*TH75*	T	.	T	.	G	A	T	G	G	G	C	A	A	T	.	.	G	.	A	.	G	.	T	G	G	.	G
*Clarias_MHCI*TH76*	T	.	T	-	-	A	T	G	G	G	.	A	A	T	.	.	G	.	A	.	G	.	T	G	G	.	G
*Clarias_MHCI*TH77*	T	-	-	-	-	-	T	G	G	G	.	A	A	T	.	.	G	.	A	.	G	.	T	G	G	.	G
*Clarias_MHCI*TH78*	T	.	T	-	-	-	-	-	-	G	.	A	A	T	.	.	G	.	A	.	G	.	T	G	G	.	G
*Clarias_MHCI*TH79*	T	.	T	T	G	A	T	G	G	G	.	A	A	T	.	.	G	.	A	.	G	.	T	G	G	.	G
*Clarias_MHCI*TH80*	T	.	T	.	G	A	T	G	G	G	.	A	A	T	.	.	G	T	A	.	C	.	T	G	G	.	G
*Clarias_MHCI*TH81*	T	T	T	T	G	A	T	.	G	.	.	A	-	-	.	A	A	T	A	.	C	A	G	G	G	C	G
*Clarias_MHCI*TH82*	C	.	.	.	.	T	A	C	.	.	A	A	A	G	T	.	A	.	A	G	C	A	C	C	.	T	C
*Clarias_MHCI*TH83*	T	.	T	.	G	A	T	G	G	G	.	A	A	T	.	.	G	.	A	.	G	.	T	G	G	.	G
*Clarias_MHCI*TH84*	T	.	T	.	G	A	T	G	G	G	.	A	A	T	.	.	G	.	A	.	G	.	T	G	G	.	G
*Clarias_MHCI*TH85*	T	.	T	.	G	A	T	G	G	G	.	A	A	T	.	.	G	.	A	.	G	.	T	G	G	.	G
*Clarias_MHCI*TH86*	C	.	.	.	.	T	A	C	.	.	A	A	A	G	T	.	A	.	A	G	.	A	C	C	T	.	.
*Clarias_MHCI*TH87*	T	.	T	.	G	A	T	G	G	G	.	A	A	T	.	.	G	.	A	.	G	.	T	G	G	.	G
*Clarias_MHCI*TH88*	T	.	T	.	G	A	T	G	G	G	.	A	A	T	.	.	G	A	A	.	G	.	T	G	G	.	G
*Clarias_MHCI*TH89*	T	.	T	.	G	A	T	G	G	G	.	A	A	T	.	.	G	.	A	.	G	.	T	G	G	.	G
*Clarias_MHCI*TH90*	T	.	T	.	.	A	T	G	G	G	.	A	A	T	.	.	G	.	A	.	G	.	T	G	G	A	G
*Clarias_MHCI*TH91*	T	.	T	.	G	A	T	G	G	G	.	A	A	T	.	.	T	.	A	.	G	.	T	G	G	.	G

Table S4. The results of Analysis of Variance (AMOVA) for catfish populations in Thailand.

**Source**	**df**	**SS**	**Var**	**% of variance**
Among Population	19	97005.408	5105.548	11
Among Individual	761	386266.892	507.578	43
Within Individual	1,108	405959.503	366.389	46
Total	1,888	889231.803	5979.515	100

(df: degree of freedom *f*; SS: sum of squares; Var: variance components)

Table S5. Detailed site-by-site results from the MEME analysis.

Codon	α	β1	p1	β+	p+	LRT	p-value	q	Class
1	0.000	0.000	1	0.000	0.000	0.000	1.000	1.000	Invariable
2	0.324	0.012	0	99.367	1.000	5.554	0.028	0.053	Diversifying
3	0.032	0.022	0	23.997	1.000	28.423	0.000	0.000	Diversifying
4	0.075	0.075	0	2524.722	1.000	30.544	0.000	0.000	Diversifying
5	0.142	0.142	0	20.061	1.000	13.923	0.000	0.002	Diversifying
6	0.074	0.050	0	458.768	1.000	26.945	0.000	0.000	Diversifying
7	0.181	0.181	0	10.736	1.000	14.184	0.000	0.002	Diversifying
8	0.027	0.025	0	4.783	1.000	13.567	0.000	0.002	Diversifying
9	0.658	0.658	0	28395.742	1.000	24.585	0.000	0.000	Diversifying
10	0.058	0.000	0	0.468	1.000	4.451	0.050	0.082	Diversifying
11	0.585	0.585	0	0.588	1.000	0.000	0.667	0.692	Neutral
12	0.158	0.000	0	0.609	1.000	2.597	0.132	0.174	Neutral
13	0.446	0.446	0	75.077	1.000	17.820	0.000	0.000	Diversifying
14	0.177	0.177	0	20.133	1.000	19.874	0.000	0.000	Diversifying
15	0.059	0.059	0	1.656	1.000	3.819	0.070	0.101	Diversifying
16	0.078	0.074	0	8.254	1.000	7.036	0.013	0.028	Diversifying
17	1.548	0.640	0	4.820	1.000	0.498	0.430	0.494	Neutral
18	0.852	0.446	0	2.280	1.000	0.639	0.394	0.462	Neutral
19	0.017	0.011	0	17.555	1.000	3.862	0.068	0.102	Diversifying
20	0.406	0.079	0	3.478	1.000	6.947	0.014	0.027	Diversifying
21	0.077	0.077	0	11934.451	1.000	25.552	0.000	0.000	Diversifying
22	0.059	0.044	0	7.233	1.000	9.113	0.005	0.012	Diversifying
23	0.058	0.046	0	30.651	1.000	24.838	0.000	0.000	Diversifying
24	0.483	0.483	0	15156.562	1.000	19.356	0.000	0.000	Diversifying
25	1.073	0.000	0	1.717	1.000	0.386	0.463	0.520	Neutral
26	1.462	0.754	0	5.023	1.000	0.842	0.348	0.418	Neutral
27	0.262	0.262	0	21.700	1.000	8.268	0.007	0.017	Diversifying
28	2.806	1.253	0	7475.149	1.000	8.787	0.005	0.013	Diversifying
29	1.058	1.058	0	1543.958	1.000	12.849	0.001	0.002	Diversifying

Codon	α	β1	p1	β+	p+	LRT	p-value	q	Class
30	1.471	0.778	0	2.462	1.000	0.147	0.551	0.607	Neutral
31	1.309	0.065	0	1.522	1.000	0.089	0.580	0.614	Neutral
32	0.748	0.404	0	2.500	1.000	1.147	0.292	0.375	Neutral
33	0.890	0.610	0	263.264	1.000	9.961	0.003	0.009	Diversifying
34	1.397	0.645	0	2.792	1.000	0.100	0.574	0.620	Neutral
35	0.664	0.149	0	4.254	1.000	4.225	0.056	0.089	Diversifying
36	1.267	0.354	0	11.567	1.000	4.023	0.063	0.096	Diversifying
37	1.292	0.735	0	13.975	1.000	5.224	0.034	0.059	Diversifying
38	0.732	0.348	0	17.844	1.000	5.542	0.029	0.051	Diversifying
39	0.687	0.621	0	76.755	1.000	7.706	0.009	0.021	Diversifying
40	2.142	1.194	0	17.432	1.000	1.051	0.308	0.387	Neutral
41	0.581	0.378	0	22.749	1.000	13.548	0.000	0.002	Diversifying
42	0.232	0.232	0	31.152	1.000	12.675	0.001	0.002	Diversifying
43	2.171	0.236	0	6.373	1.000	3.773	0.071	0.101	Diversifying
44	1.669	0.321	0	4.810	1.000	0.984	0.321	0.393	Neutral
45	3.739	1.103	0	1033.742	1.000	4.489	0.049	0.083	Diversifying
46	0.372	0.372	0	24.507	1.000	7.591	0.010	0.022	Diversifying
47	0.000	0.000	0	1976.037	1.000	9.068	0.005	0.012	Diversifying
48	0.217	0.011	0	5.367	1.000	3.625	0.077	0.107	Diversifying
49	0.002	0.001	0	16.197	1.000	20.558	0.000	0.000	Diversifying
50	0.015	0.008	0	23.565	1.000	36.753	0.000	0.000	Diversifying
51	0.000	0.000	0	10.522	1.000	10.635	0.002	0.006	Diversifying
52	0.017	0.006	0	8.695	1.000	6.959	0.014	0.028	Diversifying
53	0.000	0.000	0	1.148	1.000	2.763	0.121	0.163	Neutral
54	1.567	0.000	0	0.009	1.000	0.000	0.667	0.679	Neutral

Table S6. Detailed site-by-site results from the FEL analysis.

Codon	α	β	α = β	LRT	p-value	Total branch length	Class
1	0.000	0.000	0.000	0.000	1.000	0.000	Invariable
2	6.215	0.326	0.428	8.806	0.003	30.395	Purifying
3	0.717	0.569	0.597	0.137	0.711	42.427	Neutral
4	0.063	0.437	0.317	3.329	0.068	22.535	Diversifying
5	2.048	1.041	1.204	0.578	0.447	85.580	Neutral
6	0.697	1.007	0.936	0.385	0.535	66.538	Neutral
7	0.783	0.889	0.865	0.033	0.855	61.473	Neutral
8	0.665	0.866	0.843	0.084	0.772	59.929	Neutral
9	0.658	1.060	1.010	0.296	0.587	71.834	Neutral
10	0.045	0.404	0.298	5.009	0.025	21.161	Diversifying
11	0.497	0.493	0.494	0.000	0.989	35.091	Neutral
12	0.076	0.489	0.366	3.649	0.056	26.039	Diversifying
13	0.149	0.914	0.730	4.080	0.043	51.887	Diversifying
14	0.504	1.516	1.327	0.858	0.354	94.363	Neutral
15	0.003	0.875	0.717	2.348	0.125	50.995	Neutral
16	1.018	0.948	0.962	0.013	0.910	68.412	Neutral
17	1.437	0.815	0.967	1.391	0.238	68.731	Neutral
18	0.761	0.984	0.929	0.202	0.653	66.055	Neutral
19	1.479	1.085	1.156	0.249	0.618	82.198	Neutral
20	0.658	1.057	0.966	0.571	0.450	68.671	Neutral
21	0.753	0.617	0.654	0.150	0.699	46.521	Neutral
22	0.631	0.990	0.892	0.600	0.438	63.409	Neutral
23	0.836	0.575	0.638	0.464	0.496	45.378	Neutral
24	0.561	0.748	0.703	0.212	0.645	50.004	Neutral
25	1.052	1.100	1.090	0.005	0.944	77.487	Neutral
26	1.588	1.190	1.263	0.238	0.626	89.763	Neutral
27	1.398	1.268	1.285	0.014	0.907	91.337	Neutral
28	3.803	1.204	1.363	2.325	0.127	96.902	Neutral
29	1.289	1.546	1.511	0.074	0.786	107.390	Neutral

Codon	α	β	α = β	LRT	p-value	Total branch length	Class
30	1.261	1.212	1.222	0.005	0.946	86.854	Neutral
31	1.066	1.217	1.174	0.065	0.799	83.426	Neutral
32	0.757	0.909	0.882	0.053	0.818	62.670	Neutral
33	2.001	0.995	1.161	1.382	0.240	82.521	Neutral
34	1.216	0.898	0.954	0.254	0.614	67.817	Neutral
35	0.839	0.632	0.668	0.152	0.697	47.522	Neutral
36	2.145	0.981	1.179	1.521	0.218	83.810	Neutral
37	1.571	1.165	1.263	0.306	0.580	89.793	Neutral
38	1.267	1.010	1.083	0.254	0.615	76.982	Neutral
39	0.948	1.070	1.030	0.065	0.799	73.224	Neutral
40	2.039	1.520	1.681	0.319	0.572	119.473	Neutral
41	1.220	1.764	1.578	0.389	0.533	112.189	Neutral
42	2.142	2.396	2.324	0.011	0.917	165.207	Neutral
43	2.294	2.327	2.318	0.000	0.983	164.811	Neutral
44	1.788	1.411	1.470	0.104	0.747	104.512	Neutral
45	5.998	1.152	1.471	4.771	0.029	104.597	Purifying
46	1.099	1.205	1.183	0.019	0.890	84.092	Neutral
47	2.080	1.292	1.511	0.879	0.348	107.417	Neutral
48	0.179	4.327	2.895	3.634	0.057	205.837	Diversifying
49	0.000	2.845	2.105	7.042	0.008	149.649	Diversifying
50	0.000	2.981	2.281	4.395	0.036	162.174	Diversifying
51	0.000	1.423	1.073	2.831	0.093	76.251	Diversifying
52	0.000	2.176	1.784	0.682	0.409	126.818	Neutral
53	0.000	0.440	0.412	0.190	0.663	29.269	Neutral
54	0.034	0.036	0.000	–0.014	1.000	0.001	Neutral

Table S7. Codon sites under selection identified by FUBAR analysis.

Codon	Partition	α	β	β-α	Prob[α>β]	Prob[α<β]	BayesFactor[α<β]
1	1	0.982	0.330	–0.652	0.979	0.009	0.010
2	1	10.089	0.374	–9.715	0.984	0.010	0.012
3	1	0.732	0.682	–0.050	0.505	0.387	0.709
4	1	0.167	0.466	0.299	0.063	0.895	9.545
5	1	1.901	1.113	–0.788	0.429	0.218	0.313
6	1	0.814	0.983	0.169	0.184	0.619	1.822
7	1	0.863	1.019	0.155	0.197	0.576	1.527
8	1	0.896	0.904	0.008	0.337	0.497	1.108
9	1	0.930	1.074	0.144	0.149	0.608	1.737
10	1	0.115	0.438	0.323	0.024	0.953	22.815
11	1	0.520	0.509	–0.010	0.443	0.446	0.902
12	1	0.163	0.535	0.372	0.038	0.936	16.332
13	1	0.437	0.905	0.468	0.059	0.896	9.636
14	1	1.912	1.206	–0.705	0.212	0.460	0.954
15	1	0.479	0.854	0.375	0.120	0.816	4.965
16	1	1.080	1.033	–0.047	0.313	0.363	0.639
17	1	1.234	0.990	–0.243	0.491	0.191	0.264
18	1	0.894	1.024	0.130	0.203	0.539	1.310
19	1	1.260	1.107	–0.154	0.288	0.292	0.464
20	1	0.779	1.006	0.227	0.152	0.660	2.175
21	1	0.755	0.690	–0.065	0.525	0.357	0.622
22	1	0.780	0.975	0.194	0.175	0.648	2.068
23	1	0.774	0.676	–0.098	0.567	0.320	0.528
24	1	0.668	0.787	0.119	0.278	0.608	1.738
25	1	0.914	1.042	0.128	0.197	0.525	1.238
26	1	1.340	1.166	–0.174	0.241	0.272	0.418
27	1	1.307	1.163	–0.145	0.223	0.370	0.660
28	1	4.394	1.155	–3.239	0.759	0.060	0.071
29	1	1.297	1.218	–0.079	0.165	0.322	0.533

Codon	Partition	α	β	β-α	Prob[α>β]	Prob[α<β]	BayesFactor[α<β]
30	1	1.161	1.138	–0.023	0.198	0.321	0.530
31	1	1.090	1.155	0.065	0.135	0.362	0.637
32	1	0.825	0.941	0.115	0.255	0.560	1.425
33	1	1.725	1.069	–0.656	0.523	0.151	0.200
34	1	1.116	0.988	–0.128	0.370	0.361	0.633
35	1	0.807	0.737	–0.070	0.488	0.401	0.750
36	1	1.619	1.071	–0.548	0.466	0.185	0.255
37	1	1.418	1.163	–0.256	0.281	0.194	0.270
38	1	1.177	1.104	–0.072	0.275	0.230	0.335
39	1	1.013	1.087	0.074	0.195	0.406	0.766
40	1	1.690	1.195	–0.495	0.396	0.117	0.148
41	1	1.069	1.673	0.603	0.074	0.487	1.066
42	1	1.366	2.739	1.373	0.063	0.746	3.302
43	1	1.784	2.789	1.004	0.036	0.570	1.490
44	1	1.573	1.209	–0.364	0.314	0.239	0.353
45	1	4.936	1.140	–3.796	0.824	0.035	0.041
46	1	1.128	1.130	0.001	0.196	0.386	0.705
47	1	1.947	1.190	–0.757	0.526	0.089	0.109
48	1	0.698	4.256	3.558	0.002	0.948	20.327
49	1	0.427	2.781	2.353	0.006	0.964	30.339
50	1	0.754	2.508	1.754	0.038	0.881	8.345
51	1	0.629	1.130	0.501	0.097	0.757	3.488
52	1	0.994	1.463	0.469	0.172	0.620	1.833
53	1	2.335	0.648	–1.686	0.455	0.458	0.946
54	1	4.662	0.862	–3.800	0.524	0.382	0.695

Table S8. Types of single nucleotide polymorphism (SNPs) and their locations in the partial fragment of the exon 6 of *MHC I* gene of catfish catfish populations in Thailand compare with the reference sequence (Accession number XM53477511) .

No	Allele-specific	Variable nucleotide position and nucleotide substitution	Mutation type	Amino acid change (substitution)
1	*Clarias_MHCI*TH1-16,18-20,22,25,26,29,30,32,34-39,42,43,45-90*	816C→T	Missense	Arg to Trp (CGG to TGG)
2	*Clarias_MHCI*TH17,27,28,44,91*	816C→T	Nonsense	Arg to Stop (CGG to TAG)
3	*Clarias_MHCI*TH17,27,28,44,91*	817G→A	Nonsense	Arg to Stop (CGG to TAG)
4	*Clarias_MHCI*TH21,23,24,29,31,33,40,41*	817G→C	Missense	Arg to Pro (CGG to CCG)
5	*Clarias_MHCI*TH81,82,86*	820G→T	Missense	Gly to Val (GGT to GTA)
6	*Clarias_MHCI*TH5,16,22*	821T→G	Silent	Gly to Gly (GGT to GGG)
7	*Clarias_MHCI*TH1,2,11-15,17-21,24-40,42,44,47,49,50,54,55,57-59,61-68,70,73,74,76,77-80, 83-85,87,90,91*	821T→C	Silent	Gly to Gly (GGT to GGC)
8	*Clarias_MHCI*TH81,82,86*	821T→A	Missense	Gly to Val (GGT to GTA)
9	*Clarias_MHCI*TH3,10,69,71*	821T→A	Silent	Gly to Gly (GGT to GGA)
10	*Clarias_MHCI*TH73,81*	822T→C	Missense	Ser to Arg (TCT to CGG)
11	*Clarias_MHCI*TH1,2,4-9,11-19,21-27,29-31,33,34,36-43,45-53,55-68,70,72,74-80,82-90*	823C→G	Missense	Ser to Trp (TCT to TGG)
12	*Clarias_MHCI*TH20,32,35,44,54*	823C→G	Nonsense	Ser to Stop (TCT to TGA)
13	*Clarias_MHCI*TH73,81*	822T→C	Missense	Ser to Arg (TCT to CGG)
14	*Clarias_MHCI*TH3,10,28,69,71,91*	823C→A	Nonsense	Ser to Stop (TCT to TAG)
15	*Clarias_MHCI*TH82,86*	825A→T	Missense	Ile to Phe (ATC to TTT)
16	*Clarias_MHCI*TH57*	825A→G	Missense	Ile to Val (ATC to GTA)
17	*Clarias_MHCI*TH81*	825A→C	Missense	Ile to Leu (ATC to CTG)
18	*Clarias_MHCI*TH39*	827C→T	Silent	Ile to Ile (ATC to ATT)
19	*Clarias_MHCI*TH82,86*	827C→T	Missense	Ile to Phe (ATC to TTT)
20	*Clarias_MHCI*TH81*	827C→G	Missense	Ile to Leu (ATC to CTG)
21	*Clarias_MHCI*TH57*	827C→A	Missense	Ile to Val (ATC to GTA)
22	*Clarias_MHCI*TH1-38,40-56,58-80,83-85,87-91*	827C→A	Silent	Ile to Ile (ATC to ATA)
23	*Clarias_MHCI*TH47,61,62,64-66,68,70*	828C→T	Nonsense	Leu to Stop (CTA to TGA)
24	*Clarias_MHCI*TH1-17,21-25,27,28,31,33,35-46,48-52,55-59,63,67,69,71-79,83-85,87-91*	828C→T	Silent	Leu to Leu (CTA to TTG)
25	*Clarias_MHCI*TH18,19,26,29,32,34,60,80*	828C→T	Silent	Leu to Leu (CTA to TTA)
26	*Clarias_MHCI*TH20*	828C→T	Missense	Leu to Phe (CTA to TTT)
27	*Clarias_MHCI*TH54,81*	828C→T	Missense	Leu to Cys (CTA to TGT)
28	*Clarias_MHCI*TH82,86*	828C→T	Missense	Leu to Cys (CTA to TGC)
29	*Clarias_MHCI*TH47,61,62,64-66,68,70*	829T→G	Nonsense	Leu to Stop (CTA to TGA)
30	*Clarias_MHCI*TH54,81*	829T→G	Missense	Leu to Cys (CTA to TGT)

No	Allele-specific	Variable nucleotide position and nucleotide substitution	Mutation type	Amino acid change (substitution)
31	*Clarias_MHCI*TH82,86*	829T→G	Missense	Leu to Cys (CTA to TGC)
32	*Clarias_MHCI*TH20*	830A→T	Missense	Leu to Phe (CTA to TTT)
33	*Clarias_MHCI*TH54,81*	830A→T	Missense	Leu to Cys (CTA to TGT)
34	*Clarias_MHCI*TH1-17,21-25,27,28,30,31,33,35-46,48-52,55-59,63,67,69,71-79,83-85,87-91*	830A→G	Silent	Leu to Leu (CTA to TTG)
35	*Clarias_MHCI*TH53*	830A→G	Silent	Leu to Leu (CTA to CTG)
36	*Clarias_MHCI*TH82,86*	830A→C	Missense	Leu to Cys (CTA to TGC)
37	*Clarias_MHCI*TH25,38*	834A→G	Missense	Met to Val (ATG to GTT)
38	*Clarias_MHCI*TH3-10,17,20,27,32,35,44-53,55-72,74,77,78,80,83,84,85,87-90*	834A→G	Missense	Met to Val (ATG to GTG)
39	*Clarias_MHCI*TH79*	834A→G	Missense	Met to Val (ATG to GTA)
40	*Clarias_MHCI*TH12,18,19,21,23,24,26,28,29-31,33,34,36,40,41,43,81,91*	834A→G	Missense	Met to Gly (ATG to GGG)
41	*Clarias_MHCI*TH1,2,11,13,15,16,22,37,69,42*	834A→G	Missense	Met to Gly (ATG to GGA)
42	*Clarias_MHCI*TH14,82,86*	834A→G	Missense	Met to Ala (ATG to GCC)
43	*Clarias_MHCI*TH12,18,19,21,23,24,26,28-31,33,34,36,40,41,43,81,91*	835T→G	Missense	Met to Gly (ATG to GGG)
44	*Clarias_MHCI*TH1,2,11,13,15,16,22,37,39,42*	835T→G	Missense	Met to Gly (ATG to GGA)
45	*Clarias_MHCI*TH14,82,86*	835T→C	Missense	Met to Ala (ATG to GCG)
46	*Clarias_MHCI*TH25,38*	836G→T	Missense	Met to Val (ATG to GTT)
47	*Clarias_MHCI*TH82,86*	836G→C	Missense	Met to Ala (ATG to GCC)
48	*Clarias_MHCI*TH79*	836G→A	Missense	Met to Val (ATG to GTA)
49	*Clarias_MHCI*TH1,2,11,13,15,16,22,37,39,42*	836G→A	Missense	Met to Gly (ATG to GGA)
50	*Clarias_MHCI*TH4,8,48,88,89*	838G→T	Missense	Trp to Leu (TGG to TTA)
51	*Clarias_MHCI*TH67,82,86*	838G→C	Missense	Trp to Ser (TGG to TCA)
52	*Clarias_MHCI*TH1,2,11,13,15,16,22,37,39,42*	838G→A	Nonsense	Trp to Stop (TGG to TAA)
53	*Clarias_MHCI*TH3,5-7,9,10,12,14,17-21,23-36,38,40,41,43-47,49-66,68-81,83-85,87,90,91*	839G→A	Nonsense	Trp to Stop (TGG to TGA)
54	*Clarias_MHCI*TH1,2,11,13,15,16,22,37,39,42*	839G→A	Nonsense	Trp to Stop (TGG to TAA)
55	*Clarias_MHCI*TH67,82,86*	839G→A	Missense	Trp to Ser (TGG to TCA)
56	*Clarias_MHCI*TH4,8,48,88,89*	839G→A	Missense	Trp to Leu (TGG to TTA)
57	*Clarias_MHCI*TH1-81,83,85,87-91*	840A→T	Nonsense	Arg to Stop (AGA to TGA)
58	*Clarias_MHCI*TH82,84,86*	840A→G	Missense	Arg to Gly (AGA to GGA)
59	*Clarias_MHCI*TH65,66,81,82,86*	845A→T	Missense	Stop to Cys (TGA to TGT)
60	*Clarias_MHCI*TH47,61,62,64,68,70,90*	845A→G	Missense	Stop to W (TGA to TGG)
61	*Clarias_MHCI*TH45*	845A→C	Missense	Stop to Cys (TGA to TGC)
62	*Clarias_MHCI*TH1-25,27-60,62-68,70-79,83-85,87-91*	853G→T	Missense	Arg to Ile (AGA to ATA)
63	*Clarias_MHCI*TH81*	853G→C	Missense	Arg to Thr (AGA to ACA)
64	*Clarias_MHCI*TH26,69,80,82,86*	853G→A	Missense	Arg to Lys (AGA to AAA)

No	Allele-specific	Variable nucleotide position and nucleotide substitution	Mutation type	Amino acid change (substitution)
65	*Clarias_MHCI*TH82,86*	863A→T	Silent	Leu to Leu (CTA to CTT)
66	*Clarias_MHCI*TH1,2,4,8,10-44,47-50,53-55,57-68,70,73,74,76,77,79,80,83-85,87-91*	863A→G	Silent	Leu to Leu (CTA to CTG)
67	*Clarias_MHCI*TH72,78*	863A→C	Silent	Leu to Leu (CTA to CTC)
68	*Clarias_MHCI*TH1,2,4-24,26-,7,39-68,70,72,84,86-91*	866T→C	Silent	Pro to Pro (CCT to CCC)
69	*Clarias_MHCI*TH25,38*	866T→A	Silent	Pro to Pro (CCT to CCA)
70	*Clarias_MHCI*TH81*	871T→C	Missense	Ile to Thr (ATC to ACG)
71	*Clarias_MHCI*TH1-29,31-80,82-87,89-91*	871T→C	Missense	Ile to Thr (ATC to ACA)
72	*Clarias_MHCI*TH88*	871T→A	Missense	Ile to Lys (ATC to AAA)
73	*Clarias_MHCI*TH81*	872C→G	Missense	Ile to Thr (ATC to ACG)
74	*Clarias_MHCI*TH1-29,31-80,82-87,89-91*	872C→A	Missense	Ile to Thr (ATC to ACA)
75	*Clarias_MHCI*TH88*	872C→A	Missense	Ile to Lys (ATC to AAA)
76	*Clarias_MHCI*TH30*	872C→A	Silent	Ile to Ile (ATC to ATA)
77	*Clarias_MHCI*TH49,55,74,77,80*	874G→T	Missense	Trp to Leu (TGG to TTG)
78	*Clarias_MHCI*TH82,86*	874G→C	Missense	Trp to Ser (TGG to TCT)
79	*Clarias_MHCI*TH78,81*	874G→C	Missense	Trp to Ser (TGG to TCG)
80	*Clarias_MHCI*TH82,86*	875G→T	Missense	Trp to Ser (TGG to TCT)
81	*Clarias_MHCI*TH4,8,48,88,89*	875G→T	Missense	Trp to Cys (TGG to TGT)
82	*Clarias_MHCI*TH27,54*	875G→A	Nonsense	Trp to Stop (TGG to TGA)
83	*Clarias_MHCI*TH81*	876T→G	Missense	Stop to Glu (TAA to GAG)
84	*Clarias_MHCI*TH25*	877A→C	Missense	Stop to Ser (TAA to TCA)
85	*Clarias_MHCI*TH38*	877A→C	Missense	Stop to Ser (TAA to TCG)
86	*Clarias_MHCI*TH1-16,17-24,26-37,39-72,74,75,77-91*	878A→G	Silent	Stop to Stop (TAA to TAG)
87	*Clarias_MHCI*TH38*	878A→G	Missense	Stop to Ser (TAA to TCG)
88	*Clarias_MHCI*TH81*	878A→G	Missense	Stop to Glu (TAA to GAG)
89	*Clarias_MHCI*TH17*	878A→C	Missense	Stop to Tyr (TAA to TAC)
90	*Clarias_MHCI*TH52,58,79,83,84*	879C→T	Missense	His to Tyr (CAT to TAT)
91	*Clarias_MHCI*TH1-17,20-23,25,27-33,35-51,53,55-57,59-79,80,81,88-90*	879C→G	Missense	His to Asp (CAT to GAT)
92	*Clarias_MHCI*TH18,19,24,26,34,54,85,87*	879C→A	Missense	His to Asn (CAT to AAT)
93	*Clarias_MHCI*TH81*	882C→T	Missense	Leu to Phe (CTT to TTT)
94	*Clarias_MHCI*TH1,2,4-9,11-15,18-21,23-26,28-45,47-49,51-57,60-68,70,72,74,75,77,78,80,8587,88,91*	882C→G	Missense	Leu to Val (CTT to GTT)
95	*Clarias_MHCI*TH82,86*	882C→G	Missense	Leu to Gly (CTT to GGT)
96	*Clarias_MHCI*TH3,10,16,17,22,27,59,69,71,73,76,89*	882C→G	Missense	Leu to Ala (CTT to GCT)
97	*Clarias_MHCI*TH3,10,16,17,22,27,59,69,71,73,76,89*	883T→C	Missense	Leu to Ala (CTT to GCT)
98	*Clarias_MHCI*TH46,50,90*	882C→A	Missense	Leu to Ile (CTT to ATT)

No	Allele-specific	Variable nucleotide position and nucleotide substitution	Mutation type	Amino acid change (substitution)
99	*Clarias_MHCI*TH82,86*	883T→G	Missense	Leu to Gly (CTT to GGT)
100	*Clarias_MHCI*TH1,2,5,7,9,10,11-16,18-25,26,28-39,41-47,50-53,56-68,70,72,73,75-80,82-87,90,91*	899C→G	Silent	Pro to Pro (CCC to CCG)
101	*Clarias_MHCI*TH40,54,81*	899C→A	Silent	Pro to Pro (CCC to CCA)
102	*Clarias_MHCI*TH64-66*	900A→C	Missense	Ser to Arg (AGT to CGA)
103	*Clarias_MHCI*TH81*	901G→T	Missense	Ser to Met (AGT to ATG)
104	*Clarias_MHCI*TH3,10,17,27,69,71*	901G→T	Missense	Ser to Ile (AGT to ATT)
105	*Clarias_MHCI*TH30*	901G→T	Missense	Ser to Ile (AGT to ATA)
106	*Clarias_MHCI*TH82,86*	901G→C	Missense	Ser to Thr (AGT to ACA)
107	*Clarias_MHCI*TH4,8,48,88,89*	901G→A	Missense	Ser to Lys (AGT to AAA)
108	*Clarias_MHCI*TH81*	902T→G	Missense	Ser to Met (AGT to ATG)
109	*Clarias_MHCI*TH82,86*	902T→A	Missense	Ser to Thr (AGT to ACA)
110	*Clarias_MHCI*TH1,2,5-7,9,11-16,18-26,28,29,31-47,49-63,67,68,70,72-80,83-85,87,90,91*	902T→A	Missense	Ser to Arg (AGT to AGA)
111	*Clarias_MHCI*TH4,8,48,88,89*	902T→A	Missense	Ser to Lys (AGT to AAA)
112	*Clarias_MHCI*TH30*	902T→A	Missense	Ser to Ile (AGT to ATA)
113	*Clarias_MHCI*TH58,79,82-84,86*	903G→T	Missense	Gly to Tyr (GGT to TAT)
114	*Clarias_MHCI*TH21,24,28,29,31,33,40,41,81,91*	903G→C	Missense	Gly to His (GGT to CAT)
115	*Clarias_MHCI*TH18,19,26,34*	903G→C	Missense	Gly to His (GGT to CAC)
116	*Clarias_MHCI*TH1-17,20,22,23,25,27,30,32,35-39,42-52,54-57,59-62,64-78,80,85,87-90*	903G→A	Missense	Gly to Asn (GGT to AAT)
117	*Clarias_MHCI*TH53,63*	903G→A	Missense	Gly to Lys (GGT to AAG)
118	*Clarias_MHCI*TH58,79,82-84,86*	904G→A	Missense	Gly to Tyr (GGT to TAT)
119	*Clarias_MHCI*TH1-17,20,22,23,25,27,30,32,35-39,42-52,54-57,59-62,64-78,80,85,87-90*	904G→A	Missense	Gly to Asn (GGT to AAT)
120	*Clarias_MHCI*TH53,63*	904G→A	Missense	Gly to Lys (GGT to AAG)
121	*Clarias_MHCI*TH21,24,28,29,31,33,40,41,81,91*	904G→A	Missense	Gly to His (GGT to CAT)
122	*Clarias_MHCI*TH18,19,26,34*	904G→A	Missense	Gly to His (GGT to CAC)
123	*Clarias_MHCI*TH53,63*	905T→G	Missense	Gly to Lys (GGT to AAG)
124	*Clarias_MHCI*TH18,19,26,34*	905T→C	Missense	Gly to His (GGT to CAC)
125	*Clarias_MHCI*TH1-30,32-42,44-49,51-53,55-57,60-78,85,87,89,91*	906G→T	Missense	Asp to Ser (GAT to TCT)
126	*Clarias_MHCI*TH82,86*	906G→T	Missense	Asp to Ser (GAT to TCC)
127	*Clarias_MHCI*TH81*	906G→T	Missense	Asp to Leu (GAT to TTA)
128	*Clarias_MHCI*TH50,58,79,83,84,90*	906G→T	Missense	Asp to Phe (GAT to TTT)
129	*Clarias_MHCI*TH31,54*	906G→C	Missense	Asp to Pro (GAT to CCT)
130	*Clarias_MHCI*TH59*	906G→C	Missense	Asp to His (GAT to CAT)
131	*Clarias_MHCI*TH43*	906G→A	Missense	Asp to Thr (GAT to ACT)
132	*Clarias_MHCI*TH81*	907A→T	Missense	Asp to Leu (GAT to TTA)

No	Allele-specific	Variable nucleotide position and nucleotide substitution	Mutation type	Amino acid change (substitution)
133	*Clarias_MHCI*TH50,58,79,83,84,90*	907A→T	Missense	Asp to Phe (GAT to TTT)
134	*Clarias_MHCI*TH43*	907A→C	Missense	Asp to Thr (GAT to ACT)
135	*Clarias_MHCI*TH1-30,32-42,44-49,51-53,55-57,60-78,85,87,89,91*	907A→C	Missense	Asp to Ser (GAT to TCT)
136	*Clarias_MHCI*TH82,86*	907A→C	Missense	Asp to Ser (GAT to TCC)
137	*Clarias_MHCI*TH31,54*	907A→C	Missense	Asp to Pro (GAT to CCT)
138	*Clarias_MHCI*TH4,48,88*	909G→A	Missense	Gly to Arg (GGA to AGA)
139	*Clarias_MHCI*TH81*	910G→C	Missense	Gly to Ala (GGA to GCA)
140	*Clarias_MHCI*TH19,33*	910G→A	Missense	Gly to Glu (GGA to GAA)
141	*Clarias_MHCI*TH82,86*	910G→A	Missense	Gly to Asp (GGA to GAT)
142	*Clarias_MHCI*TH82,86*	911A→T	Missense	Gly to Asp (GGA to GAT)
143	*Clarias_MHCI*TH5-7,9,45,46,51,52,56-58,72,75,77,79,83,84*	911A→G	Silent	Gly to Gly (GGA to GGG)
144	*Clarias_MHCI*TH13,67,80*	912A→T	Missense	Ile to Leu (ATC to TTG)
145	*Clarias_MHCI*TH81*	912A→G	Missense	Ile to Val (ATC to GTT)
146	*Clarias_MHCI*TH1-4,8,10-12,14-27,29,31,32,34-37,40-44,47-50,53,55,60-66,68-74,76,77,85,87-90*	912A→G	Missense	Ile to Val (ATC to GTG)
147	*Clarias_MHCI*TH28,30,57,91*	912A→G	Missense	Ile to Val (ATC to GTA)
148	*Clarias_MHCI*TH82,86*	912A→G	Missense	Ile to Asp (ATC to GAT)
149	*Clarias_MHCI*TH58,79,83,84*	912A→C	Missense	Ile to Leu (ATC to CTG)
150	*Clarias_MHCI*TH33*	913T→G	Missense	Ile to Arg (ATC to AGG)
151	*Clarias_MHCI*TH54*	913T→A	Missense	Ile to Lys (ATC to AAG)
152	*Clarias_MHCI*TH82,86*	913T→A	Missense	Ile to Asp (ATC to GAT)
153	*Clarias_MHCI*TH81*	914C→T	Missense	Ile to Val (ATC to GTT)
154	*Clarias_MHCI*TH82,86*	914C→T	Missense	Ile to Asp (ATC to GAT)
155	*Clarias_MHCI*TH1-4,8,10-12,14-27,29,31,32,34-37,40-44,47-50,53,55,60-66,68-74,76,77,85,87-90*	914C→G	Missense	Ile to Val (ATC to GTG)
156	*Clarias_MHCI*TH33*	914C→G	Missense	Ile to Arg (ATC to AGG)
157	*Clarias_MHCI*TH5-7,9,45,46,51,52,56,72,75,78*	914C→G	Missense	Ile to Met (ATC to ATG)
158	*Clarias_MHCI*TH13,67,80*	914C→G	Missense	Ile to Leu (ATC to TTG)
159	*Clarias_MHCI*TH58,79,83,84*	914C→G	Missense	Ile to Leu (ATC to CTG)
160	*Clarias_MHCI*TH54*	914C→G	Missense	Ile to Lys (ATC to AAG)
161	*Clarias_MHCI*TH28,30,57,91*	914C→A	Missense	Ile to Val (ATC to GTA)
162	*Clarias_MHCI*TH82,86*	915T→C	Missense	Tyr to Arg (TAC to CGT)
163	*Clarias_MHCI*TH1-20,22,23,26-28,30,32,34-37,39,42-77,79,83-85,87-91*	915T→A	Missense	Tyr to Ser (TAC to AGT)
164	*Clarias_MHCI*TH21,24,29,31,40,41,80*	915T→A	Missense	Tyr to Asn (TAC to AAT)
165	*Clarias_MHCI*TH25,38*	915T→A	Missense	Tyr to Lys (TAC to AAG)
166	*Clarias_MHCI*TH33*	915T→A	Missense	Tyr to Lys (TAC to AAA)

No	Allele-specific	Variable nucleotide position and nucleotide substitution	Mutation type	Amino acid change (substitution)
167	*Clarias_MHCI*TH78*	915T→A	Missense	Tyr to Ile (TAC to ATT)
168	*Clarias_MHCI*TH81*	915T→A	Missense	Tyr to Ile (TAC to ATC)
169	*Clarias_MHCI*TH82,86*	916A→G	Missense	Tyr to Arg (TAC to CGT)
170	*Clarias_MHCI*TH1-20,22,23,26-28,30,32,34-37,39,42-77,79,83-85,87-91*	916A→G	Missense	Tyr to Ser (TAC to AGT)
171	*Clarias_MHCI*TH78*	916A→T	Missense	Tyr to Ile (TAC to ATT)
172	*Clarias_MHCI*TH81*	916A→T	Missense	Tyr to Ile (TAC to ATC)
173	*Clarias_MHCI*TH82,86*	917C→T	Missense	Tyr to Arg (TAC to CGT)
174	*Clarias_MHCI*TH1-20,22,23,26-28,30,32,34-37,39,42-77,79,83-85,87-91*	917C→T	Missense	Tyr to Ser (TAC to AGT)
175	*Clarias_MHCI*TH21,24,29,31,40,41,80*	917C→T	Missense	Tyr to Asn (TAC to AAT)
176	*Clarias_MHCI*TH25,38*	917C→G	Missense	Tyr to Lys (TAC to AAG)
177	*Clarias_MHCI*TH33*	917C→A	Missense	Tyr to Lys (TAC to AAA)
178	*Clarias_MHCI*TH78*	917C→T	Missense	Tyr to Ile (TAC to ATT)
179	*Clarias_MHCI*TH1-24,26-37,39-58,60,62,63,65-69,71-80,83-85,97-91*	918C→T	Nonsense	Gln to Stop (CAG to TAG)
180	*Clarias_MHCI*TH61,64,68,70*	918C→T	Nonsense	Gln to Stop (CAG to TAA)
181	*Clarias_MHCI*TH25,38*	918C→A	Missense	Gln to Asn (CAG to AAT)
182	*Clarias_MHCI*TH59*	918C→A	Missense	Gln to Lys (CAG to AAG)
183	*Clarias_MHCI*TH61,64,68,70*	920G→A	Nonsense	Gln to Stop (CAG to TAA)
184	*Clarias_MHCI*TH25,38*	920G→T	Missense	Gln to Asn (CAG to AAT)
185	*Clarias_MHCI*TH81*	920G→C	Missense	Gln to His (CAG to CAC)
186	*Clarias_MHCI*TH49,55,74,80*	921C→T	Silent	Leu to Leu (CTA to TTA)
187	*Clarias_MHCI*TH1-48,50-54,56,58-73,75-79,81-91*	921C→A	Missense	Leu to Ile (CTA to ATA)
188	*Clarias_MHCI*TH1,2,4-9,11-16,18-24,26,28-35,37,39-68,70,72-81,83-85,87-91*	924G→T	Nonsense	Gly to Stop (GGA to TGA)
189	*Clarias_MHCI*TH82,86*	924G→C	Missense	Gly to Arg (GGA to CGA)
190	*Clarias_MHCI*TH3,10,17,25,27,36,38,69,71*	924G→A	Missense	Gly to Arg (GGA to AGA)
191	*Clarias_MHCI*TH81*	928G→T	Missense	Arg to Ile (AGA to ATT)
192	*Clarias_MHCI*TH81*	928G→A	Missense	Arg to Asn (AGA to AAT)
193	*Clarias_MHCI*TH5,9,60,72*	928G→A	Missense	Arg to Asn (AGA to AAT)
194	*Clarias_MHCI*TH5,9,60,72*	929A→T	Missense	Arg to Asn (AGA to AAT
195	*Clarias_MHCI*TH1-4,6-8,10-20,22,23,25-28,30,32,34-36,38,39,42-59,61-71,73-79,80,83-85,87-91*	929A→T	Missense	Arg to Ser (AGA to AGT)
196	*Clarias_MHCI*TH21,24,29,31,33,37,40,41*	929A→G	Missense	Arg to Arg (AGA to AGG)
197	*Clarias_MHCI*TH58,79,81*	947A→T	Silent	Thr to Thr (ACA to ACT)
198	*Clarias_MHCI*TH49,55*	947A→G	Silent	Thr to Thr (ACA to ACG)
199	*Clarias_MHCI*TH59*	948A→T	Nonsense	Arg to Stop (AGG to TAG)
200	*Clarias_MHCI*TH59*	949G→A	Nonsense	Arg to Stop (AGG to TAG)

No	Allele-specific	Variable nucleotide position and nucleotide substitution	Mutation type	Amino acid change (substitution)
201	*Clarias_MHCI*TH47,54,64,-66,68*	948A→G	Missense	Arg to Gly (AGG to GGG)
202	*Clarias_MHCI*TH1,2,4-9,11-16,18-26,28-46,48,49,51-53,56-59,60-63,67,70,72,74,75,79-81,83-91*	948A→G	Missense	Arg to Glu (AGG to GAG)
203	*Clarias_MHCI*TH1,2,4-9,11-16,18-26,28-46,48,49,51-53,56-59,60-63,67,70,72,74,75,79-81,83-91*	949G→A	Missense	Arg to Glu (AGG to GAG)
204	*Clarias_MHCI*TH55*	948A→C	Missense	Arg to Gln (AGG to CAG)
205	*Clarias_MHCI*TH55*	949G→A	Missense	Arg to Gln (AGG to CAG)
206	*Clarias_MHCI*TH3,10,17,27,43,50,69,71,76,76*	949G→A	Missense	Arg to Lys (AGG to AAG)
207	*Clarias_MHCI*TH82,86*	949G→T	Missense	Arg to Met (AGG to ATG)
208	*Clarias_MHCI*TH90*	949G→A	Missense	Arg to Lys (AGG to AAG)
209	*Clarias_MHCI*TH2-15,17-21,23-29,31-66,68-77,79-81,83-85,87-91*	952C→T	Missense	Thr to Ile (ACA to ATA)
210	*Clarias_MHCI*TH1,16,22,30*	952C→G	Missense	Thr to Arg (ACA to AGA)
211	*Clarias_MHCI*TH67,82,86*	952C→A	Missense	Thr to Lys (ACA to AAA)
212	*Clarias_MHCI*TH30*	955A→T	Missense	Lys to Met (AAG to ATG)
213	*Clarias_MHCI*TH1,3-11,13,16,18,19,21-24,26,28,29,31,33,34,37,41,43,45-77,79,80,83-85,87-91*	955A→G	Missense	Lys to Arg (AAG to AGG)
214	*Clarias_MHCI*TH82,86*	955A→C	Missense	Lys to Thr (AAG to ACG)
215	*Clarias_MHCI*TH1-17,18-21,23-26,28-52,53-56,58-62,64-66,68-77,79-81,83-85,87-91*	957A→G	Missense	Ile to Val (ATC to GTC)
216	*Clarias_MHCI*TH1-16,18-24,26,28-37,39,40,41,43-46,48-58,61,63,67,69,71-80,83-85,87-91*	963A→G	Missense	Met to Val (ATG to GTG)
217	*Clarias_MHCI*TH5,7,9,39,45,51,52,56,72,75*	968T→C	Silent	Ile to Ile (ATT to ATC)
218	*Clarias_MHCI*TH82,86*	968T→A	Silent	Ile to Ile (ATT to ATA)
219	*Clarias_MHCI*TH1-19,21-31,33,34,36-43,45-91*	974G→A	Missense	Met to Ile (ATG to ATA)
220	*Clarias_MHCI*TH3,69,71*	976G→T	Missense	Cys to Phe (TGT to TTT)
221	*Clarias_MHCI*TH1,2,4-68,70,72-80,82-91*	976G→A	Missense	Cys to Tyr (TGT to TAT)
222	*Clarias_MHCI*TH1-17,20-25,27-33,35-80,83-85,87-91*	978C→T	Missense	Leu to Phe (CTC to TTC)
223	*Clarias_MHCI*TH19,26,34,82,86*	978C→G	Missense	Leu to Val (CTC to GTC)
224	*Clarias_MHCI*TH18*	978C→A	Missense	Leu to Ile (CTC to ATC)
225	*Clarias_MHCI*TH5,9,56,72*	984G→C	Missense	Ala to Pro (GCA to CCA)
226	*Clarias_MHCI*TH43,81*	984G→A	Missense	Ala to Lys (GCA to AAA)
227	*Clarias_MHCI*TH43,81*	985C→A	Missense	Ala to Lys (GCA to AAA)
228	*Clarias_MHCI*TH91*	985C→T	Missense	Ala to Val (GCA to GTA)
229	*Clarias_MHCI*TH1-4,6-8,-10-17,20-23,25,27-33,35-39,41,42,44-46,48-55,57-60,63,67,69,73-80,83-90*	985C→G	Missense	Ala to Gly (GCA to GGA)
230	*Clarias_MHCI*TH18,19,24,26,34,40,47,61,62,64-66,68,70,82,86*	985C→A	Missense	Ala to Glu (GCA to GAA)
231	*Clarias_MHCI*TH80,81*	987C→T	Missense	Pro to Tyr (CCT to TAT)
232	*Clarias_MHCI*TH36,48,60,71,88*	987C→A	Missense	Pro to Asn (CCT to AAT)
233	*Clarias_MHCI*TH80,81*	988C→A	Missense	Pro to Tyr (CCT to TAT)
234	*Clarias_MHCI*TH36,48,60,71,88*	988C→A	Missense	Pro to Asn (CCT to AAT)

No	Allele-specific	Variable nucleotide position and nucleotide substitution	Mutation type	Amino acid change (substitution)
235	*Clarias_MHCI*TH82,86*	988C→A	Missense	Pro to Gln (CCT to CAG)
236	*Clarias_MHCI*TH1-35,37-47,49-59,61-70,72-79,83-85,87,89-91*	988C→A	Missense	Pro to His (CCT to CAT)
237	*Clarias_MHCI*TH82,86*	989T→G	Missense	Pro to Gln (CCT to CAG)
238	*Clarias_MHCI*TH1-16,18,19,21-26,28-31,33,34,36-43,45-80,83-85,87-91*	990T→G	Missense	Leu to Val (TTA to GTT)
239	*Clarias_MHCI*TH81*	990T→C	Missense	Leu to Gln (TTA to CAG)
240	*Clarias_MHCI*TH17,27,80*	990T→C	Silent	Leu to Leu (TTA to CTT)
241	*Clarias_MHCI*TH82*	990T→C	Missense	Leu to His (TTA to CAC)
242	*Clarias_MHCI*TH20,32,35,44*	990T→A	Missense	Leu to Ile (TTA to ATT)
243	*Clarias_MHCI*TH86*	991T→A	Missense	Leu to Tyr (TTA to TAC)
244	*Clarias_MHCI*TH81*	991T→A	Missense	Leu to Gln (TTA to CAG)
245	*Clarias_MHCI*TH82*	991T→A	Missense	Leu to His (TTA to CAC)
246	*Clarias_MHCI*TH1-16,18,19,21-26,28-31,33,34,36-43,45-80,83-85,87-91*	992A→T	Missense	Leu to Val (TTA to GTT)
247	*Clarias_MHCI*TH81*	992A→G	Missense	Leu to Gln (TTA to CAG)
248	*Clarias_MHCI*TH17,27,80*	992A→T	Silent	Leu to Leu (TTA to CTT)
249	*Clarias_MHCI*TH82*	992A→C	Missense	Leu to His (TTA to CAC)
250	*Clarias_MHCI*TH20,32,35,44*	992A→T	Missense	Leu to Ile (TTA to ATT)
251	*Clarias_MHCI*TH86*	992A→C	Missense	Leu to Tyr (TTA to TAC)
252	*Clarias_MHCI*TH53,60,63*	993A→T	Missense	Lys to Trp (AAG to TGG)
253	*Clarias_MHCI*TH1-52,54-59,61,62,64,81,83-85,87-91*	993A→G	Missense	Lys to Gly (AAG to GGG)
254	*Clarias_MHCI*TH82*	993A→C	Missense	Lys to Gln (AAG to CAG)
255	*Clarias_MHCI*TH86*	993A→C	Missense	Lys to Leu (AAG to CTG)
256	*Clarias_MHCI*TH53,60,63*	994A→G	Missense	Lys to Trp (AAG to TGG)
257	*Clarias_MHCI*TH1-52,54-59,61,62,64,81,83-85,87-91*	994A→G	Missense	Lys to Gly (AAG to GGG)
258	*Clarias_MHCI*TH86*	994A→T	Missense	Lys to Leu (AAG to CTG)
